# Identification of a Candidate Gene for Panicle Length in Rice (*Oryza sativa* L.) Via Association and Linkage Analysis

**DOI:** 10.3389/fpls.2016.00596

**Published:** 2016-05-03

**Authors:** Erbao Liu, Yang Liu, Guocan Wu, Siyuan Zeng, Thu G. Tran Thi, Lijun Liang, Yinfeng Liang, Zhiyao Dong, Dong She, Hui Wang, Imdad U. Zaid, Delin Hong

**Affiliations:** ^1^State Key Laboratory of Crop Genetics and Germplasm Enhancement, Nanjing Agricultural UniversityNanjing, China; ^2^College of Agronomy, Hue University of Agriculture and Forestry, Hue UniversityHue, Vietnam

**Keywords:** association analysis, gene identification, map-based cloning, panicle length, quantitative trait locus, rice

## Abstract

Panicle length (PL) is an important trait for improving panicle architecture and grain yield in rice (*Oryza sativa* L.). Three populations were used to identify QTLs and candidate genes associated with PL. Four QTLs for PL were detected on chromosomes 4, 6, and 9 through linkage mapping in the recombinant inbred line population derived from a cross between the cultivars Xiushui79 (short panicle) and C-bao (long panicle). Ten SSR markers associated with PL were detected on chromosomes 2, 3, 5, 6, 8, 9, and 10 in the natural population consisting of 540 accessions collected from East and Southeast Asia. A major locus on chromosome 9 with the largest effect was identified via both linkage and association mapping. *LONG PANICLE 1* (*LP1*) locus was delimited to a 90-kb region of the long arm of chromosome 9 through fine mapping using a single segment segregating F_2_ population. Two single nucleotide polymorphisms (SNPs) leading to amino acid changes were detected in the third and fifth exons of *LP1*. *LP1* encodes a Remorin_C-containing protein of unknown function with homologs in a variety of species. Sequencing analysis of *LP1* in two parents and 103 rice accessions indicated that SNP1 is associated with panicle length. The *LP1* allele of Xiushui79 leads to reduced panicle length, whereas the allele of C-bao relieves the suppression of panicle length. *LP1* and the elite alleles can be used to improve panicle length in rice.

## Introduction

Rice (*Oryza sativa* L.) is an important staple food that feeds approximately 50% of the world's population. At present, rice is grown globally on approximately 160 million hectares annually and the average yield is 4.4 tons per hectare (GRiSP, 2013). Higher productivity is needed to meet the demands of the rapidly increasing population (Yuan, 1997; Khush, 1999). Panicle length is one aspect of panicle architecture and is usually measured as a yield-related trait. Panicle length, together with spikelet number and density, seed setting rate and grain plumpness, determines the grain number per panicle; hence, yield increases in rice. Studies focusing on traits that are components of grain yield and quality, such as grain number, panicle number and grain weight, have revealed a few genes associated with these traits, such as *GS3, GS5, GW2, GW5, GW8, Gn1, GL3*, and *GIF1* (Ashikari et al., 2005; Fan et al., 2006; Song et al., 2007; Wang et al., 2008, 2012; Weng et al., 2008; Li Y. et al., 2011; Zhang et al., 2012). Panicle length QTL were commonly co-identified with heading date, and some genes were cloned in recent years, such as *Hd1, EHD4, Hd6, Ghd7, DTH7*, and *DTH8* (Yano et al., 2000; Takahashi et al., 2001; Xue et al., 2008; Wei et al., 2010; Gao et al., 2013, 2014). However, panicle length has received relatively less attention. There are two subspecies, i.e., indica and japonica, in *Oryza sativa* L. In general, subspecies indica has longer panicle length and looser spikelet density than those of japonica. Even within subspecies there is considerable genetic variation of panicle length (range, 12–40 cm; genetic coefficient of variation, 10%; Jambhulkar and Bose, 2014; Zuo et al., 2014). Panicle length is inherited in a quantitative manner and controlled by both major and minor QTLs (Liu et al., 2011). To date, at least 253 QTLs for panicle length have been detected distributed on 12 chromosomes (Xiao et al., 1998; Hittalmani et al., 2002, 2003; Xing et al., 2002; Kobayashi et al., 2003; Thomson et al., 2003; Ashikari et al., 2005; Lee et al., 2005; Mei et al., 2005; Cho et al., 2007; Liu et al., 2011; Marathi et al., 2012; Yao et al., 2015; Zhang et al., 2015). However, only a few genes have been cloned and applied in rice plant architecture breeding. The gene *short panicle1* (*SP1*) encodes a putative polypeptide transporter protein (PTR) family regulating the activity of the spike meristem, resulting in the short-panicle phenotype. SP1 contains a conserved PTR2 domain consisting of 12 transmembrane domains (Li et al., 2009). The *dense and erect panicle1* (*DEP1*) locus is a gain-of-function mutation that results in the truncation of a phosphatidylethanolamine-binding protein-like domain protein. This allele enhances meristematic activity, resulting in a reduced length of the inflorescence internode, an increased number of grains per panicle and a consequent increase in grain yield (Huang et al., 2009). The gene *DEP2* encodes a plant-specific protein without any known functional domain is essential for determining panicle outgrowth and elongation (Li et al., 2010). And the gene *DEP3*, predicted to encode a patatin-like phospholipase A2, plays an important role in high grain yield (Qiao et al., 2011). *LARGER PANICLE* (*LP*) encodes a Kelch repeat-containing F-box protein and leads to an increase in spikelets and branches. *LP* expression is enriched in the branch primordial region, and *LP* may be involved in modulating cytokinin levels in plant tissues (Li M. et al., 2011).

Linkage mapping using cross-populations is the traditional method for QTL identification due to its high power and simple genetic background (Huang and Han, 2014). However, linkage mapping exploits only those loci with the strongest influence, hindering the detection of phenotypes occurring at lower frequencies in samples of natural populations. As a new approach to the use of natural populations for QTL analysis, genome-wide association (GWA) mapping has been widely applied for the mining of useful alleles in many species (Breseghello and Sorrells, 2006; Agrama et al., 2007; Kump et al., 2011; Morris et al., 2013; Zhang et al., 2014; Liu et al., 2015) due to its greater power to identify variants with weak effects compared with linkage studies (Risch and Merikangas, 1996). However, few studies have verified the loci and candidate genes underlying panicle length via association and linkage mapping. In this study, three populations, including a population of 254 recombinant inbred lines (RILs) derived from a cross between the cultivars Xiushui 79 (short panicle) and C-bao (long panicle), 540 rice accessions covering a wide geographical expanse (from 12.3°N to 47.4°N) and a single segment segregating F_2_ population, were used to identify QTLs and candidate genes associated with panicle length.

## Materials and methods

### Plant materials, cultivation, and measurements

Three mapping populations were used in the present study. A population consisting of 540 rice accessions from the geographical regions of East and Southeast Asia were used for association mapping, including 121 accessions from Vietnam (17°N–23°N), 400 from China and 11 from Japan (20°N–54°N) (Supplementary Table 1). A population consisting of 254 RILs derived from a cross between two japonica rice cultivars, Xiushui79 (female parent, panicle length 15.53 cm) and C-bao (male parent, panicle length 26.63 cm) were used for primary QTL analysis. A single segment segregating F_2_ population derived by crossing a single segment substitution line with Xiushui79 was used to confirm and finely map the major QTL controlling panicle length. All plants were grown in a paddy field at Jiangpu Experimental Station, Nanjing Agricultural University, Nanjing, China (31°56′N, 119°4′E). The seeds of natural and RILs populations were sown in the seedling nursery on 15 May in 2011 and 2012, and the seedlings were transplanted on 15 June in 2011 and 2012, planting one seedling per hill, with three replicates. The seeds of single segment segregating F_2_ population were sown in the seedling nursery on 12 May in 2014, and the seedlings were transplanted on 15 June in 2014. Each plot consisted of five rows with eight hills per row, and the hill spacing was 17 × 20 cm. Panicle length was measured as the length from the panicle neck to the panicle tip of the main panicle. The average of three replicates was subjected to association mapping and linkage analysis.

### Genotype analysis

DNA was extracted from fresh leaves of individuals of the 540 rice accessions, 254 RILs and single segment segregating F_2_ population using the method reported by Monna et al. (2002). A total of 262 SSR markers selected from the rice maps (Temnykh et al., 2000; McCouch et al., 2002) were used to genotype 540 rice accessions. PCR amplification was conducted in a 10-μL reaction mixture containing 1 μL of 20 ng μL^−1^ template DNA, 0.6 μL of 25 mmol L^−1^ MgCl_2_, 0.7 μL of 2 pmol μL^−1^ forward primers, 0.7 μL of 2 pmol μL^−1^ reverse primers, 0.2 μL of 2.5 mmol L^−1^ dNTP, 1 μL of 10 × PCR buffer, 0.1 μL of 5 U μL^−1^ rTaq DNA polymerase (TaKaRa, Japan) and 5.7 μL of ddH_2_O. DNA amplification was performed using a PTC-100 Peltier Thermal Cycler (MJ Research Inc., USA). The PCR program included denaturation at 95°C for 5 min, followed by 35 cycles of 95°C for 30 s, 55°C for 30 s, and 72°C for 30 s, and a final extension step at 72°C for 5 min. The PCR products were separated through electrophoresis on 8% non-denaturing polyacrylamide gels at a voltage of 180 V for approximately 100 min and then visualized via silver staining (Creste et al., 2001).

### Population structure and linkage disequilibrium

The population structure of 540 rice accessions was analyzed with STRUCTURE 2.2 (Falush et al., 2003). Twenty independent runs were performed for each k (from 2 to 10) using a burn-in length of 50,000, a run length of 100,000 and a model for the admixture and independent allele frequency. The mean log-likelihood value over 20 runs at each K value was used to identify the true number of populations (K), which was determined when the mean log-likelihood value reached the highest value for the model parameter K. The genetic distance was calculated from the 262 molecular markers using Nei's distance (Nei et al., 1983), and phylogenetic reconstruction was based on the Neighbour-joining method implemented in PowerMarker version 3.25 (Liu and Muse, 2005). A principal component analysis (PCA) was performed using the pcaMethods package of R.2.11.1 (Stacklies et al., 2007) to examine the population structure.The decay of LD (with distance in cM) between the SSR loci within the same chromosome was evaluated using 1000 permutations and calculated using TASSEL 2.1 software (Bradbury et al., 2007).

### Association mapping

The general linear model (GLM) and mixed linear model (MLM) in TASSEL 2.1 software were used for association mapping. The population structure (Q) was included as a covariate in the GLM to test for marker-trait associations and the matrices Q and K were used as covariates in the MLM analysis (Dang et al., 2014). The K matrix (kinship matrix) was obtained from the results of the relatedness analysis using SPAGeDi (Hardy and Vekemans, 2002). The allelic effects were estimated compared with the “null allele” (non-amplified alleles) for each locus (Flavio et al., 2006). The formula that was used to calculate the average positive (negative) allelic effects (AAE) within a locus was:

AAE=∑acnc

where *a*_*c*_ represents the phenotypic value of the cth allele with a positive (negative) effect, and *n*_*c*_ represents the number of alleles with positive (negative) effects within the locus.

### Genetic linkage map and QTL analysis

A total of 254 RILs derived from a cross between the cultivars Xiushui 79 and C-bao were used to conduct QTL analysis of panicle length using 91 SSR markers. The linkage map was constructed with MapMaker3.0/EXP version 3.0 (Lander et al., 1987). QTL analysis was performed using the inclusive composite interval mapping method in IciMapping, version 3.3 (http://www.isbreeding.net/), based on a stepwise linear regression model (Wang, 2009).

### Map-based cloning of *LP1*

Primers were designed around *LP1* on chromosome 9 of rice using indels identified between the indica cultivar 93-11 and the japonica cultivar Nipponbare using the software Primer Premier 5.0. Twelve of the 82 selected SSR primers in the preliminary mapping region showed polymorphisms between Xiushui79 and C-bao, which were available from the Rice Annotation Project Database (RAP-DB, http://rapdb.dna.affrc.go.jp/). These primers were used to genotype and identify the recombinants of the single-locus segregating F_2_ population, which included a total of 8650 individuals (Supplementary Table 2). The number of recombinants was identified based on a combination of genotype and phenotype data.

### Expression analysis of candidate genes

Total RNA was extracted from various tissues of Xiushui79 and NIL-*LP1* plants using the Ultrapure RNA Kit (CoWin Biotech Co., China). Genomic DNA contamination was removed by treatment with RNase-free DNase I. First-strand cDNA was synthesized using 6 μg of RNA and 4 μg of reverse transcriptase mix (PrimeScript RT Master Mix Perfect Real Time; TaKaRa Bio, Inc.) in a volume of 20 μL. Real-time quantitative RT-PCR was performed in a total volume of 20 μL containing 2 μL of template cDNA, 10 μL of qPCR SYBR Green Master Mix (Vazyme), 0.4 μL of forward and reverse gene-specific primers, 0.4 μL of ROX reference dye1, and 6.8 μL of ddH_2_O. Gene expression was normalized against 18S rRNA as an internal control. The amplification reaction was performed in a 96-well thermocycler (Roche Applied Science LightCycler 480) using the AceQ qPCR Kit (Vazyme). The cycling program consisted of 5 min at 95°C, followed by 40 cycles of amplification (95°C for 10 s and 60°C for 30 s). The primers are listed in Supplementary Table 2. The relative gene expression of the target gene was calculated using the following equation:

$$\begin{array}{l} Exp=2^{-\Delta Ct} \end{array}$$

where Δ*Ct* = *Ct*_*t*arg*etgene*_−*Ct*_18*SrRNA*_ (Livak and Schmittgen, 2001).

### Sequencing analysis of *LP1*

Gene-specific PCR primers were designed to amplify the full-length DNA sequence of *LP1* from Xiushui79 and NIL-*LP1* (Supplementary Table 2). The PCR product was gel purified and sequenced by GenScript Corporation Ltd., Nanjing, China. Multiple sequence alignments were performed using DNAMAN.

### Phylogenetic analysis of *LP1*

The amino acid sequences of *LP1* homologs were obtained from NCBI (http://www.ncbi.nlm.nih.gov/). Then, the protein sequences were aligned using CLUSTAL W in MEGA5.0, and the result was used to construct a bootstrap N-J phylogenetic tree. A total of 1000 replicates were conducted to determine the statistical support for each node.

### Sequence polymorphisms of *LP1* in rice accessions

A total of 103 rice accessions showing abundant diversity in panicle length were selected to sequence a 5.4-kb genomic DNA fragment of *LP1*. Association analysis of the SNPs and indel markers in the 5.4-kb region of the 103 accessions with panicle length was conducted.

## Results

### Phenotypic variation of panicle length in natural and ril populations

**Figure 1:**
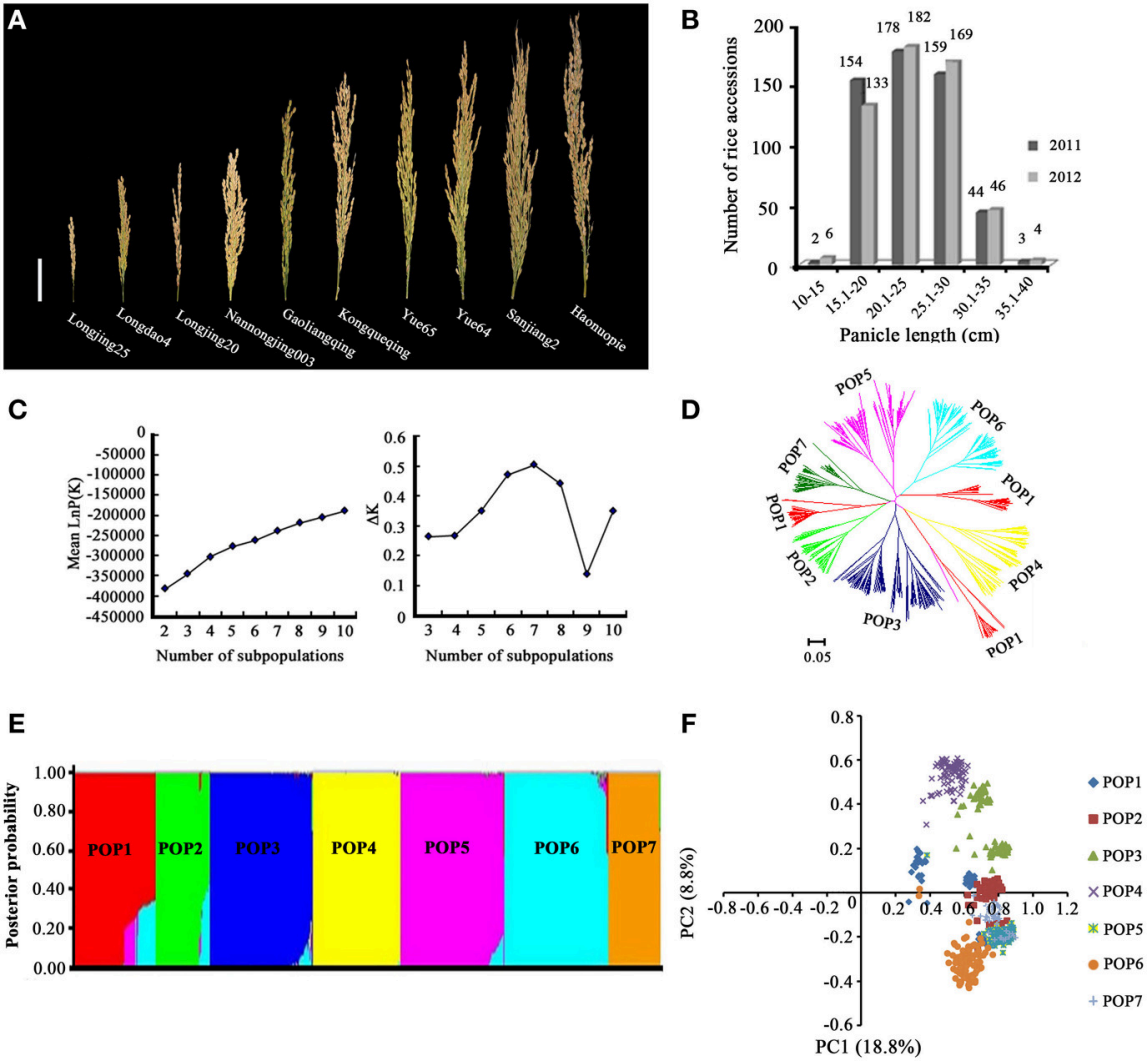
**Population structure analysis of 540 rice accessions. (A)** Panicle length of 10 rice accessions selected from 540 accessions to represent the diversity of panicle length in the population. Bar = 5 cm. **(B)** Frequency distribution of the panicle length of the 540 accessions in 2011 and 2012. **(C)** Changes in mean LnP(K) and ΔK. **(D)** Neighbor-joining tree for the 540 accessions generated using Nei's genetic distance. **(E)** Posterior probability of the 540 accessions belonged to seven subpopulations. **(F)** Principal components analysis (PCoA) for 540 accessions and reference cultivars genotyped with 262 molecular markers.

Panicle length differed significantly among the 540 accessions in both 2011 and 2012, with CVs of 19.45 and 19.56%, respectively (Supplementary Table 3). A high broad-sense heritability value was observed in both years. We selected ten varieties to represent the diversity of panicle length among the 540 rice accessions, including the shortest-panicle accession, Longjing25 (11.91 cm), and the longest-panicle accession, Haonuopie (39.98 cm) (Figure 1A). The frequency distribution of panicle length in the 540 accessions recorded in 2011 and 2012 is presented in Figure 1B. The means of panicle length over 254 RILs were 21.1 and 20.9 cm in 2011 and 2012, with CVs of 15.25% and 15.57%, respectively. RIL242 had the smallest panicle length (14.0 cm in 2011 and 14.9 cm in 2012), and RIL31 had the largest panicle length (29.0 cm in 2011 and 27.1 cm in 2012).

### GWA mapping and linkage analysis for panicle length

STRUCTURE analysis using 262 simple sequence repeat (SSR) markers revealed that the log-likelihood increased as the model parameter K increased; thus, the statistic ΔK was used to determine a suitable value for K. The ΔK value was much higher for the model parameter *K* = 7 than for other values of K (Figure 1C), indicating that the population used in this study was a mixed population consisting of seven subpopulations (Figure 1E). A neighbor-joining tree among the 540 rice accessions was constructed based on Nei's (1983) genetic distance (Nei et al., 1983) (Figure 1D), and the results were consistent with the results of the structure analysis. The results of the PCA were essentially consistent with those from STRUCTURE. The two major PCs from the PCA explained 27.6% (18.8 and 8.8%) of the total variance (Figure 1F).

Regression analysis between the D′ value and the genetic distance of syntenic marker pairs revealed that the genomes of the seven subpopulations fit the equation*y* = *b*ln *x*+*c* (Supplementary Figure 1). The minimum distances of LD decay were 60.2, 13.0, 85.4, 70.8, 29.8, 72.9, and 61.8 cM for the seven subpopulations.

Total fifteen SSR markers associated with panicle length were detected by both GLM and MLM analysis. GLM analysis of marker-trait associations revealed 10 SSR markers associated with panicle length (*p* < 0.05), located on chromosomes 2, 3, 5, 6, 8, 9, and 10, in both years. PVE ranged from 10.32% to 32.63%. RM5475 on Chr3 explained the maximum percentage of phenotypic variation: 28.38% in 2011 and 32.63% in 2012 (Table 1). RM3600 on Chr9, residing on site of 1.46 Mb, exhibited the second highest PVE values, of 22.19% in 2011 and 22.17% in 2012 (Table 1). MLM analysis revealed seven SSR markers associated with panicle length (*p* < 0.05), located on chromosomes 2, 6, 7, 9, and 11, in both years. PVE ranged from 2.09 to 25.82%. RM295 on Chr7 exhibited the highest PVE: 24.52% in 2011 and 25.82% in 2012. RM3600 on Chr9 explained 16.97% PVE in 2011 and 19.69% PVE in 2012. Two SSR markers, RM3600 on Chr9 and RM3688 on Chr2, were both detected by GLM and MLM methods (Table 1). In this study, the alleles with positive effects were considered elite alleles for panicle length. The ten best elite alleles and their typical carrier materials are described in Supplementary Table 4. The allele RM3600-130 bp exhibited the largest phenotypic effect (8.69 cm) on panicle length, and the typical carrier accession was Yue 33 (Supplementary Table 4).

**Table 1:** **Marker-trait associations with *P*-values of less than 0.05 by GLM and MLM analysis, the proportion of phenotypic variance explained (PVE) and the average positive (negative) allele effects (AAE) in 2011 and 2012**.

**No**.	**Marker names**	**Chr**.	**Start position (bp)**	**2011**		**2012**		**AAE**		**Analysis methods**
				***P*-value**	**PVE (%)**	***P*-value**	**PVE (%)**	**Positive**	**Negative**	
1	RM3688	2	22,400,777	0.0500	19.28	0.0251	22.59	+2.65	−1.88	GLM
				0.0384	2.57	0.0480	2.09			MLM
2	RM213	2	34,658,315	0.0274	17.67	0.0028	27.27	+5.64	−2.06	GLM
3	RM266	2	35,431,738	0.0347	2.95	0.0377	2.77	+3.26	−1.38	MLM
4	RM535	2	35,784,312	0.0271	12.21	0.0454	10.32	+4.00	−3.08	GLM
5	RM5475	3	30,577,227	0.0372	28.38	0.0157	32.63	+2.37	−3.83	GLM
6	RM480	5	27,376,099	0.0323	11.50	0.0194	13.55	+3.44	−2.35	GLM
7	RM314	6	4,845,258	0.0222	18.60	0.0229	18.46	+2.42	−1.65	GLM
8	RM276	6	6,231,149	0.0303	21.70	0.0104	26.57	+3.60	−3.04	GLM
9	RM5389	6	8,276,087	0.0324	10.33	0.0067	9.34	+2.98	−1.24	MLM
10	RM6811	6	28,600,179	0.0270	4.51	0.0398	3.96	+3.66	−1.15	MLM
11	RM295	7	414,668	0.0482	24.51	0.0123	25.82	+2.88	−1.34	MLM
12	RM264	8	27,926,632	0.0424	10.40	0.0223	13.00	+2.29	−3.11	GLM
13	RM3600	9	17,054,142	0.0109	22.19	0.0110	22.17	+6.16	−2.25	GLM
				0.0086	16.97	0.0042	19.69			MLM
14	RM184	10	16,430,316	0.0161	14.30	0.0198	13.48	+1.62	−2.98	GLM
15	RM286	11	384,837	0.0335	10.33	0.0380	8.05	+1.98	−2.04	MLM

*GLM, general linear model; MLM, mixed linear model*.

A total of four additive QTLs were detected for panicle length on chromosomes 4, 6, and 9 in the RILs, including SSR marker RM551 (0 cM) on Chr4 and regions between RM3288 and RM349 (7.0 cM) on Chr4, between RM5314 and RM454 (0.9 cM) on Chr6, and between RM5652 and RM410 (7.0 cM) on Chr9 (Table 2). Each QTL explained 1.2–34.9% of the observed phenotypic variation (Table 2), with *LP1* explaining 34.9% of the phenotypic variation, representing the highest value among the four QTLs.

**Table 2:** **Estimated position, flanking markers, additive effects and percentage of phenotypic variation explained (R^2^) for the QTLs for panicle length in RILs population**.

**QTL**	**Marker interval**	**Distance (cM)**	**Position (bp)**	**LOD**	***A* effect**	***P*-value**	***R^2^* (%)**
*PL4-1*	RM551	0.0	(4)168,711	12.8	−0.56	<1 × 10^−4^	5.6
*PL4-2*	RM3288-RM349	7.0	(4)27,516,234–32,718,571	9.1	−0.59	<1 × 10^−4^	3.6
*PL6*	RM5314-RM454	0.9	(6)23,381,619-24,460,234	22.1	−0.81	<1 × 10^−4^	1.2
*LP1*	RM5652-RM410	7.0	(9)14,731,280–17,589,271	115.8	−1.35	<1 × 10^−4^	34.9

GWA mapping revealed that the SSR marker RM3600 was associated with PL and was located in the region between RM5652 and RM410 of the QTL *LP1* detected through linkage analysis in the RILs (Tables 1, 2). The locus explained more than 20% of the variation of panicle length in both the linkage and GWA mapping analyses, indicating that *LP1* is a major QTL controlling panicle length in rice.

### Phenotypic performance of panicle length in a fine-mapping population

**Figure 2:**
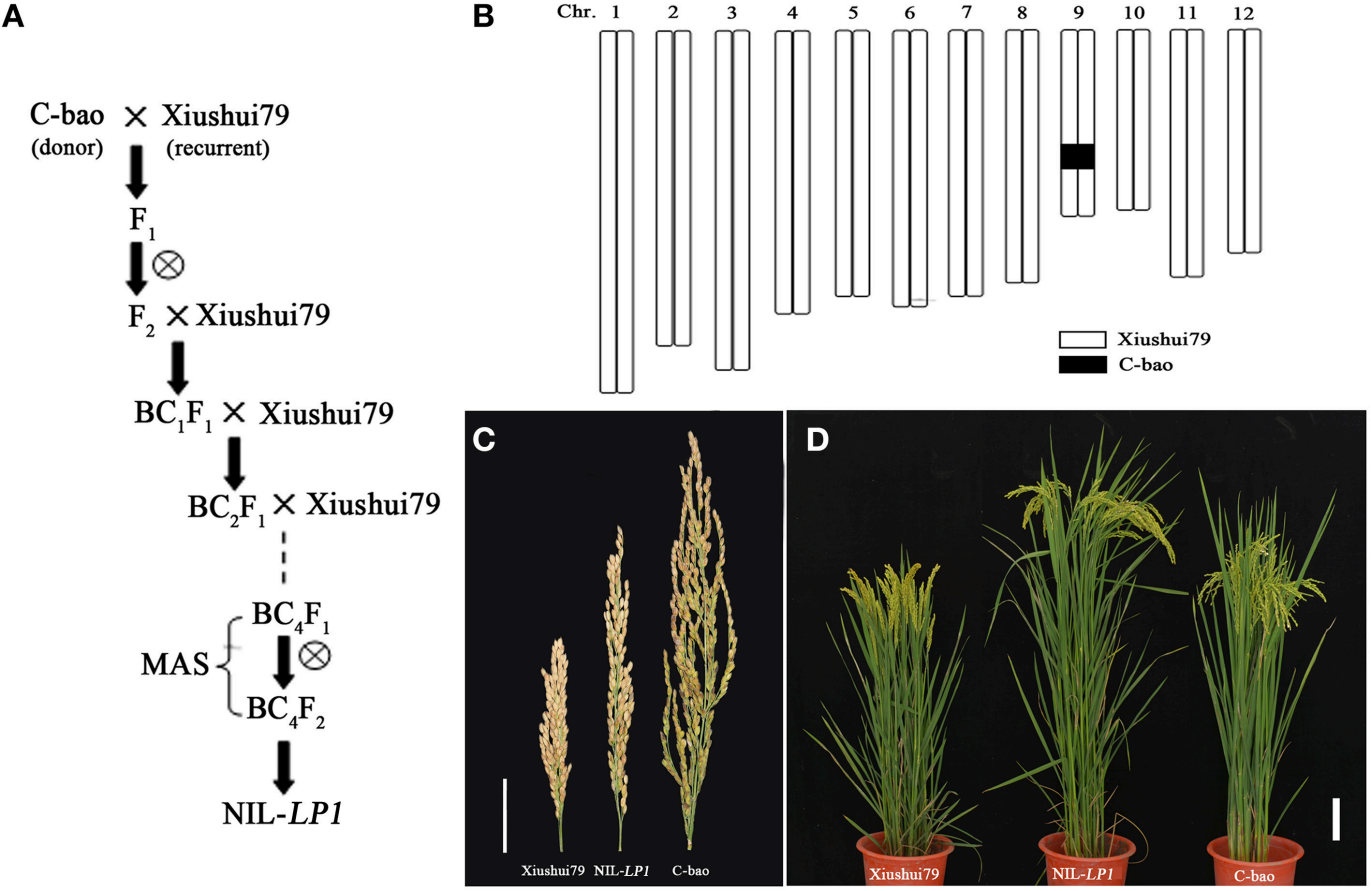
**Genotypic and phenotypic performance of the parents. (A)** Workflow of the construction of one NIL plant (BC_4_F_2_), NIL-*LP1*. **(B)** Graphical genotype of NIL-*LP1*. The black bar indicates the fragment from C-bao, and the remainder was derived from Xiushui79. **(C)** Panicle morphology of Xiushui79, NIL-*LP1* and C-bao. Bar = 5 cm. **(D)** Xiushui79, NIL-*LP1*, and C-bao plants. Bar = 10 cm.

A NIL plant containing C-bao allele at the *LP1* in genetic background of Xiushui79 isolated from BC_4_F_2_ generation (Figures 2A,B,D) was employed to develop a single segment segregating F_2_ population to confirm and finely map the major QTL *LP1*. The panicle length of NIL-*LP1* homozygous plant was significantly longer than that of Xiushui79 (i.e., 21.90 ±2.70 cm vs. 15.83 ± 1.90 cm) in 2014, which was consistent with the measurements obtained in 2013 (Table 3, Figure 2C). By contrast, the F_1_progeny of NIL-*LP1*/Xiushui79 exhibited a panicle length that was intermediate to those of the two parents (19.26 ± 1.21 cm) in 2014, indicating a semi-dominant pattern of expression of the allele for long panicle length. Among the 1980 plants randomly selected from the single-segment segregating F_2_population, the phenotypic separation ratio was fitted to 1:2:1 [460 Xiushui79 homozygote type: 1017 heterozygote type: 503 NIL-*LP1* homozygote type, $\chi_{\left(1:2:1\right)}^{2}$ = 3.22 < $\chi_{\left(0.05,2\right)}^{2}$ = 5.99], suggesting that panicle length is controlled by a single-gene locus (Supplementary Figure 2B). The other agronomic traits of NIL-*LP1* were evaluated under field conditions. Compared with Xiushui79, NIL-*LP1* exhibited an increase in yield of 13.73% resulting from increases of 41.02% in panicle length, 10.90% in the number of filled grains per panicle, 2.69% in grain width, and 1.05% in the thousand filled-grain weight (Table 3). No significant differences were observed between Xiushui79 and NIL-*LP1* regarding days to heading, the panicle number per plant, seed setting rates, grain length, and grain thickness (Table 3). Therefore, the increase in grain yield observed in NIL-*LP1* was mainly due to the improvement of panicle length.

**Table 3:** **Comparison of panicle length and other agronomic traits between Xiushui79 and NIL-*LP1***.

**Traits**	**Xiushui79**	**NIL-*LP1***	***P*-value**
PL (cm)	15.83 ± 1.90	21.90 ± 2.70	5.18 × 10^−6^
HD(d)	103.0 ± 0.3	104 ± 0.3	0.37
PH (cm)	80.33 ± 3.45	102.33 ± 4.20	3.09 × 10^−4^
PP	15.3 ± 1.2	17.0 ± 1.6	0.28
NSP	110.0 ± 15.5	126.7 ± 23.8	1.57 × 10^−2^
NFGP	107.3 ± 12.6	119.0 ± 17.5	3.22 × 10^−2^
SS (%)	97.55 ± 0.21	94.01 ± 0.33	0.11
TGW (g)	28.413 ± 1.041	28.712 ± 1.261	1.53 × 10^−2^
GL (mm)	7.51 ± 0.11	7.52 ± 0.13	0.63
GW (mm)	3.35 ± 0.23	3.44 ± 0.21	3.53 × 10^−2^
GT (mm)	2.39 ± 0.15	2.40 ± 0.19	0.84
YPP (kg)	2.04 ± 0.51	2.32 ± 0.66	4.26 × 10^−3^

*All trait data are presented as the mean ± SD. Each P-value for each trait was obtained from a t-test between Xiushui79 and NIL-LP1. PL, panicle length; HD, days to heading (summer 2015, Nanjing, Jiangsu, China); PH, plant height; PP, panicle number per plant; NSP, number of spikelets per panicle; NFGP, number of filled grains per panicle; SS, seed setting rate; TGW, thousand filled-grain weight; GL, grain length; GW, grain width; GT, grain thickness; YPP, yield per plot*.

### Map-based cloning of *LP1*

**Figure 3:**
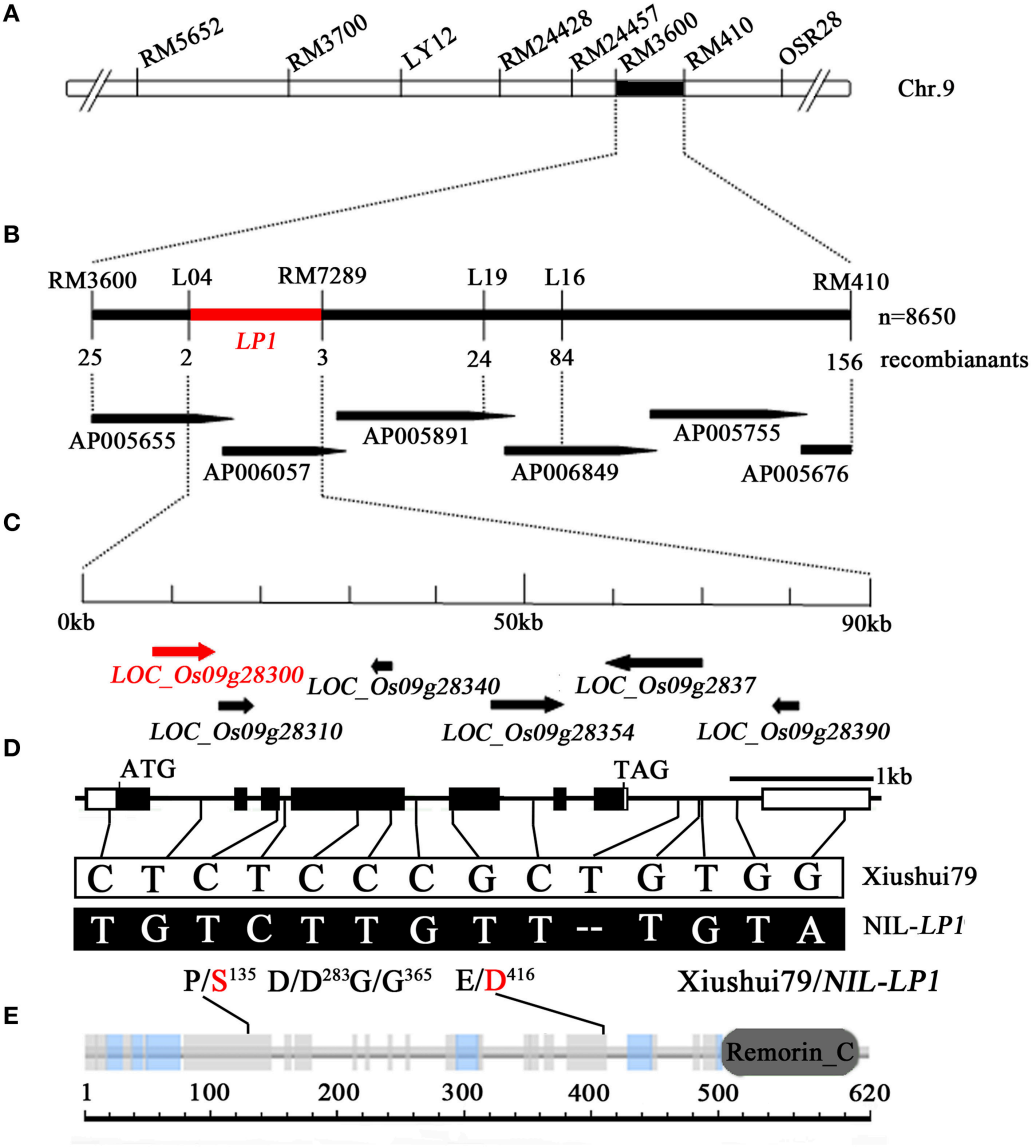
**Map-based cloning of *LP1*. (A)** Primary mapping of *LP1*. *LP1* was mapped between the SSR markers RM3600 and RM410. **(B)** High-resolution mapping of *LP1*. *LP1* was delimited to a 90-kb region between the markers L04 and RM7289 in the BAC clones AP005655 and AP006057, using a total of 8650 plants from the NIL-*LP1*/Xiushui79 F_2_ population. **(C)** Predicted ORFs based on the Nipponbare genome sequence in the Rice Genome Annotation Project database (http://rice.plantbiology.msu.edu/index.shtml). The horizontal arrows indicate the six predicted ORFs. **(D**) Gene structure of *LOC_Os09g28300*. The empty boxes refer to the 5′ and 3′ UTRs; the black boxes, to exons; and the lines between the boxes, to introns. The fourteen SNPs in *LP1* are indicated by solid lines. **(E)** The Remorin_C domain predicted in the protein encoded by *LOC_Os09g28300*. The solid lines indicate the positions of two amino acid transitions.

Further QTL analysis was conducted in 176 individuals from the NIL-*LP1*/Xiushui79 F_2_ population. The *LP1* QTL was delimited to a 1.60-cM region between RM3600 and RM410 with an LOD peak of 101.67 (Supplementary Figure 2A, Figure 3A). A total of 8650 F_2_ plants were subjected to marker analysis by scanning RM3600 and RM410 (confidence interval markers of *LP1* in preliminary mapping). Analysis of RM3600 identified 25 recombination events between the marker and *LP1* on one side, whereas an analysis of RM410 detected 156 recombination events between the marker and *LP1* on the other side. The insertion/deletion (indel) marker L04 revealed two recombinants, whereas RM24496, RM24489, and RM7289 revealed 84, 24, and 3 recombinants, respectively, on the other side (Figure 3B). Therefore, *LP1* was mapped to a 90-kb DNA region between L04 and RM7289 (Figure 3C). This region contains six predicted ORFs according to the Nipponbare genome sequence (annotated by Rice Genome Annotation Project, http://rice.plantbiology.msu.edu/index.shtml), including *LOC_Os09g28300, LOC_Os09g28310, LOC_Os09g28340, LOC_Os09g28354, LOC_Os09g28370*, and *LOC_Os09g28390* (Figure 3C, Supplementary Table 5).

**Figure 4:**
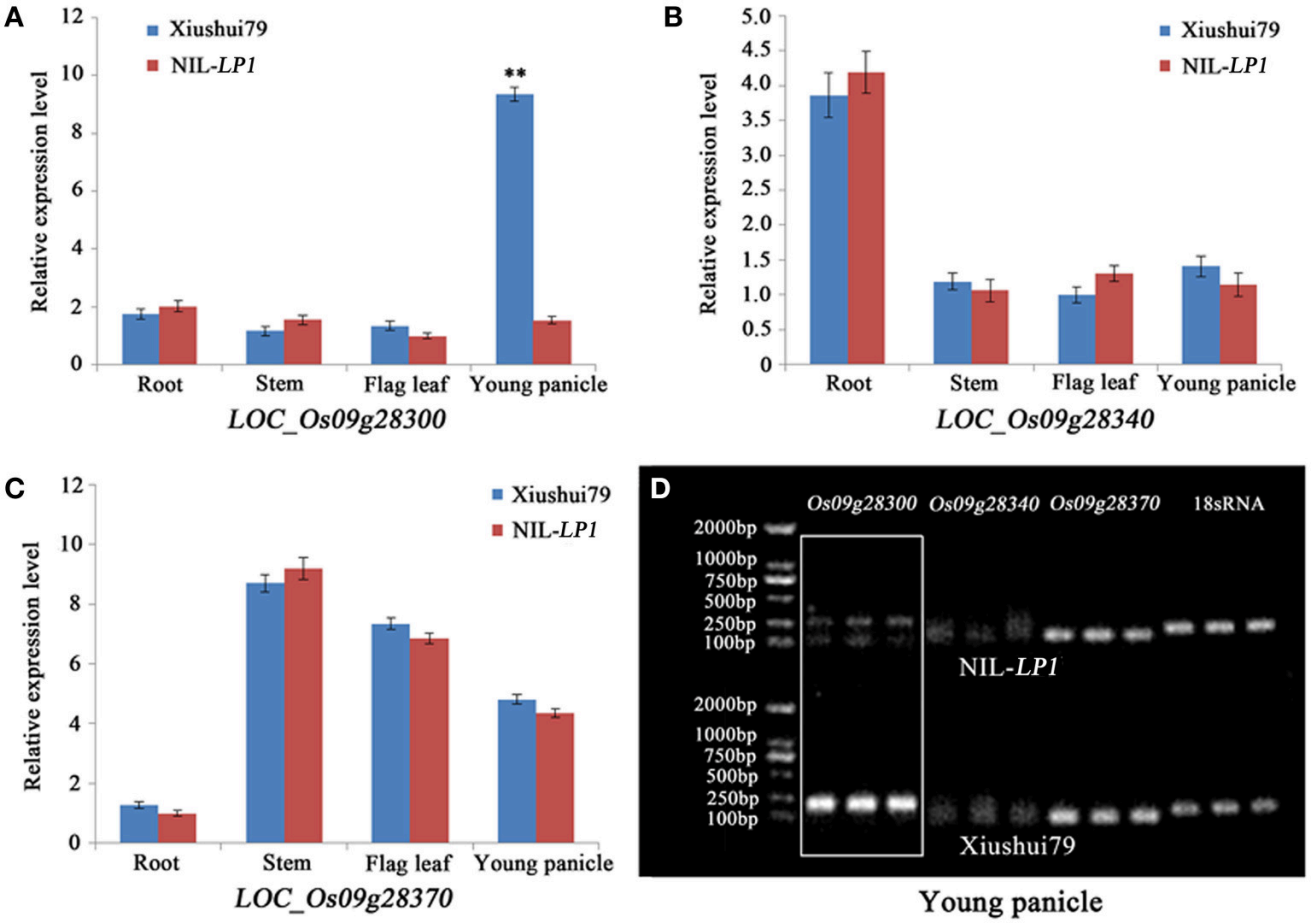
**Analysis of the expression of the three expressed candidate genes via real-time quantitative RT-PCR. (A)** Relative expression of *LOC_Os09g28300* in the roots, stems, flag leaves and young panicles. **(B)** Relative expression of *LOC_Os09g28340* in the roots, stems, flag leaves and young panicles. **(C)** Relative expression of *LOC_Os09g28370* in the roots, stems, flag leaves and young panicles. **(D)** Real-time quantitative RT-PCR results for the three candidate genes in the young panicles. The 18S rRNA gene was used as a control. ^**^ indicates significance at the α = 0.01 probability level.

Real-time quantitative RT-PCR was performed to further analyze the candidate genes by detecting differences in expression between the parents. Only *LOC_Os09g28300, LOC_Os09g28340*, and *LOC_Os09g28370* were expressed. Among these three genes, *LOC_Os09g28340* and *LOC_Os09g28370* exhibited no differential expression in the roots, stems, flag leaves and young panicles between Xiushui79 and NIL-*LP1* (Figures 4B,C,D). The expression of *LOC_Os09g28300* was significantly up-regulated (6.08-fold) in the young panicles of Xiushui79 compared with NIL-*LP1* (Figures 4A,D).

Sequence comparison of *LOC_Os09g28300* between Xiushui79 and NIL-*LP1* revealed 14 SNPs, four of which were located in exons and 10 in introns. SNP1, a single nucleotide transition from C (Xiushui79) to T (NIL-*LP1*), was identified in the third exon of *LOC_Os09g28300*, and SNP2, a single nucleotide transversion from G (Xiushui79) to T (NIL-*LP1*), was found in the fifth exon (Figure 3D). These two polymorphisms cause amino acid residue changes from proline to serine (P/S^135^) and glutamic acid to aspartic acid (E/D^416^), respectively (Figure 3D). SNP3 (C → T, in the 4th exon, D/D^283^) and SNP4 (C → T, in the 4th exon, G/G^365^) do not cause amino acid substitutions (Figure 3D).

### *LP1* encodes a remorin_C-containing protein

**Figure 5:**
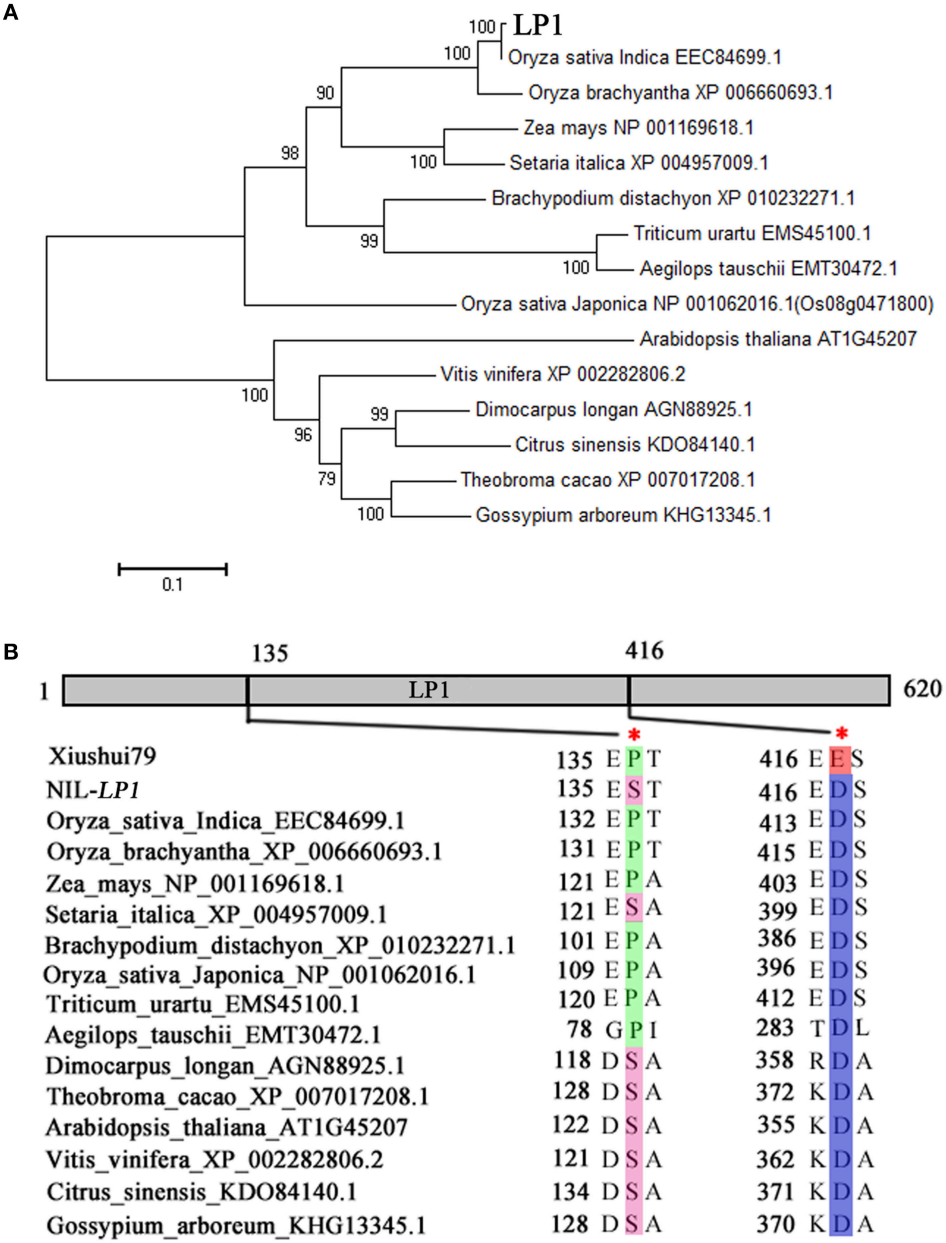
**Phylogenetic analysis of the *LP1* protein. (A)** Phylogenetic tree of the *LP1*-like proteins in higher plants. The amino acid sequences of 15 *LP1* homologs were obtained from NCBI (http://www.ncbi.nlm.nih.gov/) and used to construct a bootstrap N-J phylogenetic tree. A total of 1000 replicates were performed to determine the statistical support for each node. *LP1* is denoted in bold. **(B)** Diagram indicating the two amino acid substitutions in *LP1* at positions conserved across LP1-like proteins.

*LOC_Os09g28300* encodes a C-terminal region domain-containing protein (Remorin_C). These proteins are plant-specific plasma membrane-associated proteins of unknown function (Figure 3E). Phylogenetic analysis revealed the presence of Remorin_C in many species, including rice, maize, millet, *Bachypodium distachyon*, wheat, and *Arabidopsis* (Figure 5A). Among the identified homologs, the maize homolog exhibits the highest homology with rice, sharing 67.4% amino acid identity with LP1 (Supplementary Figure 3). In addition, LP1 shares 54.2% amino acid identity with Os08g36760, which is encoded on chromosome 8 in rice. The two polymorphisms that lead to amino acid substitutions, P/S^135^ and E/D^416^, are located at conserved positions among the homologs (Figure 5B). At the E/D^416^ locus, only Xiushui79 exhibits glutamic acid, whereas NIL-*LP1* and the other species exhibit aspartic acid. These differences in conserved amino acids may affect LP1 function.

### Sequence polymorphisms of *LP1* in rice accessions

A total of 103 rice accessions with abundant diversity in panicle length selected from the 540 accessions were used to sequence a 5.4-kb genomic DNA fragment encompassing the mutation sites leading to amino acid substitutions in *LP1*. A total of 22 SNPs were identified (Supplementary Table 6). Based on the sequencing results, seven accessions carrying the same SNP1 as C-bao were identified, whereas polymorphisms in SNP2, SNP3, and SNP4 were widely distributed in both japonica and indica accessions (Supplementary Table 6). Association analysis of the SNPs and indel markers in the 5.4-kb region with panicle length in the two parents and 103 rice accessions revealed that SNP1 was significantly associated with panicle length at α = 0.01 probability level, whereas other polymorphic sites made no significant contributions to panicle length (Supplementary Table 7). These results indicate that SNP1 identical with Xiushui 79 is a major functional mutation for short panicles.

## Discussion

Panicle length is one of the most important traits for rice yields and ideal plant breeding. Previous studies have demonstrated that panicle length is a typical quantitative trait controlled by multiple genes and may be significantly influenced by environmental conditions (Yao et al., 2015). Therefore, the number and effect of QTLs for panicle length detected via linkage mapping may differ among segregating populations, such as F_2_ populations, RILs or backcrossed inbred lines (Huang and Han, 2014), and these differences may underlie the identification of approximately 253 QTLs for panicle length distributed on 12 chromosomes (Xiao et al., 1998; Hittalmani et al., 2002, 2003; Xing et al., 2002; Kobayashi et al., 2003; Thomson et al., 2003; Ashikari et al., 2005; Lee et al., 2005; Mei et al., 2005; Cho et al., 2007; Liu et al., 2011; Marathi et al., 2012; Yao et al., 2015; Zhang et al., 2015). GWA mapping, a new method that fully exploits ancient recombination events to identify the genetic loci underlying traits, is becoming a powerful tool for detecting natural variation underlying complex traits in crops (Rafalski, 2010). The results obtained using these two types of mapping methods may enable mutual authentication. Consequently, both association mapping using a natural population and linkage mapping using RILs were conducted in this study. The QTL *LP1* was detected through both association mapping and linkage analysis and was identified as a major QTL associated with high phenotypic variation for panicle length.

A natural population of 540 rice accessions with abundant phenotypic and genotypic diversity was employed to perform a succession study of panicle length in rice. Population structure and linkage disequilibrium are the basis for association analysis (Pritchard and Rosenberg, 1999; Flint-Garcia et al., 2003). Determining population structure can prevent false-positive results regarding associations between phenotypes and genotypes in association mapping due to the linkage disequilibrium in natural populations (Pritchard and Rosenberg, 1999). The seven subpopulations (POP1–POP7) grouped by structure were essentially consistent with geographic regions. For example, the accessions from Vietnam were basically classified as POP6, and the accessions from northeastern China were mostly classified as POP1. The LD decay distances among the seven subpopulations identified in this study ranged from 13 to 80 cM, which was longer than has been observed in previous studies (Agrama et al., 2007; Jin et al., 2010). These results indicate that the 540 rice accessions underwent artificial hybridization and selection.

By comparing the mapping results of population-based association mapping with those of the linkage mapping of the families, 10 marker-panicle length associations were detected in both 2011 and 2012, whereas only four related QTLs were identified, indicating that GWA mapping has greater power than linkage analyses to identify variants with weak effects. *LP1* was detected via the two approaches due to the strong effect of this QTL on panicle length in rice. Therefore, *LP1* was selected as a major QTL for further studies, including map-based cloning and candidate gene identification.

NIL development is a useful method for confirming and evaluating the genetic effects of a QTL and provides useful materials for population development during fine mapping of QTLs (Ding et al., 2011). This method has been successfully applied for fine mapping of QTLs in most crop species (Benson et al., 2015; Jang et al., 2015; Wang et al., 2015; Zheng et al., 2015). In the present study, we developed NILs in a backcrossing program using two parents, Xiushui79 (the receipt parent) and C-bao (the donor parent). The NILs exhibited an additive effect similar to that observed in the QTL analysis. Introgression of the *LP1* allele into the Xiushui79 background produced the longest panicles among the examined allele combinations, which will be valuable for breeding applications. NIL-*LP1* plants will serve as an important parent for the generation of a single-locus segregating F_2_ population for fine mapping.

In the present study, the chromosome segment containing *LP1* was delimited to a 90-kb region for the first time. Within the fine-mapping region, we identified six annotated genes: *LOC_Os09g28300, LOC_Os09g28310, LOC_Os09g28340, LOC_Os09g28354, LOC_Os09g28370*, and *LOC_Os09g28390*. Among the six candidate genes, *LOC_Os09g28300* was validated as the gene locus controlling panicle length. *LP1* is predicted to be a major regulator of panicle length in rice. Furthermore, real-time quantitative RT-PCR analysis revealed significant differences in the expression of *LOC_Os09g28300* in young panicles between Xiushui79 and NIL-*LP1*. The expression of *LOC_Os09g28300* was significantly higher in Xiushui79 than in NIL-*LP1*, indicating that the *LP1* allele of Xiushui79 suppresses panicle development in rice. Map-based cloning revealed that *LP1* encodes a Remorin_C-containing protein that may be closely related to plant development. The two SNP polymorphisms resulting in amino acid substitutions likely affect the function of the LP1 protein.

Sequence polymorphism analysis of *LP1* indicated that SNP1 is a functional mutation leading to short panicles. Most varieties with short panicles exhibit the same amino acid as Xiushui79 at the P/S^135^ locus, indicating that a change from proline to serine may relieve the suppression of short panicles. Consequently, the C-bao allele at *LP1* locus can be used to improve varieties with short panicles. As a major QTL, the NIL-*LP1* allele has beneficial effects on panicle length, the number of filled grains per panicle, the thousand filled-grain weight and grain width, and *LP1* should be selected in rice breeding.

**Figure 6:**
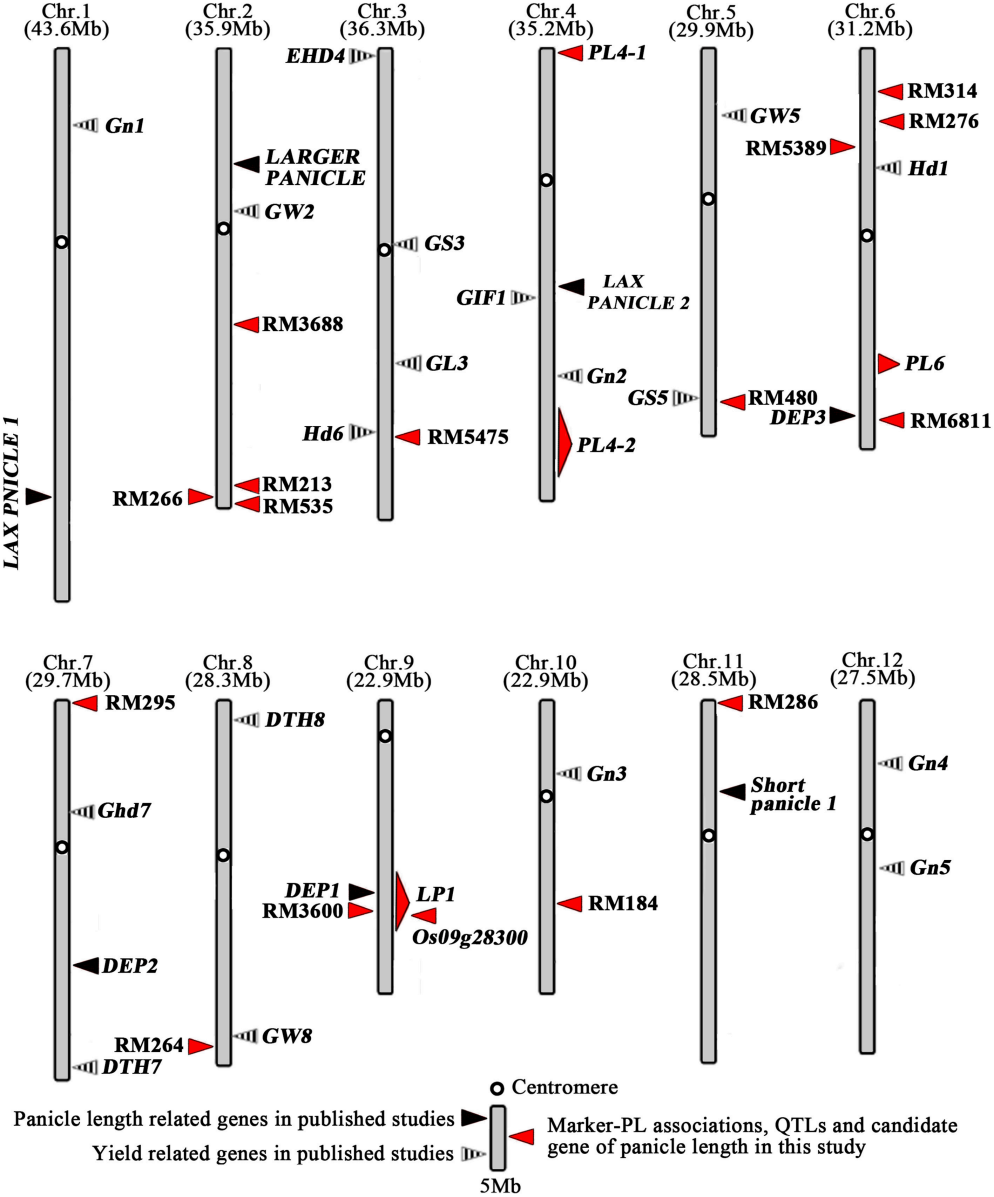
**Distribution of marker-PL and QTLs or genes for panicle length and panicle length related on rice chromosome**.

Compared the QTLs detected in this study for panicle length with other studies, we found that *DEP1* (Huang et al., 2009), mapped to the interval between the marker RM3700 and RM7424 on Chr9, was within the region of QTL *LP1* detected in this study (Figure 6). However, the results of fine mapping show that the physical distance between *LP1* and *DEP1* is about 770 kb, which indicated that *LP1* was a novel gene within the region of QTL *LP1* (Figures 3, 6).Although other genes associated with the panicle phenotype have been cloned, the present study is the first to observe the involvement of *LP1*. The results of this study provide an opportunity for further functional analysis of *LP1* to elucidate the mechanism of rice panicle improvement. *LP1* can be used to create new varieties to improve rice yields. Compared the marker-PL associations with previous studies, we found 11 of the 15 SSR markers detected in this study were novel, and the other four SSR markers were located near to the chromosome regions harboring panicle and yield related QTLs or genes which have been reported. Among them, RM5472 on chromosome3 was near to the chromosome regions covering *Hd6* of heading-related gene shown in Figure 6 (Takahashi et al., 2001). Marker RM480 on Chr4 associated with panicle length was near to chromosome region harboring the gene *GS5* (Li Y. et al., 2011) (Figure 6). The marker RM6811 was near to the region of *DEP3* mapped on chromosome 6 (Qiao et al., 2011) (Figure 6). RM264 on chromosome 8 was near to the region containing *GW8* (Wang et al., 2012) (Figure 6). Utilization of all of the elite alleles that were detected in this study may improve the panicle length of parents of F_1_ hybrid rice via pyramiding breeding.

## Author contributions

DH designed the research; EL, YL, GW, SZ, and TT carried out the field experiment; EL, YL, LL, YFL, ZD, DS, HW and IZ carried out the molecular experiment; EL analyzed data; and EL wrote the manuscript; DH revised the manuscript.

## Funding

This work was supported by the National High-tech R&D Program of China (863 Program) (Grant number: 2010AA101301) and Research Fund for the Doctoral Program of Higher Education of China (Grant numbers B0201100690, B0201300662).

### Conflict of interest statement

The authors declare that the research was conducted in the absence of any commercial or financial relationships that could be construed as a potential conflict of interest. The reviewer DG and handling Editor declared their shared affiliation, and the handling Editor states that the process nevertheless met the standards of a fair and objective review.

## Supplementary material

Supplementary Figure 1 **(A–G)** Relationship between the D' value and genetic distance of syntenic marker pairs in subpopulations. **(H)** Gene diversity and PIC values of the seven subpopulations.

Supplementary Figure 2 **Quantitative trait loci analysis using NIL-*LP1*/Xiushui79 F_2_ individuals. (A)** Molecular linkage map of rice chromosome 9 showing the location of *LP1*. **(B)** Distribution of panicle length in the segregating population for the *LP1* locus.

Supplementary Figure 3 **Alignment of *LP1* with the amino acid sequences of LP1-like proteins from other species**. Asterisks indicate identical amino acids.

Supplementary Table 1 **Panicle length of the 540 rice germplasm accessions, their geographical origin and their membership probabilities corresponding to each subpopulation**.

Supplementary Table 2 **Primer sequences designed in this study**.

Supplementary Table 3 **Descriptive statistics for panicle length (cm) in 540 rice accessions in 2011 and 2012**.

Supplementary Table 4 **Phenotypic effects of top 10 elite alleles at loci significantly associated with panicle length and their carrier variety**.

Supplementary Table 5 **Six annotated genes and their putative functions in a 90-kb region of the rice genome**.

Supplementary Table 6 **The SNPs detected at the 5.4-kb region of *LP1* among two parents and 103 rice accessions**.

Supplementary Table 7 **Association analysis of the polymorphic markers in *LP1* with panicle length**.
