# Supplementary figures and images for: Identification of a Candidate Gene for Panicle Length in Rice (Oryza sativa L.) Via Association and Linkage Analysis
Source: Front Plant Sci. 2016 May 3;7:596. doi: 10.3389/fpls.2016.00596 (PMC4853638; doi:10.3389/fpls.2016.00596)

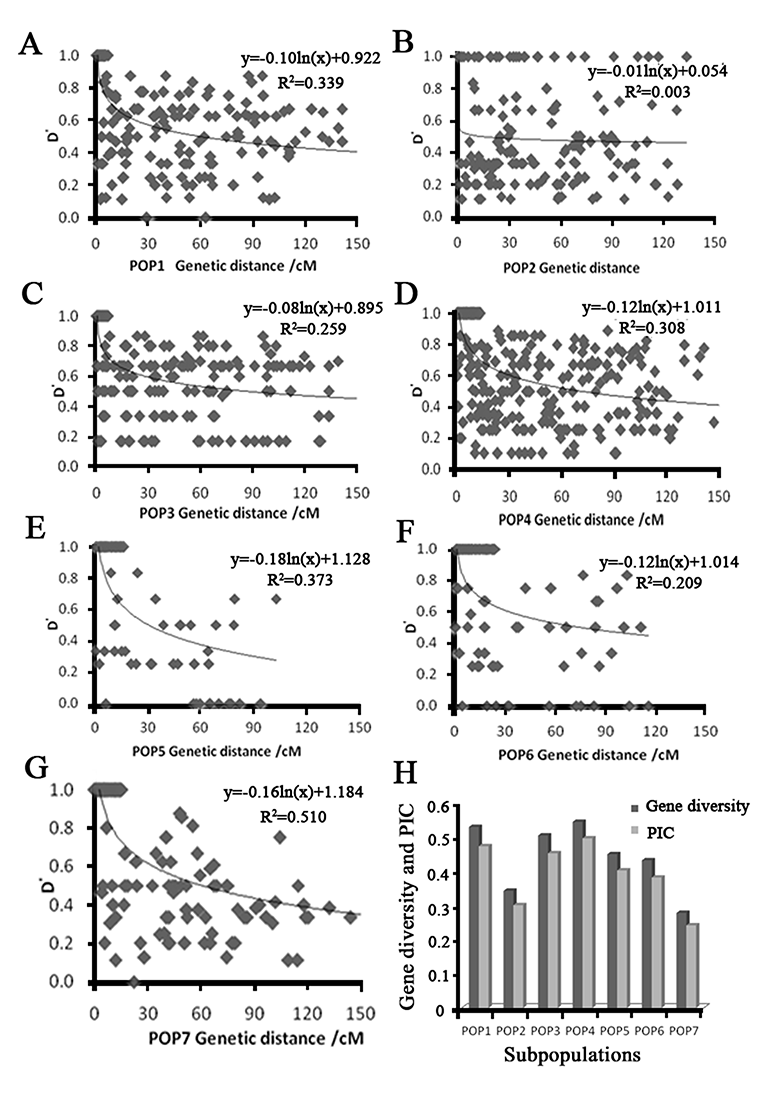

Supplement: Supplementary Figure 1 — (A–G) Relationship between the D' value and genetic distance of syntenic marker pairs in subpopulations. (H) Gene diversity and PIC values of the seven subpopulations. [file Image1.TIF]

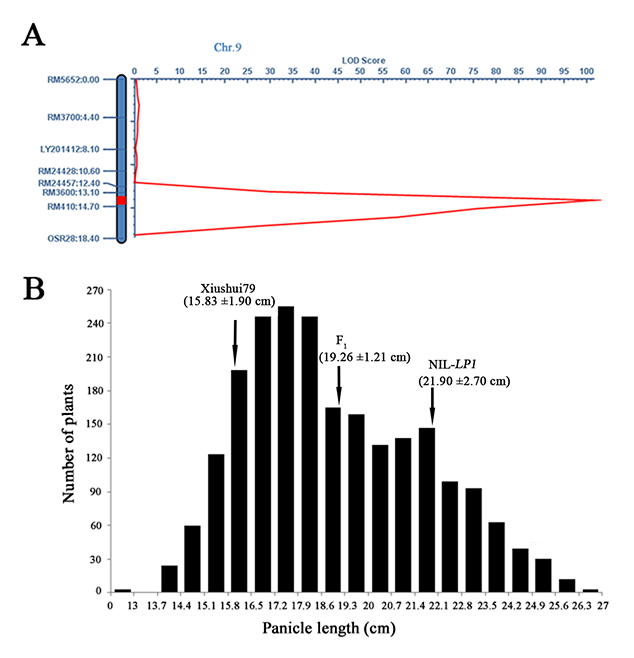

Supplement: Supplementary Figure 2 — Quantitative trait loci analysis using NIL-LP1/Xiushui79 F2 individuals. (A) Molecular linkage map of rice chromosome 9 showing the location of LP1. (B) Distribution of panicle length in the segregating population for the LP1 locus. [file Image2.TIF]

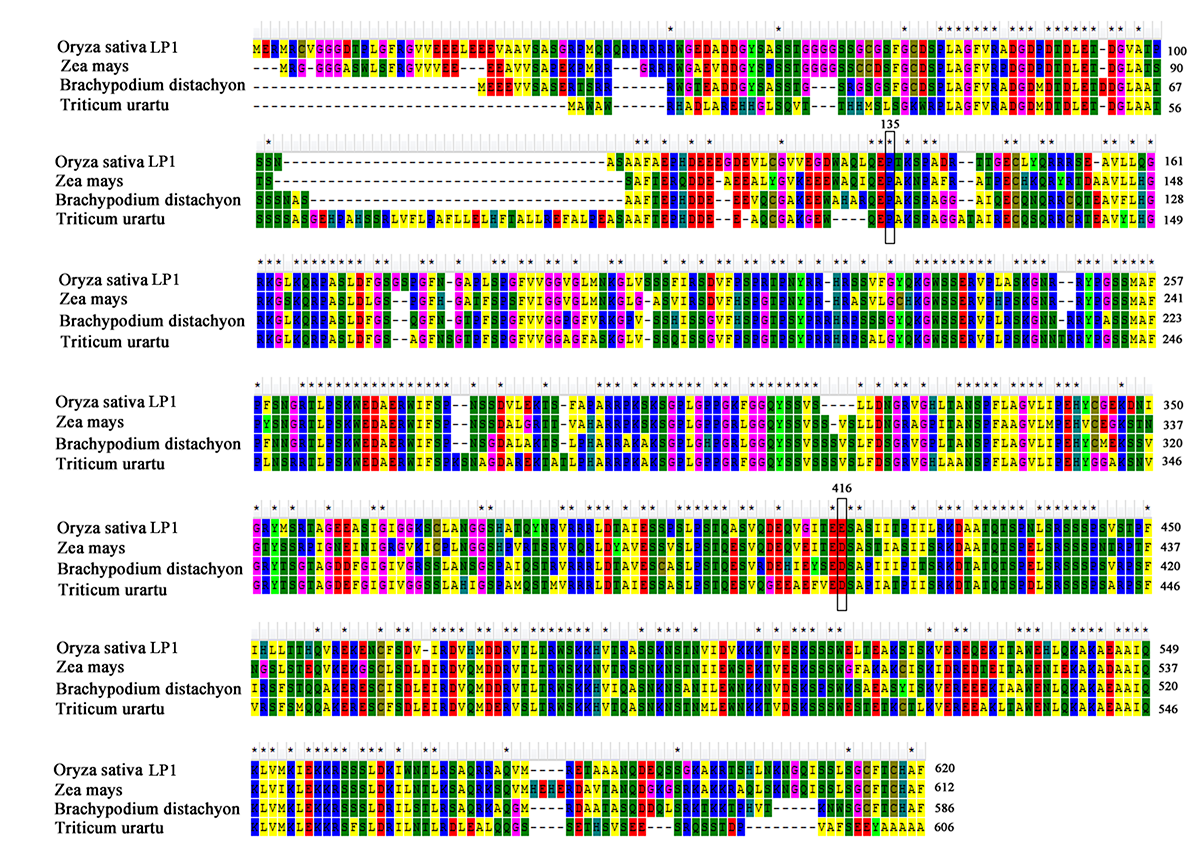

Supplement: Supplementary Figure 3 — Alignment of LP1 with the amino acid sequences of LP1-like proteins from other species. Asterisks indicate identical amino acids. [file Image3.TIF]
