# Supplementary material for: Identification of a Candidate Gene for Panicle Length in Rice (Oryza sativa L.) Via Association and Linkage Analysis
Source: Front Plant Sci. 2016 May 3;7:596. doi: 10.3389/fpls.2016.00596 (PMC4853638; doi:10.3389/fpls.2016.00596)
Supplement: Supplementary Table 1 — Panicle length of the 540 rice germplasm accessions, their geographical origin and their membership probabilities corresponding to each subpopulation. [file Table1.DOCX]

| **Supplementary Table 1.** Panicle length of the 540 rice germplasm accessions,their geographical origin and their membership probabilities corresponding to each subpopulation | | | | | | | | | | | |
| --- | --- | --- | --- | --- | --- | --- | --- | --- | --- | --- | --- |
|  |  |  |  |  |  |  |  |  |  |  |  |
| **Code** | **Accessions** | **Origin** | **Q1** | **Q2** | **Q3** | **Q4** | **Q5** | **Q6** | **Q7** | **Panicle length (cm)** | |
|  |  |  |  |  |  |  |  |  |  | **2011** | **2012** |
| **A1** | **Yazihuang** | Jinshan, Shanghai | 0.024 | 0.000 | 0.968 | 0.001 | 0.005 | 0.000 | 0.001 | 26.2 | 28.3 |
| **A2** | **Hongmangzaodao** | Kunshan, Jiangsu | 0.002 | 0.000 | 0.762 | 0.060 | 0.086 | 0.090 | 0.000 | 26.5 | 27.2 |
| A3 | Wanhuangdao | Wuxian, Jiangsu | 0.012 | 0.000 | 0.971 | 0.005 | 0.012 | 0.000 | 0.000 | 25.7 | 24.6 |
| A4 | Guozinuo | Kunshan, Jiangsu | 0.000 | 0.000 | 0.999 | 0.001 | 0.000 | 0.000 | 0.000 | 19.7 | 24.4 |
| A5 | Shuijingbaidao | Wuxian, Jiangsu | 0.000 | 0.000 | 0.999 | 0.000 | 0.000 | 0.000 | 0.000 | 21.5 | 22.3 |
| **A6** | **Wumangzaodao** | Changshu, Jiangsu | 0.000 | 0.000 | 0.972 | 0.009 | 0.016 | 0.000 | 0.002 | 29.3 | 29.8 |
| A7 | Sanbailitou | Kunshan, Jiangsu | 0.000 | 0.000 | 0.997 | 0.002 | 0.000 | 0.000 | 0.000 | 23.5 | 25.2 |
| A8 | Cuyingwanyangdao | Wuxi, Jiangsu | 0.000 | 0.000 | 0.999 | 0.000 | 0.000 | 0.000 | 0.000 | 23.4 | 25.1 |
| A9 | Yanglingdao | Wuxi, Jiangsu | 0.000 | 0.000 | 0.999 | 0.000 | 0.000 | 0.000 | 0.000 | 25.6 | 25 |
| A10 | Wanyedao | Wuxian, Jiangsu | 0.000 | 0.000 | 0.999 | 0.000 | 0.000 | 0.000 | 0.000 | 24.8 | 26.8 |
| **A11** | **Qiaobinghuang** | Taicang, Jiangsu | 0.000 | 0.000 | 0.999 | 0.000 | 0.000 | 0.000 | 0.000 | 28.4 | 28.5 |
| A12 | Tiejingqing | Kunshan, Jiangsu | 0.000 | 0.000 | 0.999 | 0.000 | 0.000 | 0.000 | 0.000 | 23 | 23.3 |
| A13 | Xiaobaiyedao | Wuxi, Jiangsu | 0.000 | 0.000 | 0.999 | 0.000 | 0.000 | 0.000 | 0.000 | 23.9 | 24.1 |
| **A14** | **Baoxintaihuqing** | Wujiang, Jiangsu | 0.010 | 0.001 | 0.814 | 0.070 | 0.066 | 0.038 | 0.000 | 24.6 | 26.1 |
| A15 | Jiangfeng4 | Jiangyin, Jiangsu | 0.001 | 0.000 | 0.986 | 0.000 | 0.000 | 0.013 | 0.000 | 21.8 | 24 |
| A16 | Sujing4 | Suzhou, Jiangsu | 0.000 | 0.000 | 0.986 | 0.000 | 0.009 | 0.000 | 0.004 | 20 | 18.1 |
| A17 | Aizhongluohanhuang | Changshu, Jiangsu | 0.000 | 0.000 | 0.999 | 0.000 | 0.000 | 0.000 | 0.000 | 23.8 | 26 |
| A18 | Baodao | Wuxi, Jiangsu | 0.000 | 0.000 | 0.999 | 0.000 | 0.000 | 0.000 | 0.000 | 18.4 | 18 |
| A19 | Wanmuxiqiu | Taicang, Jiangsu | 0.000 | 0.000 | 0.999 | 0.000 | 0.000 | 0.000 | 0.000 | 21.9 | 21.9 |
| A20 | Huangsanshi | Wujiang, Jiangsu | 0.000 | 0.000 | 0.999 | 0.000 | 0.000 | 0.000 | 0.000 | 21.6 | 21.2 |
| **A21** | **Erheidao** | Wuxi, Jiangsu | 0.000 | 0.000 | 0.999 | 0.000 | 0.000 | 0.000 | 0.000 | 26.4 | 27.3 |
| **A22** | **Xiaoqingzhong** | Wuxian, Jiangsu | 0.000 | 0.000 | 1.000 | 0.000 | 0.000 | 0.000 | 0.000 | 25.9 | 25.8 |
| A23 | Zaoguangtou | Wuxi, Jiangsu | 0.000 | 0.000 | 0.999 | 0.000 | 0.000 | 0.000 | 0.000 | 22.5 | 24.7 |
| **A24** | **Xiaoluohanhuang** | Changshu, Jiangsu | 0.000 | 0.000 | 0.999 | 0.000 | 0.000 | 0.000 | 0.000 | 25.7 | 23.8 |
| **A25** | **Souzhouqing** | Jiangyin, Jiangsu | 0.000 | 0.000 | 1.000 | 0.000 | 0.000 | 0.000 | 0.000 | 27.9 | 26.7 |
| A26 | Wanluli | Jiangyin, Jiangsu | 0.000 | 0.000 | 0.999 | 0.000 | 0.000 | 0.000 | 0.000 | 21.2 | 22 |
| A27 | Wanbaguo | Jiangyin, Jiangsu | 0.000 | 0.000 | 0.999 | 0.000 | 0.000 | 0.000 | 0.000 | 22.9 | 23.4 |
| A28 | Ebusinuodao | Wuxi, Jiangsu | 0.000 | 0.000 | 0.999 | 0.000 | 0.000 | 0.000 | 0.000 | 23.1 | 24.6 |
| A29 | Laodiegu | Wujiang, Jiangsu | 0.000 | 0.000 | 0.999 | 0.000 | 0.000 | 0.000 | 0.000 | 24.3 | 25.5 |
| A30 | Yefenghuang | Wujiang, Jiangsu | 0.000 | 0.000 | 1.000 | 0.000 | 0.000 | 0.000 | 0.000 | 23.4 | 25.8 |
| A31 | Chenjiazhong | Kunshan, Jiangsu | 0.000 | 0.000 | 1.000 | 0.000 | 0.000 | 0.000 | 0.000 | 25.7 | 26.6 |
| A32 | Zaoheitouhong | Wujiang, Jiangsu | 0.000 | 0.000 | 1.000 | 0.000 | 0.000 | 0.000 | 0.000 | 20 | 21.1 |
| **A33** | **Luohanhuang** | Jiangyin, Jiangsu | 0.000 | 0.000 | 0.999 | 0.000 | 0.000 | 0.000 | 0.000 | 27.2 | 27 |
| A34 | Longgouzhong | Qingpu, Shanghai | 0.000 | 0.000 | 1.000 | 0.000 | 0.000 | 0.000 | 0.000 | 25.5 | 25.4 |
| A35 | Shiluqing | Kunshan, Jiangsu | 0.000 | 0.000 | 1.000 | 0.000 | 0.000 | 0.000 | 0.000 | 24.1 | 23.4 |
| A36 | Ligengqing | Yixing, Jiangsu | 0.000 | 0.000 | 0.999 | 0.000 | 0.000 | 0.000 | 0.000 | 24 | 22.4 |
| A37 | Heitouhong | Wujiang, Jiangsu | 0.000 | 0.000 | 1.000 | 0.000 | 0.000 | 0.000 | 0.000 | 23.9 | 23.3 |
| A38 | Laolaihong | Wuxian, Jiangsu | 0.000 | 0.000 | 0.999 | 0.000 | 0.000 | 0.000 | 0.000 | 23.5 | 24.9 |
| A39 | Erlibie | Wuxian, Jiangsu | 0.000 | 0.000 | 1.000 | 0.000 | 0.000 | 0.000 | 0.000 | 25.3 | 28.1 |
| A40 | Jinguhuang | Wujiang, Jiangsu | 0.000 | 0.000 | 0.999 | 0.000 | 0.000 | 0.000 | 0.000 | 22.6 | 25.7 |
| A41 | Cuganhuangdao | Wujiang, Jiangsu | 0.000 | 0.000 | 1.000 | 0.000 | 0.000 | 0.000 | 0.000 | 23 | 23.5 |
| A42 | Zaoshirihuangdao | Wuxian, Jiangsu | 0.000 | 0.000 | 0.999 | 0.000 | 0.000 | 0.000 | 0.000 | 23 | 23.6 |
| A43 | Shengtangqing | Changshu, Jiangsu | 0.000 | 0.000 | 0.999 | 0.000 | 0.000 | 0.000 | 0.000 | 25.8 | 26.9 |
| **A44** | **Xiaomandao** | Wujiang, Jiangsu | 0.000 | 0.000 | 1.000 | 0.000 | 0.000 | 0.000 | 0.000 | 27.2 | 26.5 |
| A45 | Shengtangdao | Changshu, Jiangsu | 0.000 | 0.000 | 0.999 | 0.000 | 0.000 | 0.000 | 0.000 | 21.2 | 22.2 |
| **A46** | **Wanmandao** | Wujiang, Jiangsu | 0.000 | 0.000 | 0.999 | 0.000 | 0.000 | 0.000 | 0.000 | 28.4 | 27.8 |
| **A47** | **Nantouzhong** | Kunshan, Jiangsu | 0.000 | 0.000 | 0.999 | 0.000 | 0.000 | 0.000 | 0.000 | 26.4 | 27.6 |
| **A48** | **Daniaodao** | Changshu, Jiangsu | 0.000 | 0.000 | 0.997 | 0.000 | 0.002 | 0.000 | 0.000 | 29.2 | 27.6 |
| **A49** | **Kongqueqing** | Kunshan, Jiangsu | 0.000 | 0.001 | 0.938 | 0.004 | 0.006 | 0.034 | 0.017 | 26.3 | 27.8 |
| A50 | Kaiqing | Kunshan, Jiangsu | 0.001 | 0.000 | 0.944 | 0.000 | 0.001 | 0.044 | 0.010 | 22.6 | 19.1 |
| A51 | Manyedao | Kunshan, Jiangsu | 0.000 | 0.000 | 0.994 | 0.000 | 0.000 | 0.005 | 0.000 | 26.3 | 26.5 |
| **A52** | **Baikenuo** | Wujiang, Jiangsu | 0.000 | 0.001 | 0.888 | 0.000 | 0.000 | 0.109 | 0.000 | 27.6 | 27.9 |
| A53 | Baimangnuo | Wujiang, Jiangsu | 0.000 | 0.003 | 0.951 | 0.000 | 0.009 | 0.035 | 0.001 | 20.9 | 23.4 |
| A54 | Xiangzhunuo | Changshu, Jiangsu | 0.000 | 0.000 | 0.981 | 0.000 | 0.000 | 0.018 | 0.000 | 24.2 | 25.1 |
| A55 | Yaxienuo | Wuxian, Jiangsu | 0.000 | 0.000 | 0.982 | 0.000 | 0.000 | 0.017 | 0.000 | 25 | 24.7 |
| **A56** | **Xianhui429** | Nanjing, Jiangsu | 0.000 | 0.000 | 0.941 | 0.000 | 0.000 | 0.052 | 0.006 | 27.6 | 24.6 |
| **A57** | **Zijianxian3** | Nanjing, Jiangsu | 0.000 | 0.006 | 0.847 | 0.008 | 0.000 | 0.138 | 0.000 | 30.4 | 29.8 |
| A58 | Huangsandannuo | Wuxi, Jiangsu | 0.000 | 0.001 | 0.871 | 0.000 | 0.000 | 0.127 | 0.001 | 22.9 | 24 |
| **A59** | **Jia159** | Jiaxing, Zhejiang | 0.001 | 0.001 | 0.997 | 0.001 | 0.000 | 0.000 | 0.000 | 16.4 | 16.1 |
| A60 | Sidao10 | Siyang, Jiangsu | 0.000 | 0.000 | 0.999 | 0.000 | 0.000 | 0.000 | 0.000 | 24.1 | 24.5 |
| **A61** | **Wuqiang** | Wujin, Jiangsu | 0.000 | 0.000 | 0.999 | 0.000 | 0.000 | 0.000 | 0.000 | 16.4 | 17 |
| A62 | Wuyujing3 | Wujin, Jiangsu | 0.000 | 0.000 | 0.999 | 0.000 | 0.000 | 0.000 | 0.000 | 17 | 18.6 |
| A63 | Xiushui04 | Nanjing, Jiangsu | 0.000 | 0.000 | 0.999 | 0.000 | 0.000 | 0.000 | 0.000 | 17.5 | 17.9 |
| A64 | Zhendao88 | Zhenjiang, Jiangsu | 0.000 | 0.000 | 0.999 | 0.000 | 0.000 | 0.000 | 0.000 | 17.6 | 18.8 |
| A65 | Zhendao6 | Zhenjiang, Jiangsu | 0.000 | 0.000 | 0.999 | 0.000 | 0.000 | 0.000 | 0.000 | 21.2 | 18.9 |
| A66 | Taijing9 | Taibei,Tianwan | 0.000 | 0.000 | 0.999 | 0.000 | 0.000 | 0.000 | 0.000 | 23.9 | 26.2 |
| A67 | Taijing16xuanAC | Taibei,Tianwan | 0.000 | 0.000 | 0.999 | 0.000 | 0.000 | 0.000 | 0.000 | 23.8 | 25.6 |
| A68 | Taijing16xuanzi | Taibei,Tianwan | 0.000 | 0.000 | 0.993 | 0.000 | 0.000 | 0.006 | 0.000 | 25.1 | 26.4 |
| **A69** | **Diantun502xuanzao** | Kunming, Yunnan | 0.000 | 0.000 | 0.904 | 0.002 | 0.000 | 0.094 | 0.000 | 26.3 | 26.1 |
| **A70** | **Hongdao35** | Nanjing, Jiangsu | 0.000 | 0.000 | 0.999 | 0.000 | 0.000 | 0.000 | 0.000 | 30.5 | 32.8 |
| A71 | Hongdao37 | Nanjing, Jiangsu | 0.000 | 0.000 | 0.999 | 0.000 | 0.000 | 0.000 | 0.000 | 21.5 | 23.8 |
| A72 | Zijianjingnuo | Nanjing, Jiangsu | 0.000 | 0.000 | 0.999 | 0.000 | 0.000 | 0.000 | 0.000 | 18 | 18 |
| A73 | Nannongjing62401 | Nanjing, Jiangsu | 0.001 | 0.017 | 0.871 | 0.001 | 0.019 | 0.091 | 0.000 | 25.1 | 27.4 |
| **A74** | **Tongjing109** | Nantong, Jiangsu | 0.023 | 0.000 | 0.977 | 0.000 | 0.000 | 0.000 | 0.000 | 16.3 | 16.2 |
| A75 | Yangdao6 | Yangzhou, Jiangsu | 0.000 | 0.000 | 0.933 | 0.001 | 0.000 | 0.065 | 0.000 | 26.1 | 25.4 |
| A76 | Nignjing1 | Nanjing, Jiangsu | 0.000 | 0.000 | 0.999 | 0.000 | 0.000 | 0.000 | 0.000 | 18.2 | 17.8 |
| A77 | Wujing15 | Wujin, Jiangsu | 0.000 | 0.000 | 0.999 | 0.000 | 0.000 | 0.000 | 0.000 | 19.8 | 19.7 |
| A78 | Wuxiangjing14 | Wujin, Jiangsu | 0.000 | 0.000 | 0.999 | 0.000 | 0.000 | 0.000 | 0.000 | 19.1 | 20.6 |
| A79 | Xudao3 | Xuzhou, Jiangsu | 0.000 | 0.000 | 0.999 | 0.000 | 0.000 | 0.000 | 0.000 | 17.6 | 18.7 |
| **A80** | **Nannongjing003** | Nanjing, Jiangsu | 0.000 | 0.000 | 0.999 | 0.000 | 0.000 | 0.000 | 0.000 | 16.1 | 16.5 |
| A81 | Nannongjing005 | Nanjing, Jiangsu | 0.000 | 0.000 | 0.999 | 0.000 | 0.000 | 0.000 | 0.000 | 26.1 | 25 |
| A82 | 5jing20 | Nanjing, Jiangsu | 0.002 | 0.002 | 0.995 | 0.000 | 0.001 | 0.000 | 0.000 | 19 | 20.2 |
| A83 | 5jing15 | Nanjing, Jiangsu | 0.000 | 0.000 | 0.999 | 0.000 | 0.000 | 0.000 | 0.000 | 19.6 | 20.8 |
| A84 | molingjing | Nanjing, Jiangsu | 0.001 | 0.000 | 0.998 | 0.000 | 0.000 | 0.000 | 0.000 | 19.2 | 20.5 |
| A85 | 5jing03 | Nanjing, Jiangsu | 0.000 | 0.001 | 0.999 | 0.000 | 0.000 | 0.000 | 0.000 | 17.9 | 19.3 |
| A86 | 5jing68 | Nanjing, Jiangsu | 0.000 | 0.000 | 0.999 | 0.000 | 0.000 | 0.000 | 0.000 | 17.8 | 17.8 |
| A87 | Xudao4 | Xuzhou, Jiangsu | 0.000 | 0.000 | 0.999 | 0.000 | 0.000 | 0.000 | 0.000 | 19 | 18.4 |
| A88 | Xudao5 | Xuzhou, Jiangsu | 0.000 | 0.000 | 0.999 | 0.000 | 0.000 | 0.000 | 0.000 | 17.6 | 19.8 |
| A89 | Huaidao9 | Huaian, Jiangsu | 0.000 | 0.000 | 0.999 | 0.000 | 0.000 | 0.000 | 0.000 | 20.5 | 20.4 |
| A90 | Yandao6 | Yancheng, Jiangsu | 0.000 | 0.000 | 0.999 | 0.000 | 0.000 | 0.000 | 0.000 | 21.1 | 21.7 |
| A91 | Yangguang200 | Lianyungang, Jiangsu | 0.000 | 0.000 | 0.999 | 0.000 | 0.000 | 0.000 | 0.000 | 18 | 18.1 |
| A92 | Lianjing2 | Lianyungang, Jiangsu | 0.000 | 0.000 | 0.999 | 0.000 | 0.000 | 0.000 | 0.000 | 18.6 | 18 |
| **A93** | **Xiushui79** | Jiaxing, Zhejiang | 0.000 | 0.000 | 0.999 | 0.000 | 0.000 | 0.000 | 0.000 | 11.9 | 12.1 |
| **A94** | **C-bao** | Hefei, Anhui | 0.001 | 0.005 | 0.992 | 0.000 | 0.001 | 0.002 | 0.000 | 27.8 | 29.6 |
| A95 | Zhen9424 | Zhenjiang, Jiangsu | 0.032 | 0.266 | 0.693 | 0.000 | 0.000 | 0.001 | 0.008 | 17.1 | 17.3 |
| A96 | Wuyujing7 | Wujin, Jiangsu | 0.000 | 0.292 | 0.001 | 0.000 | 0.000 | 0.000 | 0.706 | 18.9 | 18.3 |
| A97 | Yanjing8 | Yancheng, Jiangsu | 0.000 | 0.000 | 0.000 | 0.000 | 0.000 | 0.000 | 0.999 | 17.3 | 17.5 |
| A98 | Zhengdao18 | Zhenzhou, Henan | 0.000 | 0.000 | 0.000 | 0.000 | 0.000 | 0.000 | 0.999 | 17.7 | 18.5 |
| A99 | Huaidao11 | Huaian, Jiangsu | 0.000 | 0.000 | 0.000 | 0.000 | 0.000 | 0.000 | 1.000 | 19.9 | 21 |
| A100 | Shengdao808 | Jiaxiang, Shandong | 0.000 | 0.000 | 0.000 | 0.000 | 0.000 | 0.000 | 1.000 | 17.9 | 18.4 |
| A101 | Shengdao14 | Jiaxiang, Shandong | 0.000 | 0.000 | 0.000 | 0.000 | 0.000 | 0.000 | 0.999 | 17.6 | 17.9 |
| **A102** | **Yujing6** | Zhenzhou, Henan | 0.000 | 0.000 | 0.000 | 0.000 | 0.000 | 0.000 | 1.000 | 16.5 | 17.7 |
| A103 | Huaidao8 | Huaian, Jiangsu | 0.000 | 0.000 | 0.000 | 0.000 | 0.000 | 0.000 | 0.999 | 16.8 | 19.4 |
| A104 | Jindao12 | Dongli, Tianjin | 0.000 | 0.000 | 0.000 | 0.000 | 0.000 | 0.000 | 0.999 | 21 | 21.4 |
| A105 | Wandao68 | Hefei, Anhui | 0.000 | 0.000 | 0.000 | 0.000 | 0.000 | 0.000 | 1.000 | 18.6 | 18.1 |
| A106 | Xudao2 | Xuzhou, Jiangsu | 0.000 | 0.000 | 0.000 | 0.000 | 0.000 | 0.001 | 0.998 | 24 | 24.4 |
| **A107** | **Sujing353** | Suzhou, Jiangsu | 0.000 | 0.000 | 0.000 | 0.000 | 0.000 | 0.000 | 0.999 | 25.1 | 26.8 |
| A108 | Xiangjing9407 | Nanjing, Jiangsu | 0.000 | 0.000 | 0.000 | 0.000 | 0.000 | 0.000 | 1.000 | 24.6 | 25.2 |
| A109 | Zhongjing212 | Nanjing, Jiangsu | 0.000 | 0.000 | 0.000 | 0.000 | 0.000 | 0.000 | 1.000 | 23.7 | 24.2 |
| A110 | Zhongjing9677 | Nanjing, Jiangsu | 0.000 | 0.000 | 0.000 | 0.000 | 0.000 | 0.000 | 1.000 | 20.3 | 23.3 |
| A111 | Zhongjing131 | Nanjing, Jiangsu | 0.000 | 0.000 | 0.000 | 0.000 | 0.000 | 0.000 | 1.000 | 22.1 | 22.8 |
| A112 | Suwujing | Wujin, Jiangsu | 0.000 | 0.000 | 0.000 | 0.000 | 0.000 | 0.000 | 1.000 | 20.2 | 21.2 |
| A113 | Zhognjing438 | Nanjing, Jiangsu | 0.000 | 0.000 | 0.000 | 0.000 | 0.000 | 0.000 | 1.000 | 21.3 | 22.9 |
| A114 | Yanjing9 | Yancheng, Jiangsu | 0.000 | 0.000 | 0.000 | 0.000 | 0.000 | 0.000 | 1.000 | 19.1 | 20.7 |
| A115 | Yangfujing4901 | Yangzhou, Jiangsu | 0.000 | 0.000 | 0.000 | 0.000 | 0.000 | 0.000 | 0.999 | 18.1 | 19.7 |
| **A116** | **Zijing** | Nanjing, Jiangsu | 0.000 | 0.000 | 0.000 | 0.000 | 0.000 | 0.000 | 1.000 | 16.3 | 16.4 |
| **A117** | **Zhendao10** | Zhenjiang, Jiangsu | 0.000 | 0.000 | 0.000 | 0.000 | 0.000 | 0.000 | 0.999 | 34.8 | 37.8 |
| A118 | Zhenghan2 | Zhengzhou, Henan | 0.000 | 0.000 | 0.000 | 0.000 | 0.000 | 0.000 | 1.000 | 17.8 | 19.6 |
| A119 | Zhen6 | Zhengzhou, Henan | 0.000 | 0.000 | 0.000 | 0.000 | 0.000 | 0.000 | 0.999 | 18.6 | 18.4 |
| A120 | Xishihuang | Wuxian, Jiangsu | 0.000 | 0.000 | 0.000 | 0.000 | 0.000 | 0.000 | 1.000 | 25.1 | 26.7 |
| A121 | Daliangdao | Wuxi, Jiangsu | 0.000 | 0.000 | 0.000 | 0.000 | 0.000 | 0.000 | 1.000 | 25.6 | 27.4 |
| A122 | Heizuidao | Changshu, Jiangsu | 0.000 | 0.000 | 0.000 | 0.000 | 0.000 | 0.000 | 1.000 | 25.2 | 26.1 |
| A123 | Xiaohuangdao | Wuxian, Jiangsu | 0.000 | 0.000 | 0.000 | 0.000 | 0.000 | 0.000 | 1.000 | 23.6 | 25.5 |
| **A124** | **Fenghaungdao** | Changshu, Jiangsu | 0.000 | 0.000 | 0.000 | 0.000 | 0.000 | 0.000 | 0.999 | 26.8 | 26.2 |
| A125 | Maijieqing | Songjiang, Shanghai | 0.000 | 0.000 | 0.000 | 0.000 | 0.000 | 0.000 | 1.000 | 25.3 | 25.3 |
| A126 | Jijingdao | Wujiang, Jiangsu | 0.000 | 0.000 | 0.000 | 0.000 | 0.000 | 0.000 | 1.000 | 24.3 | 25.1 |
| A127 | Zhognsuyangzhogndao | Wuxi, Jiangsu | 0.000 | 0.000 | 0.000 | 0.000 | 0.000 | 0.000 | 1.000 | 24.7 | 23.8 |
| A128 | Duiguzhong | Wujiang, Jiangsu | 0.000 | 0.000 | 0.000 | 0.000 | 0.000 | 0.000 | 1.000 | 23.5 | 23.3 |
| A129 | Shuaishaban | Songjiang, Shanghai | 0.000 | 0.000 | 0.000 | 0.000 | 0.000 | 0.000 | 1.000 | 25.5 | 24.8 |
| A130 | Niumaohuang | Taicang, Jiangsu | 0.000 | 0.000 | 0.000 | 0.000 | 0.000 | 0.000 | 0.999 | 22.3 | 22.5 |
| A131 | Wanheitouhong | Wujiang, Jiangsu | 0.000 | 0.000 | 0.000 | 0.000 | 0.000 | 0.000 | 0.999 | 22.6 | 22.6 |
| A132 | Taihuqing | Kunshan, Jiangsu | 0.000 | 0.000 | 0.000 | 0.000 | 0.000 | 0.000 | 0.999 | 22.6 | 22.4 |
| A133 | Yilimang | Changshu, Jiangsu | 0.000 | 0.000 | 0.000 | 0.000 | 0.000 | 0.000 | 0.999 | 22.6 | 23.2 |
| **A134** | **Wuqitou** | Wujin, Jiangsu | 0.000 | 0.000 | 0.000 | 0.000 | 0.000 | 0.000 | 0.999 | 27.1 | 29.6 |
| A135 | Jiaoaiheitouhong | Wujiang, Jiangsu | 0.000 | 0.000 | 0.000 | 0.000 | 0.000 | 0.000 | 0.999 | 25.1 | 25.6 |
| **A136** | **Laowusi** | Wujiang, Jiangsu | 0.000 | 0.000 | 0.000 | 0.000 | 0.000 | 0.000 | 0.999 | 27 | 26 |
| A137 | Manliuzhong | Jinshan, Shanghai | 0.000 | 0.000 | 0.000 | 0.000 | 0.000 | 0.000 | 0.999 | 23.7 | 22.7 |
| A138 | Tainluohuang | Changshu, Jiangsu | 0.000 | 0.000 | 0.000 | 0.000 | 0.000 | 0.000 | 0.999 | 22.4 | 24.4 |
| A139 | Jiucaiqing | Changshu, Jiangsu | 0.000 | 0.000 | 0.000 | 0.000 | 0.000 | 0.000 | 0.999 | 23 | 22 |
| A140 | Aiqidaliuzhong | Jiading, Shanghai | 0.000 | 0.000 | 0.000 | 0.000 | 0.000 | 0.000 | 0.999 | 23 | 23.2 |
| **A141** | **Lujingqing** | Wujiang, Jiangsu | 0.000 | 0.000 | 0.000 | 0.000 | 0.000 | 0.000 | 0.999 | 26.6 | 25.7 |
| **A142** | **Gaoliangqing** | Wujiang, Jiangsu | 0.000 | 0.000 | 0.000 | 0.000 | 0.000 | 0.000 | 0.999 | 25 | 25.1 |
| A143 | Yishixing | Changshu, Jiangsu | 0.000 | 0.000 | 0.000 | 0.000 | 0.000 | 0.000 | 0.999 | 25.7 | 25.3 |
| A144 | Heizhong | Wuxian, Jiangsu | 0.000 | 0.000 | 0.000 | 0.000 | 0.999 | 0.000 | 0.000 | 25.3 | 25.1 |
| A145 | Louhanbai | Kunshan, Jiangsu | 0.000 | 0.000 | 0.000 | 0.000 | 0.999 | 0.000 | 0.000 | 24.6 | 23.8 |
| A146 | Xueliqing | Wuxi, Jiangsu | 0.000 | 0.000 | 0.000 | 0.000 | 0.999 | 0.000 | 0.001 | 22.6 | 23.7 |
| **A147** | **Liyangxiaohongdao** | Liyang, Jiangsu | 0.000 | 0.001 | 0.000 | 0.000 | 0.998 | 0.000 | 0.000 | 29.5 | 29.7 |
| A148 | Juhuahuang | Wuxi, Jiangsu | 0.000 | 0.000 | 0.000 | 0.000 | 0.999 | 0.000 | 0.000 | 24.7 | 24.7 |
| A149 | Changzijingyedao | Wuxian, Jiangsu | 0.000 | 0.000 | 0.000 | 0.000 | 0.999 | 0.000 | 0.000 | 21.8 | 23.3 |
| **A150** | **Gaidaoqing** | Wujiang, Jiangsu | 0.000 | 0.000 | 0.000 | 0.000 | 0.845 | 0.154 | 0.000 | 25.6 | 26.7 |
| A151 | Dingzhuangdao | Wuxi, Jiangsu | 0.000 | 0.000 | 0.000 | 0.000 | 0.999 | 0.000 | 0.000 | 22.9 | 24.6 |
| A152 | Xuetangzhong | Jiangyin, Jiangsu | 0.000 | 0.000 | 0.000 | 0.000 | 0.999 | 0.000 | 0.000 | 22.5 | 25.2 |
| A153 | Guanbaidan | Wujiang, Jiangsu | 0.000 | 0.000 | 0.000 | 0.000 | 0.999 | 0.000 | 0.000 | 24.1 | 24 |
| A154 | Hongbaodao | Jiaxing, Zhejiang | 0.000 | 0.000 | 0.000 | 0.000 | 0.999 | 0.000 | 0.000 | 22.9 | 25.2 |
| A155 | Tiegandao | Wujiang, Jiangsu | 0.000 | 0.000 | 0.000 | 0.000 | 0.997 | 0.002 | 0.000 | 24.2 | 26 |
| A156 | Juzigaung | Wuxi, Jiangsu | 0.000 | 0.000 | 0.000 | 0.000 | 0.927 | 0.073 | 0.000 | 26.2 | 26.6 |
| A157 | Yebaidao | Taicang, Jiangsu | 0.000 | 0.000 | 0.000 | 0.000 | 0.999 | 0.000 | 0.000 | 23.3 | 24 |
| A158 | Daheitouhong | Wujiang, Jiangsu | 0.000 | 0.000 | 0.000 | 0.000 | 0.999 | 0.000 | 0.000 | 20.1 | 21 |
| **A159** | **Baigedao** | Wuxian, Jiangsu | 0.000 | 0.000 | 0.000 | 0.000 | 0.999 | 0.000 | 0.000 | 26.6 | 26.4 |
| A160 | Diediezhong | Qingpu, Shanghai | 0.000 | 0.000 | 0.000 | 0.000 | 0.999 | 0.000 | 0.000 | 23.6 | 23.7 |
| A161 | Puxidadaotou | Wujiang, Jiangsu | 0.000 | 0.000 | 0.000 | 0.000 | 0.999 | 0.000 | 0.000 | 25.7 | 26.3 |
| A162 | Yangdao | Wujiang, Jiangsu | 0.000 | 0.000 | 0.000 | 0.000 | 0.999 | 0.000 | 0.000 | 23.9 | 25.2 |
| A163 | Yanhongdao | Wujiang, Jiangsu | 0.424 | 0.000 | 0.000 | 0.000 | 0.576 | 0.000 | 0.000 | 19.8 | 20.9 |
| A164 | Baikewandao | Wuxi, Jiangsu | 0.000 | 0.000 | 0.000 | 0.000 | 0.999 | 0.000 | 0.000 | 24.1 | 24.2 |
| **A165** | **Aiguodadaotou** | Wujiang, Jiangsu | 0.000 | 0.000 | 0.000 | 0.000 | 0.999 | 0.000 | 0.000 | 27.3 | 27.1 |
| A166 | Sishitou | Wuxian, Jiangsu | 0.000 | 0.000 | 0.000 | 0.000 | 0.999 | 0.000 | 0.000 | 23.6 | 25 |
| A167 | Jiuxiaozhong | Wujiang, Jiangsu | 0.000 | 0.000 | 0.000 | 0.000 | 0.999 | 0.000 | 0.000 | 23.7 | 23.8 |
| A168 | Chushuhuang | Wuxian, Jiangsu | 0.000 | 0.000 | 0.000 | 0.000 | 0.999 | 0.000 | 0.000 | 26.2 | 27.2 |
| A169 | Qianjindao | Wujiang, Jiangsu | 0.000 | 0.000 | 0.000 | 0.000 | 0.999 | 0.000 | 0.000 | 25.4 | 25.7 |
| **A170** | **Qijiangqing** | Kunshan, Jiangsu | 0.000 | 0.000 | 0.000 | 0.000 | 0.999 | 0.000 | 0.000 | 29.7 | 28.8 |
| A171 | Baishuqing | Qingpu, Shanghai | 0.000 | 0.000 | 0.000 | 0.000 | 0.999 | 0.000 | 0.000 | 25.9 | 26 |
| A172 | Feilaifeng | Wuxi, Jiangsu | 0.000 | 0.000 | 0.000 | 0.000 | 0.999 | 0.000 | 0.000 | 25.8 | 27.4 |
| A173 | Kejia6 | Kunshan, Jiangsu | 0.000 | 0.000 | 0.000 | 0.000 | 0.999 | 0.000 | 0.000 | 22.3 | 22.9 |
| **A174** | **Lamujia** | Kunshan, Jiangsu | 0.000 | 0.000 | 0.000 | 0.000 | 0.999 | 0.000 | 0.000 | 26.5 | 29.7 |
| **A175** | **Haonuopie** | Kunshan, Jiangsu | 0.000 | 0.000 | 0.000 | 0.000 | 0.999 | 0.000 | 0.000 | 37.2 | 36.2 |
| **A176** | **Xiganggu** | Wujiang, Jiangsu | 0.000 | 0.000 | 0.000 | 0.000 | 0.999 | 0.000 | 0.000 | 26.9 | 27.2 |
| A177 | Shuangchengnuo | Wujiang, Jiangsu | 0.000 | 0.000 | 0.000 | 0.000 | 0.999 | 0.000 | 0.000 | 17.4 | 17.6 |
| A178 | Qiutiandaxiedao | Wujiang, Jiangsu | 0.000 | 0.000 | 0.000 | 0.000 | 0.999 | 0.000 | 0.000 | 19.8 | 20.4 |
| A179 | Qiyunuo10 | Wujiang, Jiangsu | 0.000 | 0.000 | 0.000 | 0.000 | 0.999 | 0.000 | 0.000 | 23 | 23.7 |
| A180 | Wunuo1 | Wujiang, Jiangsu | 0.000 | 0.000 | 0.000 | 0.000 | 0.999 | 0.000 | 0.000 | 17.7 | 17.5 |
| A181 | Jianongnuo2 | Wujiang, Jiangsu | 0.000 | 0.000 | 0.000 | 0.000 | 0.999 | 0.000 | 0.000 | 16.9 | 17.5 |
| A182 | Hongnong5 | Wujiang, Jiangsu | 0.000 | 0.000 | 0.000 | 0.000 | 0.999 | 0.000 | 0.000 | 24.8 | 25.7 |
| A183 | Nonglinnuo4 | Wujiang, Jiangsu | 0.000 | 0.015 | 0.000 | 0.000 | 0.983 | 0.002 | 0.000 | 20.4 | 22.4 |
| A184 | Xiangnuodao | Wuxian, Jiangsu | 0.000 | 0.012 | 0.002 | 0.000 | 0.984 | 0.002 | 0.000 | 22.8 | 24.4 |
| A185 | Luchaihong | Wujiang, Jiangsu | 0.000 | 0.000 | 0.000 | 0.000 | 0.999 | 0.000 | 0.000 | 24.6 | 25.3 |
| A186 | Cungu | Wujiang, Jiangsu | 0.000 | 0.000 | 0.000 | 0.001 | 0.926 | 0.071 | 0.000 | 24.2 | 25.2 |
| A187 | Katena | Wujiang, Jiangsu | 0.000 | 0.000 | 0.000 | 0.000 | 0.999 | 0.000 | 0.000 | 24.7 | 28 |
| **A188** | **Guanchanuo** | Wujiang, Jiangsu | 0.000 | 0.001 | 0.001 | 0.000 | 0.997 | 0.001 | 0.000 | 28.2 | 28 |
| **A189** | **Kuihuanuo** | Wuxian, Jiangsu | 0.000 | 0.000 | 0.000 | 0.000 | 0.924 | 0.074 | 0.000 | 24.7 | 27.1 |
| A190 | Suyunuo | Wuxian, Jiangsu | 0.000 | 0.001 | 0.000 | 0.000 | 0.998 | 0.000 | 0.000 | 18.5 | 22.2 |
| **A191** | **Hongjiaozhan** | Wuxian, Jiangsu | 0.000 | 0.000 | 0.000 | 0.005 | 0.895 | 0.100 | 0.000 | 30.2 | 31.8 |
| A192 | Haobuka | Wuxian, Jiangsu | 0.000 | 0.000 | 0.008 | 0.000 | 0.851 | 0.141 | 0.000 | 23 | 23.4 |
| **A193** | **Chuyanghan32** | Wuxian, Jiangsu | 0.000 | 0.000 | 0.003 | 0.000 | 0.868 | 0.128 | 0.000 | 25.5 | 26.8 |
| A194 | Libanyi | Wuxian, Jiangsu | 0.000 | 0.000 | 0.000 | 0.000 | 0.895 | 0.104 | 0.000 | 24.2 | 26.5 |
| A195 | Kunnong8 | Kunshan, Jiangsu | 0.001 | 0.003 | 0.004 | 0.000 | 0.978 | 0.015 | 0.000 | 17.5 | 19.9 |
| **A196** | **Guihuahuang** | Nanjing, Jiangsu | 0.000 | 0.000 | 0.000 | 0.000 | 0.999 | 0.000 | 0.000 | 18.1 | 21.2 |
| A197 | Zhoujiazhong | Wujiang, Jiangsu | 0.000 | 0.000 | 0.000 | 0.000 | 0.999 | 0.000 | 0.000 | 23.4 | 23.7 |
| A198 | Xiaofenghuang | Wuxian, Jiangsu | 0.000 | 0.000 | 0.000 | 0.000 | 0.999 | 0.000 | 0.000 | 23.3 | 24.3 |
| A199 | Xiangjingdao | Wuxian, Jiangsu | 0.000 | 0.000 | 0.000 | 0.000 | 0.999 | 0.000 | 0.000 | 23.9 | 24.5 |
| A200 | Huizao | Wujiang, Jiangsu | 0.000 | 0.000 | 0.000 | 0.000 | 0.999 | 0.000 | 0.000 | 23.6 | 25.3 |
| A201 | Yingtoudao | Kunshan, Jiangsu | 0.000 | 0.000 | 0.000 | 0.000 | 0.999 | 0.000 | 0.000 | 23 | 22.1 |
| A202 | Changdaotou | Wujiang, Jiangsu | 0.000 | 0.000 | 0.000 | 0.000 | 0.999 | 0.000 | 0.000 | 23.2 | 23.8 |
| A203 | Yangmiaozhong | Wujiang, Jiangsu | 0.000 | 0.000 | 0.000 | 0.000 | 0.999 | 0.000 | 0.000 | 22 | 22.6 |
| A204 | Maoguangdao | Wuxian, Jiangsu | 0.000 | 0.000 | 0.000 | 0.000 | 0.999 | 0.000 | 0.000 | 24.2 | 25.6 |
| A205 | Dazhongdao | Wujiang, Jiangsu | 0.000 | 0.000 | 0.000 | 0.000 | 0.999 | 0.000 | 0.000 | 22.2 | 23.8 |
| A206 | Sanxiadao | Wuxi, Jiangsu | 0.000 | 0.000 | 0.000 | 0.000 | 0.999 | 0.000 | 0.000 | 24.8 | 24.8 |
| A207 | Xiaoqingmang | Changshu, Jiangsu | 0.000 | 0.000 | 0.000 | 0.000 | 0.999 | 0.000 | 0.000 | 21.4 | 23.8 |
| A208 | Hongganlizhihong | Wujiang, Jiangsu | 0.000 | 0.000 | 0.000 | 0.000 | 0.999 | 0.000 | 0.000 | 24.4 | 26.2 |
| A209 | Wuxidao | Changshu, Jiangsu | 0.000 | 0.000 | 0.000 | 0.000 | 0.999 | 0.000 | 0.000 | 17 | 19.3 |
| A210 | Wanzhognqiu | Wuxian, Jiangsu | 0.000 | 0.000 | 0.000 | 0.000 | 0.999 | 0.000 | 0.000 | 24 | 24.8 |
| A211 | Fengjingdao | Wuxian, Jiangsu | 0.000 | 0.000 | 0.000 | 0.000 | 0.999 | 0.000 | 0.000 | 23.5 | 22.8 |
| A212 | Liuzhong | Changshu, Jiangsu | 0.000 | 0.000 | 0.000 | 0.000 | 0.999 | 0.000 | 0.000 | 25.2 | 25.9 |
| A213 | Cuganlizhihong | Wuxian, Jiangsu | 0.000 | 0.000 | 0.000 | 0.000 | 0.999 | 0.000 | 0.000 | 23.1 | 25.1 |
| A214 | Chiguwandao | Wujiang, Jiangsu | 0.000 | 0.000 | 0.000 | 0.000 | 0.999 | 0.000 | 0.000 | 22.4 | 23.5 |
| **A215** | **Jiaobaiyeqing** | Songjiang, Shanghai | 0.002 | 0.000 | 0.000 | 0.000 | 0.884 | 0.113 | 0.000 | 26.6 | 27.8 |
| A216 | Chiguhong | Wujiang, Jiangsu | 0.000 | 0.000 | 0.000 | 0.001 | 0.974 | 0.025 | 0.000 | 25.3 | 26.6 |
| A217 | Fanluoqing | Kunshan, Jiangsu | 0.000 | 0.000 | 0.000 | 0.000 | 0.999 | 0.000 | 0.000 | 22.2 | 22.6 |
| A218 | Zaoyedao | Kunshan, Jiangsu | 0.000 | 0.000 | 0.000 | 0.000 | 0.999 | 0.000 | 0.000 | 23.7 | 24.4 |
| A219 | Baidiegu | Wujiang, Jiangsu | 0.000 | 0.000 | 0.000 | 0.000 | 0.999 | 0.001 | 0.000 | 26.1 | 25.8 |
| **A220** | **Wangjiadao** | Wujiang, Jiangsu | 0.000 | 0.000 | 0.000 | 0.000 | 0.999 | 0.000 | 0.000 | 26.8 | 27.2 |
| A221 | Jiangyinzhong | Jiangyin, Jiangsu | 0.000 | 0.000 | 0.000 | 0.000 | 0.999 | 0.000 | 0.000 | 24.4 | 24.7 |
| A222 | Eyingbaijingdao | Jiading, Shanghai | 0.000 | 0.000 | 0.000 | 0.000 | 0.999 | 0.000 | 0.000 | 20.9 | 21.9 |
| A223 | Tiekewanguangtou | Wujin, Jiangsu | 0.000 | 0.000 | 0.000 | 0.000 | 0.999 | 0.000 | 0.000 | 21.4 | 21.9 |
| A224 | Tiekedao | Wujin, Jiangsu | 0.000 | 0.000 | 0.000 | 0.000 | 0.999 | 0.000 | 0.000 | 21.5 | 22.6 |
| A225 | Dadaosuitou | Changshu, Jiangsu | 0.000 | 0.000 | 0.000 | 0.000 | 0.999 | 0.000 | 0.000 | 22.8 | 23.1 |
| A226 | Aibaidao | Wujiang, Jiangsu | 0.000 | 0.000 | 0.000 | 0.000 | 1.000 | 0.000 | 0.000 | 24 | 24.1 |
| A227 | Xiepihuang | Taicang, Jiangsu | 0.000 | 0.000 | 0.000 | 0.000 | 0.999 | 0.000 | 0.000 | 25 | 23 |
| A228 | Xiaobaidao | Wuxian, Jiangsu | 0.000 | 0.000 | 0.000 | 0.000 | 0.999 | 0.000 | 0.000 | 23.6 | 25.8 |
| A229 | Baishidao | Taicang, Jiangsu | 0.000 | 0.000 | 0.000 | 0.000 | 0.999 | 0.000 | 0.000 | 23.7 | 23.6 |
| **A230** | **Manbaidao** | Wujiang, Jiangsu | 0.000 | 0.000 | 0.000 | 0.000 | 0.999 | 0.000 | 0.000 | 15.4 | 17.1 |
| A231 | Guangtouluhuabai | Wuxi, Jiangsu | 0.000 | 0.000 | 0.000 | 0.000 | 0.999 | 0.000 | 0.000 | 22 | 21.9 |
| A232 | Hongmangjing | Kunshan, Jiangsu | 0.000 | 0.000 | 0.000 | 0.000 | 0.999 | 0.000 | 0.000 | 22.1 | 23.3 |
| A233 | Wumangyedao | Changshu, Jiangsu | 0.000 | 0.000 | 0.000 | 0.000 | 0.999 | 0.000 | 0.000 | 23.2 | 25.4 |
| A234 | Luhuabai | Wuxian, Jiangsu | 0.000 | 0.000 | 0.000 | 0.000 | 0.999 | 0.000 | 0.000 | 23.1 | 24 |
| A235 | Haidongqing | Kunshan, Jiangsu | 0.000 | 0.000 | 0.000 | 0.000 | 0.999 | 0.000 | 0.000 | 25.6 | 24.4 |
| A236 | Shenlenuo | Kunshan, Jiangsu | 0.000 | 0.000 | 0.000 | 0.000 | 0.999 | 0.000 | 0.000 | 25.1 | 24.5 |
| A237 | Xiangqing | Chongming, Shanghai | 0.000 | 0.000 | 0.000 | 0.000 | 0.952 | 0.048 | 0.000 | 24.6 | 23.5 |
| **A238** | **Jinghui418** | Shenyang, Liaoning | 0.000 | 0.000 | 0.000 | 0.002 | 0.914 | 0.083 | 0.000 | 33.1 | 33.9 |
| A239 | Malaihong | Nanjing, Jiangsu | 0.001 | 0.000 | 0.000 | 0.000 | 0.834 | 0.165 | 0.000 | 23.5 | 26.9 |
| A240 | Jingnuo330 | Hefei, Anhui | 0.751 | 0.000 | 0.000 | 0.000 | 0.248 | 0.000 | 0.000 | 20.7 | 19.9 |
| A241 | Zaijinjing | Huaian, Jiangsu | 0.729 | 0.000 | 0.000 | 0.000 | 0.270 | 0.000 | 0.000 | 21.3 | 21.8 |
| A242 | Fuyu3 | Yuexi, Anhui | 0.718 | 0.000 | 0.000 | 0.000 | 0.117 | 0.165 | 0.000 | 24.8 | 24.3 |
| **A243** | **Dongnongjing424** | Shenyang, Liaoning | 1.000 | 0.000 | 0.000 | 0.000 | 0.000 | 0.000 | 0.000 | 18.8 | 17.2 |
| A244 | Dongnongjingnuo418 | Shenyang, Liaoning | 0.780 | 0.000 | 0.000 | 0.000 | 0.219 | 0.001 | 0.000 | 19.3 | 20.4 |
| A245 | R254 | Chongming, Shanghai | 0.783 | 0.000 | 0.000 | 0.000 | 0.216 | 0.001 | 0.000 | 21 | 21.6 |
| A246 | Jiangyinnuo | Jiangyin, Jiangsu | 0.802 | 0.000 | 0.000 | 0.000 | 0.197 | 0.000 | 0.000 | 20.6 | 23.3 |
| A247 | Jinggunuo | Wuxi, Jiangsu | 0.760 | 0.001 | 0.000 | 0.001 | 0.238 | 0.000 | 0.000 | 23.9 | 24.4 |
| **A248** | **Shanhonggu** | Wujiang, Jiangsu | 0.786 | 0.000 | 0.000 | 0.000 | 0.213 | 0.000 | 0.000 | 28.9 | 27.9 |
| A249 | Wanshengmaohuang | Wuxi, Jiangsu | 0.765 | 0.000 | 0.000 | 0.000 | 0.235 | 0.000 | 0.000 | 22.2 | 24.1 |
| A250 | Wanyangdao | Wuxian, Jiangsu | 0.771 | 0.000 | 0.000 | 0.000 | 0.228 | 0.000 | 0.000 | 23.2 | 23.6 |
| A251 | Aidazhong | Wujiang, Jiangsu | 0.759 | 0.000 | 0.000 | 0.000 | 0.241 | 0.000 | 0.000 | 22.2 | 24.5 |
| **A252** | **Jijiaohong** | Wuxian, Jiangsu | 0.793 | 0.000 | 0.000 | 0.000 | 0.206 | 0.001 | 0.000 | 24.9 | 25.2 |
| A253 | Toulaizhong | Wujiang, Jiangsu | 1.000 | 0.000 | 0.000 | 0.000 | 0.000 | 0.000 | 0.000 | 21.2 | 21.3 |
| A254 | Huakenuo | Wujiang, Jiangsu | 1.000 | 0.000 | 0.000 | 0.000 | 0.000 | 0.000 | 0.000 | 25 | 24.2 |
| **A255** | **Toudengyishixing** | Kunshan, Jiangsu | 1.000 | 0.000 | 0.000 | 0.000 | 0.000 | 0.000 | 0.000 | 14.6 | 14.8 |
| A256 | Maozitou | Wujiang, Jiangsu | 1.000 | 0.000 | 0.000 | 0.000 | 0.000 | 0.000 | 0.000 | 24.4 | 26.2 |
| A257 | Zaonuodao | Wujiang, Jiangsu | 1.000 | 0.000 | 0.000 | 0.000 | 0.000 | 0.000 | 0.000 | 18.5 | 18.7 |
| **A258** | **Datougui** | Changshu, Jiangsu | 0.999 | 0.000 | 0.000 | 0.000 | 0.000 | 0.000 | 0.000 | 17.4 | 18.8 |
| **A259** | **Zaoxiaobaidao** | Wuxi, Jiangsu | 1.000 | 0.000 | 0.000 | 0.000 | 0.000 | 0.000 | 0.000 | 16.5 | 18.5 |
| A260 | Kangzhounuo | Wujiang, Jiangsu | 1.000 | 0.000 | 0.000 | 0.000 | 0.000 | 0.000 | 0.000 | 26 | 26.9 |
| A261 | Kuobanzhong | Qingpu, Shanghai | 1.000 | 0.000 | 0.000 | 0.000 | 0.000 | 0.000 | 0.000 | 26.3 | 26.8 |
| A262 | Yangzhongdao | Wujiang, Jiangsu | 1.000 | 0.000 | 0.000 | 0.000 | 0.000 | 0.000 | 0.000 | 24.3 | 25.9 |
| A263 | Huangkewanguangtou | Wujin, Jiangsu | 1.000 | 0.000 | 0.000 | 0.000 | 0.000 | 0.000 | 0.000 | 25.8 | 26.7 |
| A264 | Tiehanyishixing | Wuxi, Jiangsu | 1.000 | 0.000 | 0.000 | 0.000 | 0.000 | 0.000 | 0.000 | 17.5 | 17.9 |
| A265 | Aijiaoluganhuang | Changshu, Jiangsu | 1.000 | 0.000 | 0.000 | 0.000 | 0.000 | 0.000 | 0.000 | 18 | 18.6 |
| A266 | Zhonghua3 | Haidian, Beijing | 1.000 | 0.000 | 0.000 | 0.000 | 0.000 | 0.000 | 0.000 | 17.4 | 18.1 |
| **A267** | **Buxienuo** | kunshan, Jiangsu | 1.000 | 0.000 | 0.000 | 0.000 | 0.000 | 0.000 | 0.000 | 28.3 | 28.3 |
| **A268** | **Wanjingnuo** | Hefei, Anhui | 1.000 | 0.000 | 0.000 | 0.000 | 0.000 | 0.000 | 0.000 | 17.3 | 17 |
| **A269** | **C418** | Shenyang, Liaoning | 1.000 | 0.000 | 0.000 | 0.000 | 0.000 | 0.000 | 0.000 | 33 | 33.8 |
| **A270** | **Fuxiang1** | Yuexi, Anhui | 1.000 | 0.000 | 0.000 | 0.000 | 0.000 | 0.000 | 0.000 | 28.1 | 28.9 |
| A271 | Yuedao1 | Vietnam | 0.747 | 0.000 | 0.000 | 0.000 | 0.000 | 0.253 | 0.000 | 27.1 | 26.4 |
| A272 | Yuedao2 | Vietnam | 0.688 | 0.000 | 0.000 | 0.000 | 0.000 | 0.311 | 0.000 | 25.2 | 27.1 |
| A273 | Yuedao3 | Vietnam | 0.703 | 0.000 | 0.000 | 0.000 | 0.000 | 0.296 | 0.000 | 25.7 | 26.4 |
| A274 | Yuedao4 | Vietnam | 0.712 | 0.000 | 0.000 | 0.000 | 0.000 | 0.287 | 0.000 | 26.5 | 26.8 |
| A275 | Yuedao5 | Vietnam | 0.672 | 0.000 | 0.000 | 0.000 | 0.000 | 0.327 | 0.000 | 27.1 | 26.1 |
| A276 | Yuedao6 | Vietnam | 0.693 | 0.000 | 0.000 | 0.000 | 0.000 | 0.307 | 0.000 | 25.8 | 29.2 |
| A277 | Yuedao7 | Vietnam | 0.664 | 0.000 | 0.000 | 0.000 | 0.000 | 0.335 | 0.000 | 27.8 | 27 |
| A278 | Yuedao8 | Vietnam | 0.663 | 0.000 | 0.000 | 0.000 | 0.000 | 0.337 | 0.000 | 33.7 | 34.2 |
| A279 | Yuedao9 | Vietnam | 0.672 | 0.000 | 0.000 | 0.000 | 0.000 | 0.328 | 0.000 | 29.3 | 33 |
| A280 | Yuedao10 | Vietnam | 0.681 | 0.000 | 0.000 | 0.000 | 0.000 | 0.319 | 0.000 | 27 | 27.6 |
| A281 | Yuedao11 | Vietnam | 0.679 | 0.000 | 0.000 | 0.000 | 0.000 | 0.320 | 0.000 | 26.1 | 27.5 |
| A282 | Yuedao12 | Vietnam | 0.656 | 0.000 | 0.000 | 0.000 | 0.000 | 0.343 | 0.000 | 24.6 | 26.9 |
| A283 | Yuedao13 | Vietnam | 0.685 | 0.000 | 0.000 | 0.000 | 0.000 | 0.314 | 0.000 | 31.1 | 32.9 |
| A284 | Yuedao14 | Vietnam | 0.664 | 0.000 | 0.000 | 0.000 | 0.000 | 0.336 | 0.000 | 26.4 | 26.8 |
| A285 | Yuedao15 | Vietnam | 0.684 | 0.000 | 0.000 | 0.000 | 0.000 | 0.316 | 0.000 | 24.3 | 24.9 |
| A286 | Yuedao16 | Vietnam | 0.684 | 0.000 | 0.000 | 0.000 | 0.000 | 0.316 | 0.000 | 26.8 | 26.8 |
| A287 | Yuedao17 | Vietnam | 0.670 | 0.000 | 0.000 | 0.000 | 0.000 | 0.330 | 0.000 | 24.8 | 26.5 |
| **A288** | **Yuedao18** | Vietnam | 0.000 | 0.000 | 0.000 | 0.000 | 0.000 | 0.999 | 0.000 | 28.2 | 28.9 |
| A289 | Yuedao19 | Vietnam | 0.000 | 0.000 | 0.000 | 0.000 | 0.000 | 0.999 | 0.000 | 26.6 | 27.3 |
| A290 | Yuedao20 | Vietnam | 0.000 | 0.000 | 0.000 | 0.000 | 0.000 | 0.999 | 0.000 | 28.1 | 28 |
| A291 | Yuedao21 | Vietnam | 0.000 | 0.000 | 0.000 | 0.000 | 0.000 | 0.999 | 0.000 | 27.6 | 27.2 |
| A292 | Yuedao22 | Vietnam | 0.000 | 0.000 | 0.000 | 0.000 | 0.000 | 0.999 | 0.000 | 29.5 | 29.8 |
| A293 | Yuedao23 | Vietnam | 0.000 | 0.000 | 0.005 | 0.000 | 0.000 | 0.994 | 0.000 | 31.5 | 31.9 |
| A294 | Yuedao24 | Vietnam | 0.000 | 0.000 | 0.000 | 0.000 | 0.000 | 0.999 | 0.000 | 31.1 | 35 |
| **A295** | **Yuedao25** | Vietnam | 0.000 | 0.000 | 0.000 | 0.000 | 0.000 | 0.999 | 0.000 | 32.6 | 35.3 |
| A296 | Yuedao26 | Vietnam | 0.000 | 0.000 | 0.000 | 0.000 | 0.000 | 0.999 | 0.000 | 30.9 | 31.8 |
| **A297** | **Yuedao27** | Vietnam | 0.000 | 0.000 | 0.000 | 0.000 | 0.000 | 1.000 | 0.000 | 32.8 | 32.8 |
| A298 | Yuedao28 | Vietnam | 0.000 | 0.000 | 0.000 | 0.000 | 0.000 | 1.000 | 0.000 | 32.5 | 34.2 |
| A299 | Yuedao29 | Vietnam | 0.000 | 0.000 | 0.000 | 0.000 | 0.000 | 0.999 | 0.000 | 26.1 | 26.5 |
| A300 | Yuedao30 | Vietnam | 0.000 | 0.000 | 0.000 | 0.000 | 0.000 | 1.000 | 0.000 | 30.1 | 30.5 |
| A301 | Yuedao31 | Vietnam | 0.000 | 0.000 | 0.000 | 0.000 | 0.000 | 1.000 | 0.000 | 32.8 | 33.8 |
| A302 | Yuedao32 | Vietnam | 0.000 | 0.000 | 0.000 | 0.000 | 0.000 | 1.000 | 0.000 | 31.3 | 33.8 |
| **A303** | **Yuedao33** | Vietnam | 0.000 | 0.000 | 0.000 | 0.000 | 0.000 | 0.999 | 0.000 | 33.6 | 35 |
| A304 | Yuedao34 | Vietnam | 0.000 | 0.000 | 0.000 | 0.000 | 0.000 | 1.000 | 0.000 | 33.1 | 33.9 |
| A305 | Yuedao35 | Vietnam | 0.000 | 0.000 | 0.000 | 0.000 | 0.000 | 0.999 | 0.000 | 28.2 | 31.9 |
| A306 | Yuedao36 | Vietnam | 0.000 | 0.000 | 0.000 | 0.000 | 0.000 | 1.000 | 0.000 | 30.9 | 31.5 |
| A307 | Yuedao37 | Vietnam | 0.000 | 0.000 | 0.000 | 0.000 | 0.000 | 1.000 | 0.000 | 31 | 31.3 |
| A308 | Yuedao38 | Vietnam | 0.000 | 0.000 | 0.000 | 0.000 | 0.000 | 1.000 | 0.000 | 31.9 | 32.3 |
| A309 | Yuedao39 | Vietnam | 0.000 | 0.000 | 0.000 | 0.000 | 0.000 | 1.000 | 0.000 | 31.6 | 31.3 |
| A310 | Yuedao40 | Vietnam | 0.000 | 0.000 | 0.000 | 0.000 | 0.000 | 1.000 | 0.000 | 31.5 | 32.3 |
| A311 | Yuedao41 | Vietnam | 0.000 | 0.000 | 0.000 | 0.000 | 0.000 | 0.999 | 0.000 | 30.8 | 32.8 |
| A312 | Yuedao42 | Vietnam | 0.000 | 0.000 | 0.000 | 0.000 | 0.000 | 1.000 | 0.000 | 27.9 | 32.4 |
| A313 | Yuedao43 | Vietnam | 0.000 | 0.000 | 0.000 | 0.000 | 0.000 | 0.999 | 0.000 | 30.6 | 30.9 |
| A314 | Yuedao44 | Vietnam | 0.000 | 0.000 | 0.000 | 0.000 | 0.000 | 1.000 | 0.000 | 30.5 | 30.2 |
| **A315** | **Yuedao45** | Vietnam | 0.000 | 0.000 | 0.000 | 0.000 | 0.000 | 0.999 | 0.000 | 34.6 | 35.4 |
| **A316** | **Yuedao46** | Vietnam | 0.000 | 0.000 | 0.000 | 0.000 | 0.000 | 0.999 | 0.000 | 28.7 | 27.5 |
| A317 | Yuedao47 | Vietnam | 0.000 | 0.000 | 0.000 | 0.000 | 0.000 | 0.999 | 0.000 | 27.8 | 30.8 |
| A318 | Yuedao48 | Vietnam | 0.000 | 0.000 | 0.000 | 0.000 | 0.000 | 0.999 | 0.000 | 27.7 | 28.2 |
| A319 | Yuedao49 | Vietnam | 0.000 | 0.000 | 0.000 | 0.000 | 0.000 | 0.999 | 0.000 | 25.8 | 25.8 |
| A320 | Yuedao50 | Vietnam | 0.000 | 0.000 | 0.000 | 0.000 | 0.000 | 1.000 | 0.000 | 30.1 | 30.9 |
| A321 | Yuedao51 | Vietnam | 0.000 | 0.000 | 0.000 | 0.000 | 0.000 | 0.999 | 0.000 | 29.8 | 30.3 |
| A322 | Yuedao52 | Vietnam | 0.000 | 0.000 | 0.000 | 0.000 | 0.000 | 0.999 | 0.000 | 26.6 | 28.3 |
| A323 | Yuedao53 | Vietnam | 0.000 | 0.000 | 0.000 | 0.000 | 0.000 | 0.999 | 0.000 | 25.7 | 26.2 |
| A324 | Yuedao54 | Vietnam | 0.417 | 0.000 | 0.000 | 0.000 | 0.000 | 0.582 | 0.000 | 27.3 | 28.6 |
| A325 | Yuedao55 | Vietnam | 0.001 | 0.032 | 0.000 | 0.000 | 0.019 | 0.941 | 0.007 | 27.8 | 27.7 |
| A326 | Yuedao56 | Vietnam | 0.000 | 0.001 | 0.001 | 0.000 | 0.000 | 0.998 | 0.000 | 25.9 | 24.5 |
| A327 | Yuedao57 | Vietnam | 0.000 | 0.000 | 0.000 | 0.000 | 0.000 | 0.999 | 0.000 | 30.3 | 31.2 |
| A328 | Yuedao58 | Vietnam | 0.000 | 0.000 | 0.000 | 0.000 | 0.000 | 0.999 | 0.000 | 26.4 | 27.6 |
| A329 | Yuedao59 | Vietnam | 0.000 | 0.000 | 0.000 | 0.000 | 0.000 | 0.999 | 0.000 | 26.5 | 27.6 |
| A330 | Yuedao60 | Vietnam | 0.000 | 0.000 | 0.000 | 0.000 | 0.000 | 0.999 | 0.000 | 26.4 | 27.4 |
| A331 | Yuedao61 | Vietnam | 0.000 | 0.000 | 0.000 | 0.000 | 0.000 | 0.999 | 0.000 | 29.8 | 33.6 |
| **A332** | **Yuedao62** | Vietnam | 0.000 | 0.000 | 0.000 | 0.000 | 0.000 | 0.999 | 0.000 | 29.2 | 30.2 |
| **A333** | **Yuedao63** | Vietnam | 0.001 | 0.001 | 0.000 | 0.000 | 0.009 | 0.988 | 0.001 | 28.1 | 27.4 |
| **A334** | **Yuedao64** | Vietnam | 0.000 | 0.062 | 0.000 | 0.000 | 0.012 | 0.923 | 0.002 | 27.1 | 27.4 |
| **A335** | **Yuedao65** | Vietnam | 0.000 | 0.000 | 0.000 | 0.000 | 0.000 | 0.999 | 0.000 | 33.2 | 33.6 |
| A336 | Yuedao66 | Vietnam | 0.000 | 0.055 | 0.005 | 0.002 | 0.010 | 0.914 | 0.015 | 25.6 | 28.8 |
| A337 | Yuedao67 | Vietnam | 0.000 | 0.000 | 0.000 | 0.000 | 0.000 | 0.999 | 0.000 | 28 | 30 |
| A338 | Yuedao68 | Vietnam | 0.000 | 0.000 | 0.000 | 0.000 | 0.000 | 0.999 | 0.001 | 23.9 | 25.8 |
| A339 | Yuedao69 | Vietnam | 0.000 | 0.000 | 0.000 | 0.000 | 0.000 | 0.999 | 0.000 | 25.6 | 27.4 |
| A340 | Yuedao70 | Vietnam | 0.000 | 0.000 | 0.000 | 0.000 | 0.000 | 0.999 | 0.000 | 26.3 | 30.3 |
| A341 | Yuedao71 | Vietnam | 0.000 | 0.000 | 0.000 | 0.000 | 0.000 | 0.999 | 0.000 | 28 | 29.6 |
| A342 | Yuedao72 | Vietnam | 0.000 | 0.000 | 0.000 | 0.000 | 0.000 | 0.999 | 0.000 | 26.7 | 26.6 |
| **A343** | **Yuedao73** | Vietnam | 0.000 | 0.000 | 0.000 | 0.000 | 0.000 | 0.999 | 0.000 | 27.5 | 28.4 |
| A344 | Yuedao74 | Vietnam | 0.000 | 0.000 | 0.000 | 0.000 | 0.000 | 0.999 | 0.000 | 26.6 | 25.2 |
| **A345** | **Yuedao75** | Vietnam | 0.000 | 0.000 | 0.000 | 0.000 | 0.000 | 0.999 | 0.000 | 27.5 | 30 |
| A346 | Yuedao76 | Vietnam | 0.000 | 0.000 | 0.000 | 0.000 | 0.000 | 0.999 | 0.000 | 32.4 | 33.9 |
| A347 | Yuedao77 | Vietnam | 0.000 | 0.000 | 0.000 | 0.000 | 0.000 | 0.999 | 0.000 | 27.3 | 28.4 |
| A348 | Yuedao78 | Vietnam | 0.000 | 0.014 | 0.001 | 0.000 | 0.053 | 0.919 | 0.013 | 26.4 | 25 |
| A349 | Yuedao79 | Vietnam | 0.000 | 0.000 | 0.000 | 0.000 | 0.000 | 0.999 | 0.000 | 27 | 25.1 |
| A350 | Yuedao80 | Vietnam | 0.000 | 0.000 | 0.000 | 0.000 | 0.000 | 0.999 | 0.000 | 26.4 | 28.1 |
| A351 | Yuedao81 | Vietnam | 0.000 | 0.000 | 0.000 | 0.000 | 0.000 | 0.999 | 0.000 | 25.9 | 29.6 |
| A352 | Yuedao82 | Vietnam | 0.000 | 0.000 | 0.000 | 0.000 | 0.000 | 0.999 | 0.000 | 28.3 | 31.7 |
| A353 | Yuedao83 | Vietnam | 0.000 | 0.000 | 0.000 | 0.000 | 0.000 | 0.999 | 0.000 | 27.6 | 28 |
| A354 | Yuedao84 | Vietnam | 0.409 | 0.000 | 0.000 | 0.000 | 0.001 | 0.588 | 0.001 | 33.4 | 32.8 |
| A355 | Yuedao85 | Vietnam | 0.000 | 0.000 | 0.000 | 0.000 | 0.000 | 0.999 | 0.000 | 27.9 | 29.3 |
| **A356** | **Yuedao86** | Vietnam | 0.000 | 0.028 | 0.032 | 0.000 | 0.056 | 0.883 | 0.001 | 35.6 | 32.4 |
| **A357** | **Yuedao87** | Vietnam | 0.000 | 0.000 | 0.000 | 0.000 | 0.000 | 0.999 | 0.000 | 34.8 | 35.8 |
| A358 | Yuedao88 | Vietnam | 0.000 | 0.000 | 0.000 | 0.000 | 0.000 | 1.000 | 0.000 | 29.5 | 32.4 |
| A359 | Yuedao89 | Vietnam | 0.000 | 0.000 | 0.000 | 0.000 | 0.000 | 0.999 | 0.000 | 31.9 | 34.1 |
| A360 | Yuedao90 | Vietnam | 0.000 | 0.000 | 0.000 | 0.000 | 0.000 | 0.999 | 0.000 | 30.8 | 32.2 |
| A361 | Yuedao91 | Vietnam | 0.000 | 0.000 | 0.002 | 0.000 | 0.012 | 0.985 | 0.000 | 23.2 | 26.4 |
| A362 | Yuedao92 | Vietnam | 0.000 | 0.000 | 0.000 | 0.000 | 0.000 | 1.000 | 0.000 | 27.3 | 29 |
| A363 | Yuedao93 | Vietnam | 0.000 | 0.000 | 0.001 | 0.000 | 0.000 | 0.998 | 0.000 | 26.6 | 25.5 |
| **A364** | **Yuedao94** | Vietnam | 0.000 | 0.000 | 0.000 | 0.000 | 0.000 | 0.999 | 0.000 | 35.1 | 34.6 |
| A365 | Yuedao95 | Vietnam | 0.000 | 0.000 | 0.000 | 0.000 | 0.000 | 0.999 | 0.000 | 25.2 | 26.8 |
| **A366** | **Yuedao96** | Vietnam | 0.000 | 0.000 | 0.000 | 0.000 | 0.093 | 0.906 | 0.001 | 34.3 | 31.4 |
| **A367** | **Yuedao97** | Vietnam | 0.000 | 0.000 | 0.000 | 0.000 | 0.000 | 0.999 | 0.000 | 33.3 | 32.1 |
| A368 | Yuedao98 | Vietnam | 0.000 | 0.000 | 0.000 | 0.000 | 0.000 | 1.000 | 0.000 | 29.7 | 29.4 |
| **A369** | **Yuedao99** | Vietnam | 0.000 | 0.000 | 0.000 | 0.000 | 0.000 | 0.999 | 0.000 | 32 | 34.2 |
| A370 | Yuedao100 | Vietnam | 0.000 | 0.000 | 0.000 | 0.000 | 0.000 | 0.999 | 0.000 | 29.3 | 31.3 |
| A371 | Yuedao101 | Vietnam | 0.000 | 0.000 | 0.000 | 0.000 | 0.000 | 1.000 | 0.000 | 23.6 | 26.7 |
| A372 | Yuedao102 | Vietnam | 0.000 | 0.000 | 0.000 | 0.000 | 0.000 | 0.999 | 0.000 | 24.4 | 25.6 |
| A373 | Yuedao103 | Vietnam | 0.000 | 0.001 | 0.000 | 0.000 | 0.003 | 0.988 | 0.007 | 30.1 | 28.3 |
| A374 | Yuedao104 | Vietnam | 0.000 | 0.000 | 0.000 | 0.000 | 0.000 | 0.999 | 0.000 | 28.1 | 28.9 |
| A375 | Yuedao105 | Vietnam | 0.000 | 0.000 | 0.000 | 0.000 | 0.000 | 0.999 | 0.000 | 27.2 | 28.5 |
| A376 | Yuedao106 | Vietnam | 0.000 | 0.000 | 0.000 | 0.000 | 0.000 | 0.999 | 0.000 | 27.6 | 28.3 |
| A377 | Yuedao107 | Vietnam | 0.000 | 0.000 | 0.000 | 0.000 | 0.011 | 0.988 | 0.000 | 25.3 | 28 |
| A378 | Yuedao108 | Vietnam | 0.000 | 0.002 | 0.000 | 0.000 | 0.081 | 0.916 | 0.001 | 29.2 | 29.6 |
| A379 | Yuedao109 | Vietnam | 0.001 | 0.025 | 0.001 | 0.000 | 0.071 | 0.901 | 0.001 | 26.1 | 28.4 |
| A380 | Yuedao110 | Vietnam | 0.000 | 0.000 | 0.000 | 0.000 | 0.000 | 0.999 | 0.000 | 28 | 29.8 |
| A381 | Yuedao111 | Vietnam | 0.000 | 0.000 | 0.000 | 0.000 | 0.000 | 0.999 | 0.000 | 27.1 | 29.3 |
| A382 | Yuedao112 | Vietnam | 0.000 | 0.000 | 0.000 | 0.000 | 0.000 | 0.999 | 0.000 | 28.2 | 27.5 |
| A383 | Yuedao113 | Vietnam | 0.000 | 0.000 | 0.000 | 0.000 | 0.000 | 0.999 | 0.000 | 26 | 26.7 |
| A384 | Yuedao114 | Vietnam | 0.002 | 0.556 | 0.000 | 0.000 | 0.008 | 0.432 | 0.001 | 25.9 | 28.6 |
| A385 | Yuedao115 | Vietnam | 0.000 | 0.603 | 0.000 | 0.000 | 0.011 | 0.386 | 0.000 | 28.8 | 30.9 |
| A386 | Yuedao116 | Vietnam | 0.000 | 0.605 | 0.000 | 0.000 | 0.000 | 0.394 | 0.000 | 27.5 | 29.2 |
| A387 | Yuedao117 | Vietnam | 0.000 | 0.621 | 0.000 | 0.000 | 0.000 | 0.379 | 0.000 | 27.5 | 30.3 |
| **A388** | **Yuedao118** | Vietnam | 0.000 | 0.638 | 0.000 | 0.000 | 0.000 | 0.361 | 0.000 | 32.4 | 31 |
| A389 | Yuedao119 | Vietnam | 0.003 | 0.710 | 0.002 | 0.000 | 0.034 | 0.251 | 0.000 | 22.7 | 24.4 |
| A390 | Yuedao120 | Vietnam | 0.000 | 0.650 | 0.001 | 0.000 | 0.001 | 0.348 | 0.000 | 26.7 | 27.4 |
| A391 | Yuedao121 | Vietnam | 0.000 | 0.668 | 0.000 | 0.000 | 0.003 | 0.327 | 0.001 | 30.2 | 31.9 |
| **A392** | **Jia45** | Jiaxing, Zhejiang | 0.000 | 0.999 | 0.000 | 0.000 | 0.000 | 0.000 | 0.000 | 16.3 | 15.8 |
| A393 | Nannongjing3786 | Nanjing, Jiangsu | 0.000 | 0.998 | 0.000 | 0.000 | 0.000 | 0.001 | 0.000 | 21.1 | 21.5 |
| A394 | 24248 | Nanjing, Jiangsu | 0.000 | 0.999 | 0.000 | 0.000 | 0.000 | 0.000 | 0.000 | 25.7 | 25.6 |
| A395 | Nannongjing4004 | Nanjing, Jiangsu | 0.000 | 0.999 | 0.000 | 0.000 | 0.000 | 0.000 | 0.000 | 22 | 20.7 |
| A396 | Nannongjing4016 | Nanjing, Jiangsu | 0.000 | 0.999 | 0.000 | 0.000 | 0.000 | 0.000 | 0.000 | 21.8 | 21.7 |
| A397 | Zijianwujing | Nanjing, Jiangsu | 0.000 | 0.999 | 0.000 | 0.000 | 0.000 | 0.000 | 0.000 | 17.9 | 17.1 |
| A398 | Ningjing2 | Nanjing, Jiangsu | 0.000 | 0.999 | 0.000 | 0.000 | 0.000 | 0.000 | 0.000 | 21.3 | 20 |
| A399 | Wuxiang99-8 | Wujin, Jiangsu | 0.000 | 0.999 | 0.000 | 0.000 | 0.000 | 0.000 | 0.000 | 19.5 | 20.1 |
| A400 | Wuyujing8 | Wujin, Jiangsu | 0.000 | 0.999 | 0.000 | 0.000 | 0.000 | 0.000 | 0.000 | 18.5 | 18.2 |
| A401 | Nannongjing002 | Nanjing, Jiangsu | 0.000 | 1.000 | 0.000 | 0.000 | 0.000 | 0.000 | 0.000 | 20.2 | 18.9 |
| A402 | Nannongjing004 | Nanjing, Jiangsu | 0.000 | 0.999 | 0.000 | 0.000 | 0.000 | 0.000 | 0.000 | 19.8 | 17.9 |
| **A403** | **Huaidao5hao** | Huaian, Jiangsu | 0.000 | 0.999 | 0.000 | 0.000 | 0.000 | 0.000 | 0.000 | 15.9 | 16 |
| A404 | Zhongzuo93 | Tongzhou, Beijing | 0.000 | 1.000 | 0.000 | 0.000 | 0.000 | 0.000 | 0.000 | 17.9 | 18.5 |
| **A405** | **Yandao9** | Yancheng, Jiangsu | 0.000 | 1.000 | 0.000 | 0.000 | 0.000 | 0.000 | 0.000 | 17.3 | 18.8 |
| **A406** | **Lianjing4** | Lianyungang, Jiangsu | 0.000 | 0.999 | 0.000 | 0.000 | 0.000 | 0.000 | 0.000 | 16.5 | 17.9 |
| A407 | Jindao1007 | Dongli, Tianjin | 0.000 | 0.999 | 0.000 | 0.000 | 0.000 | 0.000 | 0.000 | 19.6 | 19.5 |
| A408 | Huajing5 | Huaibei, Jiangsu | 0.000 | 0.999 | 0.000 | 0.000 | 0.000 | 0.000 | 0.000 | 17.7 | 18.2 |
| **A409** | **Huajing6** | Huaibei, Jiangsu | 0.000 | 1.000 | 0.000 | 0.000 | 0.000 | 0.000 | 0.000 | 17.6 | 17.8 |
| A410 | Yangfujing7 | Lixiahe, Jiangsu | 0.000 | 0.999 | 0.000 | 0.000 | 0.000 | 0.000 | 0.000 | 19.4 | 20.1 |
| A411 | Yangfujing8 | Lixiahe, Jiangsu | 0.000 | 0.999 | 0.000 | 0.000 | 0.000 | 0.000 | 0.000 | 19.8 | 20.2 |
| **A412** | **Zhendao99** | Zhenjiang, Jiangsu | 0.000 | 1.000 | 0.000 | 0.000 | 0.000 | 0.000 | 0.000 | 17.6 | 17.8 |
| A413 | Nanjing42 | Nanjing, Jiangsu | 0.000 | 0.999 | 0.000 | 0.000 | 0.000 | 0.000 | 0.000 | 19.5 | 18.5 |
| **A414** | **Lianjing9823** | Lianyungang, Jiangsu | 0.000 | 1.000 | 0.000 | 0.000 | 0.000 | 0.000 | 0.000 | 17.2 | 18.2 |
| A415 | Huifeng1 | Yancheng, Jiangsu | 0.000 | 1.000 | 0.000 | 0.000 | 0.000 | 0.000 | 0.000 | 17.8 | 17.6 |
| A416 | Huifeng2 | Yancheng, Jiangsu | 0.000 | 1.000 | 0.000 | 0.000 | 0.000 | 0.000 | 0.000 | 17.8 | 18.3 |
| A417 | Yandao8 | Yancheng, Jiangsu | 0.000 | 1.000 | 0.000 | 0.000 | 0.000 | 0.000 | 0.000 | 17.9 | 17.3 |
| **A418** | **Wuyujing21** | Wujin, Jiangsu | 0.000 | 1.000 | 0.000 | 0.000 | 0.000 | 0.000 | 0.000 | 17.5 | 18.7 |
| A419 | Shashani | Haerbin, Heilongjiang | 0.000 | 1.000 | 0.000 | 0.000 | 0.000 | 0.000 | 0.000 | 19.3 | 20.4 |
| **A420** | **Muzhan4** | Mudanjiang, Heilongjiang | 0.000 | 1.000 | 0.000 | 0.000 | 0.000 | 0.000 | 0.000 | 16.5 | 15.7 |
| A421 | Mudanjiang29 | Mudanjiang, Heilongjiang | 0.000 | 1.000 | 0.000 | 0.000 | 0.000 | 0.000 | 0.000 | 19 | 17.3 |
| **A422** | **Mudanjiang28** | Mudanjiang, Heilongjiang | 0.000 | 0.999 | 0.000 | 0.000 | 0.000 | 0.000 | 0.000 | 18.3 | 17.3 |
| A423 | Mudanjiang27 | Mudanjiang, Heilongjiang | 0.000 | 0.999 | 0.000 | 0.000 | 0.000 | 0.001 | 0.000 | 22.8 | 18.2 |
| A424 | Kenzhan2 | Nongken, Heilongjiang | 0.000 | 0.999 | 0.000 | 0.000 | 0.000 | 0.000 | 0.000 | 17.5 | 16.4 |
| **A425** | **Heijing8** | Haerbin, Heilongjiang | 0.000 | 0.999 | 0.000 | 0.000 | 0.000 | 0.000 | 0.000 | 16.8 | 17.5 |
| **A426** | **Hejing1** | Haerbin, Heilongjiang | 0.000 | 0.999 | 0.000 | 0.000 | 0.000 | 0.000 | 0.000 | 15.9 | 16 |
| **A427** | **Beidao4** | Haerbin, Heilongjiang | 0.000 | 0.999 | 0.000 | 0.000 | 0.000 | 0.000 | 0.000 | 27.3 | 26.2 |
| A428 | Beidao3 | Haerbin, Heilongjiang | 0.000 | 0.999 | 0.000 | 0.000 | 0.000 | 0.000 | 0.000 | 18.2 | 16.5 |
| A429 | Suijing12 | Suihua, Heilongjiang | 0.000 | 0.999 | 0.000 | 0.000 | 0.000 | 0.000 | 0.000 | 17.7 | 15.8 |
| A430 | Songjing12 | Songhuajiang, Heilongjiang | 0.000 | 0.999 | 0.000 | 0.000 | 0.000 | 0.000 | 0.000 | 23 | 21.7 |
| A431 | Songjing11 | Songhuajiang, Heilongjiang | 0.000 | 0.995 | 0.000 | 0.000 | 0.002 | 0.002 | 0.001 | 22.4 | 20 |
| **A432** | **Songjing10** | Songhuajiang, Heilongjiang | 0.980 | 0.019 | 0.000 | 0.000 | 0.000 | 0.000 | 0.000 | 17.6 | 15.7 |
| A433 | Dongnong430 | Haerbin, Heilongjiang | 0.999 | 0.000 | 0.000 | 0.000 | 0.000 | 0.000 | 0.000 | 24 | 20.6 |
| A434 | Dongnong424 | Haerbin, Heilongjiang | 0.999 | 0.000 | 0.000 | 0.000 | 0.000 | 0.000 | 0.000 | 19.7 | 17 |
| A435 | Longnuo3 | Haerbin, Heilongjiang | 0.998 | 0.000 | 0.000 | 0.000 | 0.000 | 0.000 | 0.001 | 17.2 | 18.5 |
| **A436** | **Longjing28** | Haerbin, Heilongjiang | 0.999 | 0.000 | 0.000 | 0.000 | 0.000 | 0.000 | 0.000 | 17.5 | 15.4 |
| **A437** | **Longjing27** | Haerbin, Heilongjiang | 0.998 | 0.001 | 0.000 | 0.000 | 0.000 | 0.000 | 0.000 | 16 | 14 |
| A438 | Longjing26 | Haerbin, Heilongjiang | 0.999 | 0.000 | 0.000 | 0.000 | 0.000 | 0.000 | 0.000 | 17.7 | 16.1 |
| **A439** | **Longjing25** | Haerbin, Heilongjiang | 0.999 | 0.000 | 0.000 | 0.000 | 0.000 | 0.000 | 0.000 | 12.6 | 14.3 |
| A440 | Longjing24 | Haerbin, Heilongjiang | 0.997 | 0.002 | 0.000 | 0.000 | 0.000 | 0.000 | 0.000 | 18.9 | 17.5 |
| A441 | Longjing22 | Haerbin, Heilongjiang | 0.999 | 0.001 | 0.000 | 0.000 | 0.000 | 0.000 | 0.000 | 18 | 15.2 |
| A442 | Longjing21 | Haerbin, Heilongjiang | 0.993 | 0.004 | 0.002 | 0.000 | 0.000 | 0.000 | 0.000 | 18.7 | 16.3 |
| **A443** | **Longjing20** | Haerbin, Heilongjiang | 0.998 | 0.002 | 0.000 | 0.000 | 0.000 | 0.000 | 0.000 | 15.7 | 14.8 |
| A444 | Longjing19 | Haerbin, Heilongjiang | 0.995 | 0.004 | 0.000 | 0.000 | 0.000 | 0.000 | 0.000 | 16.9 | 15 |
| A445 | Longjing18 | Haerbin, Heilongjiang | 0.093 | 0.752 | 0.002 | 0.000 | 0.096 | 0.056 | 0.001 | 18.3 | 13.8 |
| A446 | Longjing17 | Haerbin, Heilongjiang | 0.999 | 0.001 | 0.000 | 0.000 | 0.000 | 0.000 | 0.000 | 17.5 | 15.4 |
| **A447** | **Longjing16** | Haerbin, Heilongjiang | 0.999 | 0.001 | 0.000 | 0.000 | 0.000 | 0.000 | 0.000 | 16.7 | 15.4 |
| A448 | Longjing15 | Haerbin, Heilongjiang | 0.999 | 0.001 | 0.000 | 0.000 | 0.000 | 0.000 | 0.000 | 17.4 | 17.8 |
| A449 | Zhonglongdao1 | Haerbin, Heilongjiang | 0.999 | 0.000 | 0.000 | 0.000 | 0.000 | 0.000 | 0.000 | 22.8 | 21.8 |
| **A450** | **Longdao8** | Haerbin, Heilongjiang | 0.999 | 0.000 | 0.000 | 0.000 | 0.000 | 0.000 | 0.000 | 17 | 16.3 |
| A451 | Longdao6 | Haerbin, Heilongjiang | 0.999 | 0.000 | 0.000 | 0.000 | 0.000 | 0.000 | 0.000 | 23.3 | 19.2 |
| A452 | Longdao5 | Haerbin, Heilongjiang | 0.991 | 0.008 | 0.000 | 0.000 | 0.000 | 0.000 | 0.000 | 21.1 | 23.4 |
| **A453** | **Longdao4** | Haerbin, Heilongjiang | 0.999 | 0.000 | 0.000 | 0.000 | 0.000 | 0.000 | 0.000 | 17.5 | 13.8 |
| A454 | Kendao19 | Haerbin, Heilongjiang | 0.989 | 0.001 | 0.008 | 0.000 | 0.000 | 0.001 | 0.001 | 18.3 | 16.6 |
| A455 | Kendao18 | Haerbin, Heilongjiang | 0.999 | 0.000 | 0.000 | 0.000 | 0.000 | 0.000 | 0.000 | 19.1 | 16.2 |
| A456 | Kendao12 | Haerbin, Heilongjiang | 0.085 | 0.741 | 0.002 | 0.000 | 0.096 | 0.076 | 0.000 | 21.3 | 18.8 |
| A457 | Kendao13 | Haerbin, Heilongjiang | 0.999 | 0.000 | 0.000 | 0.000 | 0.000 | 0.000 | 0.000 | 18.3 | 16.5 |
| **A458** | **Kendao20** | Haerbin, Heilongjiang | 0.999 | 0.000 | 0.000 | 0.000 | 0.000 | 0.000 | 0.000 | 15.8 | 17 |
| A459 | Longdun106 | Haerbin, Heilongjiang | 0.999 | 0.000 | 0.000 | 0.000 | 0.000 | 0.000 | 0.000 | 19.2 | 16.1 |
| A460 | Longdun105 | Haerbin, Heilongjiang | 0.999 | 0.000 | 0.000 | 0.000 | 0.000 | 0.000 | 0.000 | 18.6 | 16.2 |
| **A461** | **Sanjiang2** | Haerbin, Heilongjiang | 0.997 | 0.002 | 0.000 | 0.000 | 0.000 | 0.000 | 0.000 | 17 | 15.4 |
| A462 | Nongxiang21 | Changsha, Hunan | 0.000 | 0.000 | 0.000 | 0.998 | 0.001 | 0.000 | 0.000 | 27.9 | 27.5 |
| A463 | Nongxiang25 | Changsha, Hunan | 0.000 | 0.000 | 0.000 | 0.998 | 0.000 | 0.001 | 0.000 | 33.2 | 29.7 |
| A464 | Nongxiang26 | Changsha, Hunan | 0.000 | 0.000 | 0.000 | 0.999 | 0.000 | 0.000 | 0.000 | 30.1 | 28.5 |
| A465 | Yuzhenxiang | Changsha, Hunan | 0.000 | 0.000 | 0.000 | 0.999 | 0.000 | 0.000 | 0.000 | 29.1 | 28 |
| A466 | Xiangwanxian17 | Changsha, Hunan | 0.000 | 0.000 | 0.000 | 0.998 | 0.000 | 0.000 | 0.000 | 27.7 | 27.5 |
| A467 | Huanghuazhan | Changsha, Hunan | 0.000 | 0.000 | 0.000 | 0.974 | 0.000 | 0.026 | 0.000 | 25.5 | 29 |
| A468 | Fengyouwan8 | Changsha, Hunan | 0.000 | 0.000 | 0.000 | 0.999 | 0.000 | 0.000 | 0.000 | 27.9 | 29.9 |
| A469 | Ribenqing | Haerbin, Heilongjiang | 0.000 | 0.000 | 0.000 | 0.999 | 0.000 | 0.000 | 0.000 | 21.3 | 23.8 |
| A470 | Tijin | Haerbin, Heilongjiang | 0.000 | 0.007 | 0.002 | 0.988 | 0.003 | 0.000 | 0.000 | 21.5 | 20.1 |
| A471 | M1004 | Haerbin, Heilongjiang | 0.000 | 0.000 | 0.000 | 0.999 | 0.000 | 0.000 | 0.000 | 21.8 | 22.4 |
| A472 | Zhongguo91 | Haerbin, Heilongjiang | 0.000 | 0.000 | 0.000 | 0.999 | 0.000 | 0.000 | 0.000 | 26.1 | 25.8 |
| A473 | Qiutainxiaoding | Haerbin, Heilongjiang | 0.000 | 0.000 | 0.000 | 0.999 | 0.000 | 0.000 | 0.000 | 23.3 | 22 |
| A474 | Qiuguang | Haerbin, Heilongjiang | 0.000 | 0.000 | 0.000 | 0.999 | 0.000 | 0.000 | 0.000 | 20.8 | 21.9 |
| A475 | Kangbingyueguang | Haerbin, Heilongjiang | 0.000 | 0.000 | 0.000 | 1.000 | 0.000 | 0.000 | 0.000 | 20.3 | 22.2 |
| A476 | Youzhiyueguang | Haerbin, Heilongjiang | 0.000 | 0.000 | 0.000 | 1.000 | 0.000 | 0.000 | 0.000 | 21.6 | 22.5 |
| A477 | Kasala | Haerbin, Heilongjiang | 0.002 | 0.002 | 0.006 | 0.982 | 0.007 | 0.000 | 0.000 | 29.2 | 31.5 |
| A478 | Xiangchuanwuxinbaimi | Haerbin, Heilongjiang | 0.000 | 0.000 | 0.000 | 0.976 | 0.000 | 0.023 | 0.000 | 21.8 | 23.9 |
| A479 | Dongzheng1640 | Hongze, Jiangsu | 0.000 | 0.000 | 0.000 | 0.999 | 0.000 | 0.000 | 0.000 | 18 | 21.6 |
| A480 | Dongzhengwuyujing21 | Hongze, Jiangsu | 0.000 | 0.000 | 0.000 | 1.000 | 0.000 | 0.000 | 0.000 | 16.4 | 19.5 |
| A481 | Heimixiandao | Nanjing, Jiangsu | 0.000 | 0.000 | 0.000 | 0.999 | 0.000 | 0.000 | 0.000 | 22.8 | 22.5 |
| A482 | Heimijingdao | Nanjing, Jiangsu | 0.000 | 0.000 | 0.000 | 0.999 | 0.000 | 0.000 | 0.000 | 20.7 | 21.9 |
| A483 | Zidao | Nanjing, Jiangsu | 0.000 | 0.000 | 0.000 | 0.999 | 0.000 | 0.000 | 0.000 | 23 | 23.8 |
| A484 | 9311 | Yangzhou, Jiangsu | 0.000 | 0.000 | 0.000 | 0.999 | 0.000 | 0.000 | 0.000 | 25.6 | 23.2 |
| A485 | Zacaodao | Nanjing, Jiangsu | 0.000 | 0.000 | 0.000 | 1.000 | 0.000 | 0.000 | 0.000 | 20.4 | 20.2 |
| A486 | Youmang429 | Nanjing, Jiangsu | 0.000 | 0.000 | 0.000 | 1.000 | 0.000 | 0.000 | 0.000 | 23.4 | 25.1 |
| A487 | Wanqu429 | Nanjing, Jiangsu | 0.000 | 0.000 | 0.000 | 1.000 | 0.000 | 0.000 | 0.000 | 24.7 | 27.6 |
| A488 | Zhili429 | Nanjing, Jiangsu | 0.000 | 0.000 | 0.000 | 0.999 | 0.000 | 0.000 | 0.000 | 18.1 | 17.9 |
| A489 | Si4029 | Sihong, Jiangsu | 0.000 | 0.000 | 0.000 | 1.000 | 0.000 | 0.000 | 0.000 | 18.8 | 20.5 |
| A490 | Si4031 | Sihong, Jiangsu | 0.000 | 0.000 | 0.000 | 1.000 | 0.000 | 0.000 | 0.000 | 18.3 | 20 |
| A491 | Si4033 | Sihong, Jiangsu | 0.000 | 0.000 | 0.000 | 1.000 | 0.000 | 0.000 | 0.000 | 22.2 | 20.6 |
| A492 | Si4039 | Sihong, Jiangsu | 0.000 | 0.000 | 0.000 | 1.000 | 0.000 | 0.000 | 0.000 | 19.7 | 20 |
| A493 | Si4040 | Sihong, Jiangsu | 0.000 | 0.000 | 0.000 | 0.999 | 0.000 | 0.000 | 0.000 | 18.1 | 18.2 |
| A494 | Si4041 | Sihong, Jiangsu | 0.000 | 0.000 | 0.000 | 1.000 | 0.000 | 0.000 | 0.000 | 17.5 | 18.9 |
| A495 | Si4049 | Sihong, Jiangsu | 0.000 | 0.000 | 0.000 | 0.999 | 0.000 | 0.000 | 0.000 | 19.3 | 19 |
| A496 | Si4079 | Sihong, Jiangsu | 0.000 | 0.000 | 0.000 | 1.000 | 0.000 | 0.000 | 0.000 | 18.6 | 20.4 |
| A497 | Si4081 | Sihong, Jiangsu | 0.000 | 0.000 | 0.000 | 1.000 | 0.000 | 0.000 | 0.000 | 20.5 | 20.9 |
| A498 | Si4082 | Sihong, Jiangsu | 0.000 | 0.000 | 0.000 | 1.000 | 0.000 | 0.000 | 0.000 | 18.2 | 18.3 |
| A499 | Si4139 | Sihong, Jiangsu | 0.000 | 0.000 | 0.000 | 1.000 | 0.000 | 0.000 | 0.000 | 19.3 | 19.2 |
| A500 | Si4152 | Sihong, Jiangsu | 0.000 | 0.000 | 0.000 | 0.999 | 0.000 | 0.000 | 0.000 | 17.7 | 17.6 |
| A501 | Si4161 | Sihong, Jiangsu | 0.000 | 0.000 | 0.000 | 1.000 | 0.000 | 0.000 | 0.000 | 18.1 | 19.2 |
| A502 | Si4229 | Sihong, Jiangsu | 0.000 | 0.000 | 0.000 | 0.999 | 0.000 | 0.000 | 0.000 | 20 | 20.4 |
| A503 | Si4230 | Sihong, Jiangsu | 0.000 | 0.000 | 0.000 | 1.000 | 0.000 | 0.000 | 0.000 | 18.1 | 20.8 |
| A504 | Si4251 | Sihong, Jiangsu | 0.000 | 0.000 | 0.000 | 1.000 | 0.000 | 0.000 | 0.000 | 19.2 | 18.9 |
| A505 | Si4252 | Sihong, Jiangsu | 0.000 | 0.000 | 0.000 | 1.000 | 0.000 | 0.000 | 0.000 | 18.8 | 19.1 |
| A506 | Si4259 | Sihong, Jiangsu | 0.000 | 0.000 | 0.000 | 1.000 | 0.000 | 0.000 | 0.000 | 23.1 | 26.3 |
| A507 | Si4263 | Sihong, Jiangsu | 0.000 | 0.000 | 0.000 | 1.000 | 0.000 | 0.000 | 0.000 | 19.2 | 19 |
| A508 | Si4280 | Sihong, Jiangsu | 0.000 | 0.000 | 0.000 | 1.000 | 0.000 | 0.000 | 0.000 | 18.2 | 19.5 |
| A509 | Si4330 | Sihong, Jiangsu | 0.000 | 0.000 | 0.002 | 0.998 | 0.000 | 0.000 | 0.000 | 19.8 | 20.2 |
| A510 | Si4360 | Sihong, Jiangsu | 0.000 | 0.000 | 0.000 | 0.999 | 0.000 | 0.000 | 0.000 | 20.4 | 21.5 |
| A511 | Si4364 | Sihong, Jiangsu | 0.000 | 0.000 | 0.000 | 0.999 | 0.000 | 0.000 | 0.000 | 18.3 | 18.4 |
| A512 | Digudao | Sihong, Jiangsu | 0.000 | 0.000 | 0.000 | 0.999 | 0.000 | 0.000 | 0.000 | 18.2 | 21.5 |
| A513 | Si4385 | Sihong, Jiangsu | 0.000 | 0.000 | 0.000 | 0.999 | 0.000 | 0.000 | 0.000 | 22.7 | 23.2 |
| A514 | Si4386 | Sihong, Jiangsu | 0.000 | 0.000 | 0.000 | 0.997 | 0.000 | 0.002 | 0.000 | 24.9 | 25.3 |
| A515 | Sihao4141 | Sihong, Jiangsu | 0.000 | 0.000 | 0.000 | 0.999 | 0.000 | 0.000 | 0.000 | 17.7 | 19.2 |
| A516 | Ningjinghui096 | Nanjing, Jiangsu | 0.000 | 0.000 | 0.000 | 0.999 | 0.000 | 0.000 | 0.000 | 17.9 | 16.7 |
| A517 | Ningjinghui117 | Nanjing, Jiangsu | 0.000 | 0.000 | 0.000 | 0.999 | 0.000 | 0.000 | 0.000 | 19.4 | 20.6 |
| A518 | Ningjinghui145 | Nanjing, Jiangsu | 0.000 | 0.000 | 0.000 | 0.999 | 0.000 | 0.000 | 0.000 | 24.1 | 24.5 |
| A519 | Ningjinghui166 | Nanjing, Jiangsu | 0.000 | 0.000 | 0.000 | 0.999 | 0.000 | 0.000 | 0.000 | 16.9 | 18.2 |
| A520 | Ningjinghui208 | Nanjing, Jiangsu | 0.000 | 0.000 | 0.000 | 0.999 | 0.000 | 0.000 | 0.000 | 17.9 | 17 |
| A521 | Ningjinghui210 | Nanjing, Jiangsu | 0.000 | 0.000 | 0.000 | 0.999 | 0.000 | 0.000 | 0.000 | 19.2 | 18.7 |
| A522 | Ningjinghui237 | Nanjing, Jiangsu | 0.000 | 0.000 | 0.000 | 0.999 | 0.000 | 0.000 | 0.000 | 21.2 | 21.3 |
| A523 | Ningjinghui246 | Nanjing, Jiangsu | 0.000 | 0.000 | 0.000 | 0.999 | 0.000 | 0.000 | 0.000 | 18.7 | 20.4 |
| A524 | Ningjinghui260 | Nanjing, Jiangsu | 0.000 | 0.000 | 0.000 | 0.999 | 0.000 | 0.000 | 0.000 | 20.1 | 20.3 |
| A525 | Ningjinghui285 | Nanjing, Jiangsu | 0.000 | 0.000 | 0.000 | 0.999 | 0.000 | 0.000 | 0.000 | 18.7 | 19.9 |
| A526 | Ningjinghui286 | Nanjing, Jiangsu | 0.000 | 0.000 | 0.000 | 0.999 | 0.000 | 0.000 | 0.000 | 18.1 | 17.9 |
| A527 | Ningjinghui290 | Nanjing, Jiangsu | 0.000 | 0.000 | 0.000 | 0.999 | 0.000 | 0.000 | 0.000 | 16.8 | 16.4 |
| A528 | Ningjinghui292 | Nanjing, Jiangsu | 0.000 | 0.000 | 0.000 | 0.999 | 0.000 | 0.000 | 0.000 | 17.7 | 17.3 |
| A529 | Ningjinghui293 | Nanjing, Jiangsu | 0.000 | 0.000 | 0.000 | 0.999 | 0.000 | 0.000 | 0.000 | 17.9 | 20 |
| A530 | Ningjinghui296 | Nanjing, Jiangsu | 0.000 | 0.000 | 0.000 | 0.999 | 0.000 | 0.000 | 0.000 | 18.7 | 18.5 |
| A531 | Ningjinghui298 | Nanjing, Jiangsu | 0.000 | 0.000 | 0.000 | 1.000 | 0.000 | 0.000 | 0.000 | 19.8 | 19.1 |
| A532 | Ningjinghui338 | Nanjing, Jiangsu | 0.000 | 0.000 | 0.000 | 0.999 | 0.000 | 0.000 | 0.000 | 20.2 | 20 |
| A533 | Hongyin1009 | Nanjing, Jiangsu | 0.000 | 0.000 | 0.000 | 0.999 | 0.000 | 0.000 | 0.000 | 16.9 | 18.3 |
| A534 | Hongyin1010 | Nanjing, Jiangsu | 0.000 | 0.000 | 0.000 | 0.999 | 0.000 | 0.000 | 0.000 | 19.9 | 20 |
| A535 | Hongyin1011 | Nanjing, Jiangsu | 0.000 | 0.000 | 0.000 | 0.999 | 0.000 | 0.000 | 0.000 | 18.5 | 20.3 |
| A536 | Hongyin1012 | Nanjing, Jiangsu | 0.000 | 0.000 | 0.000 | 0.999 | 0.000 | 0.000 | 0.000 | 18.1 | 17.7 |
| A537 | Cai | Haerbin, Heilongjiang | 0.000 | 0.000 | 0.000 | 0.999 | 0.000 | 0.000 | 0.000 | 21.2 | 22.9 |
| A538 | Nannongjing1R | Nanjing, Jiangsu | 0.000 | 0.000 | 0.000 | 0.999 | 0.000 | 0.000 | 0.000 | 29.4 | 30.7 |
| A539 | Nannongjing2R | Nanjing, Jiangsu | 0.000 | 0.000 | 0.000 | 0.999 | 0.000 | 0.000 | 0.000 | 27.7 | 28.3 |
| A540 | Nannongjing3R | Nanjing, Jiangsu | 0.000 | 0.000 | 0.000 | 0.999 | 0.000 | 0.000 | 0.000 | 19.6 | 20.8 |
| Bold and underline accession names are the varieties selected for sequencing analysis of *LP1.* | | | | | | | | | | | |
