# Supplementary material for: Identification of a Candidate Gene for Panicle Length in Rice (Oryza sativa L.) Via Association and Linkage Analysis
Source: Front Plant Sci. 2016 May 3;7:596. doi: 10.3389/fpls.2016.00596 (PMC4853638; doi:10.3389/fpls.2016.00596)
Supplement: Supplementary Table 2 — Primer sequences designed in this study. [file Table2.DOCX]

**Supplementary Table 2.** Primer sequences designed in this study.

| **Marker** | **Marker Type/Gene ID** | | **Purpose** | **Forward primer (5’-3’)** | **Reverse primer (5’-3’)** | **Length of the product** |
| --- | --- | --- | --- | --- | --- | --- |
| **RM5652** | | SSR | Fine mapping | GTCCTGAGTGACTCGATAGCG | CACGCGTACGTAGCAGAGAG | 200 |
| **RM3700** | | SSR | Fine mapping | AAATGCCCCATGCACAAC | TTGTCAGATTGTCACCAGGG | 142 |
| **RM24428** | | SSR | Fine mapping | CTTACTTAGCACGTGCCTGTTGC | CATTGGGATCATGCATCTCTGC | 323 |
| **RM24457** | | SSR | Fine mapping | CCTTTATGCATGTGTGGAACAGC | GTAATGCCAAAGGGAATGTGAGG | 161 |
| **RM3600** | | SSR | Fine mapping | TGCCCACACATGATGAGC | AACGGGCAAGAGATCTTCTG | 91 |
| **RM7289** | | SSR | Fine mapping | GGCCCACGACTTAATAGACATCG | ACAGCGAACGTGGTGTCTCC | 193 |
| **RM24489** | | SSR | Fine mapping | CTAGATGGTTATCTCTCACGTTGC | CTAGGCCTAATTGACACTTTCAGG | 94 |
| **RM24496** | | SSR | Fine mapping | AGCCGAACATGCCCAAAGAAGC | TGGTATGGCCCAAAGAATAGGAACG | 182 |
| **RM410** | | SSR | Fine mapping | GCTCAACGTTTCGTTCCTG | GAAGATGCGTAAAGTGAACGG | 183 |
| **OSR28** | | SSR | Fine mapping | AGCAGCTATAGCTTAGCTGG | ACTGCACATGAGCAGAGACA | 179 |
| **L12** | | InDel | Fine mapping | CTCCAGTTCTCCACCCACCC | CTGAGCTCTCTCGGGTGTC | 167 |
| **L04** | | InDel | Fine mapping | GGACCAACAACATCACAAC | TACCACCATCACCACCTT | 126 |
| **qRT1** | | *Os09g0456100* | Real time RT-PCR | CCAGCCTCTCTGGACTTTGG | ACCTTTGGAAGCAAGCGGTA | 234 |
| **qRT2** | | *Os09g0456200* | Real time RT-PCR | GACCACTGCCTTCTTGGGAA | GGTCGCTGTTCCCCTTATCG | 203 |
| **qRT3** | | *Os09g0456700* | Real time RT-PCR | GCACCGGTCCGTTTTTAGGA | GCTCTGGGTCGACAACTACC | 181 |
| **qRT4** | | *Os09g0456800* | Real time RT-PCR | CGCCTCAGTCCTCCAAATCC | TCCATCGTCAGCGTTTGGTT | 199 |
| **qRT5** | | *Os09g0456900* | Real time RT-PCR | ACCAGGCCAAGATGATACAGA | CGCTAATCAGTAAGCTTGAGCC | 118 |
| **qRT6** | | *Os09g0457100* | Real time RT-PCR | CGAAGAAGACGTTGGGGTCT | TCATCTCCTTCTTCATTTCTCTTGC | 180 |
| **qLP1** | | *Os09g0456100* | Cloning | GTAGTCCTGCTACTGCAGCG | TGCCATCTATATTTGTGAACGAGA | 5646 |
