# Supplementary material for: Identification of a Candidate Gene for Panicle Length in Rice (Oryza sativa L.) Via Association and Linkage Analysis
Source: Front Plant Sci. 2016 May 3;7:596. doi: 10.3389/fpls.2016.00596 (PMC4853638; doi:10.3389/fpls.2016.00596)
Supplement: Supplementary Table 3 — Descriptive statistics for panicle length (cm) in 540 rice accessions in 2011 and 2012. [file Table3.DOCX]

**Supplementary Table 3.** Descriptive statistics for panicle length (cm) in 540 rice accessions in 2011 and 2012.

| **Year** | **2011** | **2012** |
| --- | --- | --- |
| **Mean** | 23.42 | 23.68 |
| **Minimum** | 11.90 | 12.10 |
| **Maximum** | 39.98 | 39.82 |
| **SD** | 4.56 | 4.73 |
| **CV (%)** | 19.45 | 19.95 |
| ***H^2^*_B_** | 96.38 | 96.80 |
