# Supplementary material for: Identification of a Candidate Gene for Panicle Length in Rice (Oryza sativa L.) Via Association and Linkage Analysis
Source: Front Plant Sci. 2016 May 3;7:596. doi: 10.3389/fpls.2016.00596 (PMC4853638; doi:10.3389/fpls.2016.00596)
Supplement: Supplementary Table 4 — Phenotypic effects of top 10 elite alleles at loci significantly associated with panicle length and their carrier variety. [file Table4.DOCX]

**Supplementary Table 4.** Phenotypic effects of top ten elite alleles at loci significantly associated with panicle length and their carrier variety.

| **Locus-allele** | **Phenotypic effect value** | | | **Carrier variety** |
| --- | --- | --- | --- | --- |
|  | **2011** | **2012** | **Average** |  |
| **RM3600-130** | +8.28 | +9.09 | +8.69 | Yue33 |
| **RM3600-140** | +7.07 | +7.37 | +7.22 | Yue40 |
| **RM3600-125** | +6.94 | +7.32 | +7.13 | Yue64 |
| **RM480-150** | +6.56 | +6.96 | +6.76 | Yue45 |
| **RM276-80** | +6.55 | +6.84 | +6.70 | Yue45 |
| **RM535-285** | +6.38 | +6.78 | +6.58 | Yue28 |
| **RM480-170** | +6.32 | +6.81 | +6.56 | Yue28 |
| **RM535-300** | +6.40 | +6.47 | +6.43 | Yue30 |
| **RM276-110** | +6.07 | +6.02 | +6.04 | Yue94 |
| **RM286-175** | +5.92 | +6.03 | +5.98 | Haonuopie |
