# Supplementary material for: Identification of a Candidate Gene for Panicle Length in Rice (Oryza sativa L.) Via Association and Linkage Analysis
Source: Front Plant Sci. 2016 May 3;7:596. doi: 10.3389/fpls.2016.00596 (PMC4853638; doi:10.3389/fpls.2016.00596)
Supplement: Supplementary Table 5 — Six annotated genes and their putative functions in a 90-kb region of the rice genome. [file Table5.DOCX]

**Supplementary Table 5.** Six annotated genes and their putative functions in a 90-kb region of the rice genome

| **No.** | **Gene name** | **Position (bp)** | **Putative function** |
| --- | --- | --- | --- |
| **1** | [*LOC_Os09g28300*](http://rice.plantbiology.msu.edu/cgi-bin/ORF_infopage.cgi?orf=LOC_Os09g28300) | 17836578-17842026 | Remorin, C-terminal region domain containing protein. |
| **2** | [*LOC_Os09g28310*](http://rice.plantbiology.msu.edu/cgi-bin/ORF_infopage.cgi?orf=LOC_Os09g28300) | 17843808-17847721 | Similar to BZIP transcription factor ABI5 |
| **3** | [*LOC_Os09g28340*](http://rice.plantbiology.msu.edu/cgi-bin/ORF_infopage.cgi?orf=LOC_Os09g28300) | 17861197-17862663 | Conserved hypothetical protein. |
| **4** | [*LOC_Os09g28354*](http://rice.plantbiology.msu.edu/cgi-bin/ORF_infopage.cgi?orf=LOC_Os09g28300) | 17875190-17882634 | Similar to Heat stress transcription factor Spl7 |
| **5** | [*LOC_Os09g28370*](http://rice.plantbiology.msu.edu/cgi-bin/ORF_infopage.cgi?orf=LOC_Os09g28300) | 17888437-17898487 | Nucleic acid-binding, OB-fold domain containing protein. |
| **6** | [*LOC_Os09g28390*](http://rice.plantbiology.msu.edu/cgi-bin/ORF_infopage.cgi?orf=LOC_Os09g28300) | 17905988-17909042 | Cytochrome P450 family protein |
