# Supplementary material for: Identification of a Candidate Gene for Panicle Length in Rice (Oryza sativa L.) Via Association and Linkage Analysis
Source: Front Plant Sci. 2016 May 3;7:596. doi: 10.3389/fpls.2016.00596 (PMC4853638; doi:10.3389/fpls.2016.00596)
Supplement: Supplementary Table 6 — The SNPs detected at the 5.4-kb region of LP1 among two parents and 103 rice accessions. [file Table6.DOCX]

| **Supplementary Table6.** The SNPs detected at the 5.4-kb rengon of *LP1* among two parents and 103 rice accessions. | | | | | | | | | | | | | | | | | | | | | | | | | |
| --- | --- | --- | --- | --- | --- | --- | --- | --- | --- | --- | --- | --- | --- | --- | --- | --- | --- | --- | --- | --- | --- | --- | --- | --- | --- |
| **No.** | **Accessions** | **SNP1** | **SNP3** | **SNP4** | **SNP5** | **SNP6** | **SNP7** | **SNP8** | **SNP9** | **SNP10** | **SNP11** | **SNP12** | **SNP13** | **SNP14** | **SNP15** | **SNP16** | **SNP17** | **SNP18** | **SNP19** | **SNP2** | **SNP20** | **SNP21** | **SNP22** | Panicle length (cm) | |
|  |  |  |  |  |  |  |  |  |  |  |  |  |  |  |  |  |  |  |  |  |  |  |  | 2011 | 2012 |
| **P1** | Xiushui79 | C | G | G | G | C | – | – | G | G | T | – | C | – | A | G | G | – | A | G | A | G | G | 15.53 | 15.61 |
| **P2** | C-bao | T | G | G | G | T | – | – | G | G | T | – | T | – | A | G | G | – | A | T | A | T | G | 26.92 | 26.34 |
| **1** | Longjing25 | C | G | G | G | C | – | – | G | G | T | – | C | – | A | G | G | – | A | G | A | G | G | 11.9 | 12.1 |
| **2** | Toudengyishixing | C | G | G | G | C | – | – | G | G | T | – | C | – | A | G | G | – | A | G | G | G | G | 13.73 | 13.9 |
| **3** | Nannongjing003 | C | G | G | G | C | – | – | G | G | T | – | C | – | A | G | G | – | A | G | A | G | G | 14.53 | 14.7 |
| **4** | Manbaidao | C | G | G | G | C | – | – | G | G | T | – | C | – | A | G | G | – | A | G | A | G | G | 14.7 | 15.05 |
| **5** | Huaidao5 | C | G | G | G | C | – | – | G | G | T | – | C | – | A | G | G | – | A | G | A | G | G | 15.5 | 15.62 |
| **6** | Longjing27 | C | G | G | G | C | – | – | G | G | T | – | C | – | A | G | G | – | A | G | A | G | G | 15.5 | 15.64 |
| **7** | Hejing1 | C | G | T | A | C | – | – | G | G | T | – | C | – | A | G | G | – | A | G | A | G | G | 15.53 | 15.54 |
| **8** | Longjing16 | C | A | G | G | C | – | – | T | G | T | – | C | – | A | G | G | – | A | T | A | G | G | 15.7 | 15.69 |
| **9** | Longjing20 | C | G | G | G | C | – | – | G | G | T | – | C | – | A | G | G | – | A | G | A | G | G | 15.9 | 16.08 |
| **10** | Wanjingnuo | C | G | G | G | C | – | – | G | G | T | – | C | – | A | G | G | – | A | G | A | G | G | 16.07 | 16.16 |
| **11** | Jia45 | C | G | G | G | C | – | – | G | G | T | – | C | – | A | G | G | – | A | T | A | G | G | 16.1 | 16.52 |
| **12** | Tongjing109 | C | G | G | G | C | – | – | G | G | T | – | C | – | A | G | G | – | A | G | A | G | G | 16.13 | 16.21 |
| **13** | Zaoxiaobaidao | C | G | G | G | C | – | – | G | G | T | – | C | – | A | G | G | – | A | G | A | G | G | 16.23 | 16.3 |
| **14** | Jia159 | C | G | G | G | C | – | – | G | G | T | – | C | – | A | G | G | – | A | G | A | G | G | 16.33 | 16.41 |
| **15** | Guihuahuang | C | G | G | G | C | – | – | G | G | T | – | C | – | A | G | G | – | – | T | A | G | G | 16.37 | 16.42 |
| **16** | Zijing | C | G | G | G | C | – | – | G | G | T | – | C | – | A | G | G | – | A | G | A | G | G | 16.43 | 16.49 |
| **17** | Datougui | C | G | G | G | C | – | – | G | G | T | – | C | – | A | G | G | – | A | G | A | G | G | 16.43 | 16.47 |
| **18** | Sanjiang2 | C | G | T | A | C | – | – | T | G | T | – | C | – | A | G | G | – | A | G | A | G | G | 16.43 | 16.45 |
| **19** | Yujing6 | C | G | G | G | C | – | – | G | G | T | – | C | G | A | G | G | – | A | G | A | G | G | 16.53 | 16.57 |
| **20** | Heijing8 | C | G | T | A | C | – | – | G | G | T | – | C | – | A | G | G | – | A | G | A | G | G | 16.53 | 16.87 |
| **21** | Wuqiang | C | G | G | G | C | – | – | G | G | T | – | C | – | A | G | G | G | – | T | A | G | G | 16.57 | 16.64 |
| **22** | Lianjing4 | C | G | G | G | C | – | – | G | G | T | – | C | – | A | G | G | – | A | T | A | G | G | 16.6 | 17.22 |
| **23** | Muzhan4 | C | A | G | G | C | – | – | T | G | T | – | C | – | A | G | G | – | A | T | A | G | G | 16.63 | 17.3 |
| **24** | Yandao9 | C | G | G | G | C | – | – | G | G | T | – | C | – | A | G | G | – | A | T | A | G | G | 16.67 | 17.29 |
| **25** | Wuyujing21 | C | G | G | G | C | – | G | G | G | T | – | C | – | A | G | G | – | A | T | A | G | G | 16.73 | 17.31 |
| **26** | Mudanjiang28 | C | G | G | G | C | – | – | G | G | T | – | C | – | A | G | G | – | A | T | A | G | G | 16.8 | 17.26 |
| **27** | Longdao4 | C | G | G | G | C | – | – | G | G | T | – | C | – | A | G | G | – | A | G | A | G | G | 16.8 | 17.49 |
| **28** | Huajing6 | C | G | G | G | C | – | – | G | G | T | – | C | – | A | G | G | – | A | G | A | G | G | 16.83 | 17.58 |
| **29** | Kendao20 | C | G | G | G | C | – | – | G | G | T | – | C | – | A | G | G | – | A | G | A | G | G | 16.83 | 17.32 |
| **30** | Longjing28 | C | G | G | G | C | – | – | G | G | T | – | C | – | A | G | G | – | A | T | A | G | G | 16.87 | 17.49 |
| **31** | Dongnongjing424 | C | G | T | A | C | – | – | G | G | T | – | C | – | A | G | G | – | A | T | A | G | G | 16.93 | 16.98 |
| **32** | Lianjing9823 | C | G | G | G | C | – | – | G | G | T | – | C | – | A | G | G | – | A | T | A | G | G | 17.03 | 17.15 |
| **33** | Songjing10 | C | G | T | A | C | – | – | G | G | G | – | C | – | A | G | G | – | A | T | A | G | G | 17.07 | 17.27 |
| **34** | Longdao8 | C | G | G | G | C | – | – | G | G | T | – | C | – | A | G | G | – | A | G | A | G | G | 17.07 | 17.22 |
| **35** | Zhendao99 | C | G | G | G | C | – | – | G | G | T | – | C | – | A | G | G | – | A | T | A | G | G | 17.12 | 17.19 |
| **36** | Xiaoluohanhuang | C | G | G | G | C | – | – | G | G | T | – | C | – | A | G | G | – | A | T | A | G | G | 25.8 | 27.01 |
| **37** | Gaoliangqing | C | G | G | G | C | – | – | G | G | T | – | C | – | A | G | G | – | A | T | A | G | G | 25.8 | 26.47 |
| **38** | Jijiaohong | C | A | T | A | C | – | – | T | G | T | – | C | – | A | G | G | – | A | T | A | G | G | 25.83 | 26.16 |
| **39** | Baoxintaihuqing | C | G | T | A | C | – | – | G | G | T | – | C | – | A | G | G | – | A | T | A | G | G | 25.87 | 26.13 |
| **40** | Laowusi | C | G | G | G | C | – | – | G | G | T | – | C | – | A | G | G | – | A | T | A | G | G | 26.07 | 26.16 |
| **41** | Chuyanghan32 | C | G | G | G | C | – | – | G | G | T | – | C | – | A | G | G | – | A | T | A | G | G | 26.07 | 26.27 |
| **42** | Diantun502xuanzao | C | G | G | G | C | – | – | G | G | T | – | C | – | A | G | G | – | A | T | A | G | G | 26.33 | 26.55 |
| **43** | Kuihuanuo | C | G | G | G | C | – | – | G | G | T | – | C | – | A | G | G | – | A | T | A | G | G | 26.33 | 26.61 |
| **44** | Hongmangshajing | C | G | T | A | C | – | – | G | G | T | – | C | – | A | G | G | – | A | T | A | G | G | 26.63 | 26.69 |
| **45** | Jiaobaiyeqing | C | G | T | A | C | – | – | G | G | T | – | C | – | A | G | G | – | A | T | A | G | G | 26.67 | 26.7 |
| **46** | Lamujia | C | G | T | A | C | – | – | G | G | T | – | C | – | A | G | G | – | A | T | A | G | G | 26.7 | 26.82 |
| **47** | Gaidaoqing | C | G | G | G | C | – | – | G | G | T | – | C | – | A | G | G | – | A | T | A | G | G | 26.77 | 27.4 |
| **48** | Wuqitou | C | G | G | G | C | – | – | G | G | T | – | C | – | A | G | G | – | A | T | A | G | G | 26.9 | 27.72 |
| **49** | Kongqueqing | C | G | G | G | C | – | – | G | G | T | – | C | – | A | G | G | – | A | T | A | G | G | 26.97 | 27.95 |
| **50** | Xiganggu | C | G | T | A | C | – | – | G | G | T | – | C | – | A | G | G | – | A | T | A | G | G | 27.03 | 27.19 |
| **51** | Souzhouqing | C | G | G | G | C | – | – | G | G | T | – | C | – | A | G | G | – | A | T | A | G | G | 27.07 | 27.23 |
| **52** | Sujing353 | C | G | G | G | C | – | – | G | G | T | – | C | – | A | G | G | – | A | T | A | G | G | 27.1 | 27.75 |
| **53** | Xiaomandao | C | G | G | G | C | – | – | G | G | T | – | C | – | A | G | G | – | A | G | A | G | G | 27.17 | 27.32 |
| **54** | Lujingqing | C | G | G | G | C | – | – | G | G | T | – | C | – | A | G | G | – | A | G | A | T | C | 27.2 | 27.34 |
| **55** | Yazihuang | T | G | G | G | C | – | – | G | A | T | – | C | – | A | G | G | – | A | G | A | G | G | 27.23 | 27.32 |
| **56** | Yuedao64 | C | G | T | A | C | – | – | G | G | T | – | C | – | A | G | G | – | A | G | A | G | G | 27.23 | 27.37 |
| **57** | Nantouzhong | C | A | G | G | C | – | – | T | G | T | – | C | – | A | G | G | – | A | G | A | G | G | 27.27 | 27.43 |
| **58** | Baigedao | C | G | T | A | C | – | – | G | G | T | – | C | – | A | G | G | – | A | T | A | G | G | 27.33 | 27.5 |
| **59** | Yuedao73 | C | G | T | A | C | – | – | G | G | T | – | C | – | A | G | G | – | A | T | A | G | G | 27.37 | 27.39 |
| **60** | Wanmandao | C | G | G | G | C | – | – | G | G | T | – | C | – | A | G | G | – | A | T | A | G | G | 27.4 | 27.65 |
| **61** | Xianhui429 | C | G | G | G | C | – | – | G | G | T | – | C | – | A | G | G | – | A | T | A | G | G | 27.4 | 27.46 |
| **62** | Wangjiadao | C | G | G | G | C | – | – | G | G | T | – | C | – | A | G | G | – | A | T | A | G | G | 27.53 | 27.61 |
| **63** | Yuedao63 | C | A | G | G | C | – | – | T | G | T | – | C | – | A | G | G | – | A | T | A | G | G | 27.53 | 28.01 |
| **64** | Fenghaungdao | C | G | G | G | C | – | – | G | G | T | – | C | – | A | G | G | – | A | T | A | G | G | 27.57 | 27.98 |
| **65** | Yuedao18 | C | G | G | G | C | – | – | G | G | T | T | C | – | A | G | ? | – | A | G | A | G | G | 27.57 | 27.99 |
| **66** | Beidao4 | C | G | T | A | C | – | – | G | G | T | – | T | – | A | G | G | – | A | T | A | G | G | 27.93 | 28.5 |
| **67** | Baikenuo | C | A | G | A | C | – | – | T | G | T | – | C | – | A | G | G | – | A | T | A | G | G | 27.97 | 28.1 |
| **68** | Fuxiang1 | C | A | G | G | C | G | – | T | G | T | T | C | – | A | G | G | – | A | T | A | G | G | 28 | 28.14 |
| **69** | Yuedao46 | C | G | T | A | C | – | – | G | G | T | – | C | – | A | G | G | – | A | T | A | G | G | 28 | 28.42 |
| **70** | Yuedao75 | C | A | G | G | C | – | – | T | G | T | – | C | – | A | G | G | – | A | T | A | G | G | 28.03 | 28.01 |
| **71** | Luohanhuang | C | G | G | G | C | – | – | G | G | T | – | C | – | A | G | G | – | A | T | A | G | G | 28.07 | 28.08 |
| **72** | Guanchanuo | C | G | T | A | C | – | – | G | G | T | – | C | – | A | G | G | – | A | T | A | G | G | 28.1 | 28.24 |
| **73** | Qiaobinghuang | C | G | T | A | C | – | – | G | G | T | – | C | – | A | G | G | – | A | T | A | G | G | 28.17 | 28.36 |
| **74** | Aiguodadaotou | C | G | T | A | C | – | – | G | G | T | – | C | – | A | G | G | – | A | T | A | G | G | 28.3 | 28.29 |
| **75** | Erheidao | C | G | G | G | C | – | – | G | G | T | – | C | – | A | G | G | – | A | G | A | G | G | 28.47 | 28.45 |
| **76** | Xiaoqingzhong | C | A | G | G | C | – | – | T | G | T | – | C | – | A | G | G | – | A | T | A | G | G | 29 | 29.15 |
| **77** | Daniaodao | C | G | G | G | C | – | – | G | G | T | – | C | – | A | G | G | – | A | T | A | G | G | 29.3 | 30.15 |
| **78** | Buxienuo | C | A | G | G | C | – | – | T | G | T | – | C | – | A | G | G | – | A | T | A | G | G | 29.53 | 30.11 |
| **79** | Qijiangqing | C | G | G | G | C | – | – | G | G | T | – | C | – | A | G | G | – | A | T | A | G | G | 29.77 | 30 |
| **80** | Zijianxian3 | C | A | G | G | C | – | – | T | G | T | – | C | – | – | G | G | – | A | T | A | G | G | 30.2 | 31.06 |
| **81** | Liyangxiaohongdao | C | G | G | G | C | – | – | G | G | T | – | C | – | A | G | G | – | A | T | A | G | G | 30.27 | 31.6 |
| **82** | Yuedao62 | C | A | G | G | C | G | – | T | G | T | – | C | – | A | A | G | – | A | T | A | G | G | 30.4 | 31.76 |
| **83** | Hongdao35 | C | A | G | G | C | – | – | T | G | T | – | C | – | A | G | G | – | A | T | A | G | G | 30.4 | 30.68 |
| **84** | Wumangzaodao | T | G | G | G | T | – | – | G | G | T | – | T | – | A | G | G | – | A | T | A | G | G | 30.63 | 30.81 |
| **85** | Shanhonggu | T | G | G | G | T | – | – | G | G | T | – | T | – | A | G | G | – | – | T | A | G | G | 30.9 | 31.89 |
| **86** | Hongjiaozhan | C | G | T | A | C | – | – | G | G | T | – | T | – | A | G | G | – | A | T | A | G | G | 31.33 | 32.08 |
| **87** | C418 | C | A | G | G | C | – | – | T | G | T | – | C | – | A | G | G | – | A | T | A | G | G | 32.5 | 33.42 |
| **88** | Yuedao65 | C | G | T | A | C | – | – | G | G | T | – | C | – | A | G | G | – | A | T | A | G | G | 32.73 | 32.72 |
| **89** | Yuedao99 | C | G | T | A | C | – | – | G | G | T | – | C | – | A | G | G | – | A | T | A | G | G | 32.73 | 32.97 |
| **90** | Yuedao33 | C | G | T | A | C | – | – | G | G | T | – | C | – | A | G | G | – | A | T | A | G | G | 33.17 | 33.46 |
| **91** | Yuedao27 | C | G | T | A | C | – | – | G | G | T | – | C | – | A | G | ? | – | A | T | A | G | G | 33.33 | 33.42 |
| **92** | Yuedao25 | C | G | T | A | C | – | – | G | G | T | – | C | – | A | G | G | – | A | T | A | G | G | 33.53 | 33.61 |
| **93** | Yuedao97 | C | G | T | A | C | – | – | G | G | T | – | C | – | A | G | G | – | A | T | A | G | G | 33.67 | 34.13 |
| **94** | Yuedao118 | T | G | T | A | C | – | – | G | G | T | – | C | – | A | G | G | – | A | T | A | G | G | 33.7 | 34.15 |
| **95** | Jinghui418 | C | G | G | G | C | – | – | G | G | T | – | C | – | A | G | G | – | A | G | A | G | G | 33.93 | 34.3 |
| **96** | Yuedao45 | C | G | T | A | C | – | – | G | G | T | – | C | – | A | G | G | – | A | G | A | G | G | 34.13 | 34.28 |
| **97** | Yuedao96 | T | G | G | G | T | – | – | G | G | T | – | T | – | A | G | G | – | A | T | A | G | G | 34.53 | 35.19 |
| **98** | Zhendao10 | C | A | G | G | C | – | – | T | G | T | – | C | – | A | G | G | – | A | T | A | G | G | 35 | 35.44 |
| **99** | Yuedao94 | C | G | T | A | C | – | – | G | G | T | – | C | – | A | G | G | – | A | T | A | G | G | 35.4 | 35.91 |
| **100** | Yuedao87 | C | G | T | A | C | – | – | G | G | T | – | C | – | A | G | G | – | A | T | A | G | G | 35.83 | 36.2 |
| **101** | Yuedao86 | T | G | T | A | C | – | – | G | G | T | – | C | – | A | G | G | – | A | G | A | G | G | 36.83 | 37.64 |
| **102** | Haonuopie | T | G | G | G | C | – | – | G | G | T | – | C | – | A | G | G | – | A | T | A | G | G | 39.98 | 39.87 |
| **103** | Nipponbare | C | G | G | G | C | – | – | G | G | T | – | C | – | A | G | G | – | A | G | A | G | G | 15.76 | 15.88 |
