# Supplementary material for: Identification of a Candidate Gene for Panicle Length in Rice (Oryza sativa L.) Via Association and Linkage Analysis
Source: Front Plant Sci. 2016 May 3;7:596. doi: 10.3389/fpls.2016.00596 (PMC4853638; doi:10.3389/fpls.2016.00596)
Supplement: Supplementary Table 7 — Association analysis of the polymorphic markers in LP1 with panicle length. [file Table7.DOCX]

**Supplementary Table 7.** Association analysis of the polymorphic markers in *LP1* with panicle length

| **Marker** | **Cluster report** | **Position(bp)** | **2011** | | **2012** | |
| --- | --- | --- | --- | --- | --- | --- |
|  |  |  | ***P*-value** | **PVE(%）** | ***P*-value** | **PVE(%）** |
| **SNP1** | rs352361290 | 17,837,893 | 0.0011** | 6.23 | 0.0012** | 6.06 |
| **SNP3** | rs21567129 | 17,838,237 | 0.4779 | 0.31 | 0.4558 | 0.34 |
| **SNP4** | rs350830475 | 17,838,294 | 0.1388 | 1.33 | 0.1675 | 1.15 |
| **SNP5** | rs352900653 | 17,838,414 | 0.1137 | 1.52 | 0.1416 | 1.31 |
| **SNP6** | rs349680813 | 17,838,420 | 0.3207 | 0.60 | 0.3500 | 0.53 |
| **SNP9** | rs21567139 | 17,838,643 | 0.9213 | 0.01 | 0.8981 | 0.01 |
| **SNP10** | NA | 17,838,646 | 0.0604 | 2.13 | 0.0643 | 2.06 |
| **SNP11** | NA | 17,838,669 | 0.0605 | 2.52 | 0.0522 | 2.47 |
| **SNP13** | NA | 17,838,672 | 0.2466 | 0.82 | 0.2499 | 0.80 |
| **SNP16** | NA | 17,838,698 | 0.6811 | 0.10 | 0.5670 | 0.20 |
| **SNP2** | rs21567149 | 17,839,130 | 0.3372 | 0.56 | 0.3303 | 0.58 |
| **SNP20** | NA | 17,839,873 | 0.5121 | 0.26 | 0.5204 | 0.25 |
| **SNP21** | NA | 17,839,883 | 0.7450 | 0.06 | 0.6236 | 0.15 |
| **SNP22** | NA | 17,839,879 | 0.6492 | 0.13 | 0.6894 | 0.10 |
| The association analysis was conducted using STRUCTURE 2.2 and TASSEL 2.1. ∗∗ indicates significance at the α = 0.01 probability level. | | | | | | |
